# Supplementary material for: Evolutionary conservation and in vitro reconstitution of microsporidian iron–sulfur cluster biosynthesis
Source: Nat Commun. 2017 Jan 4;8:13932. doi: 10.1038/ncomms13932 (PMC5216125; doi:10.1038/ncomms13932)
Supplement: Supplementary Information — Supplementary Figures, Supplementary Tables and Supplementary References [file ncomms13932-s1.pdf]

Supplementary Figure 1: Conservation of amino acid residues in microsporidian ISC and CIA proteins.

Multi-sequence alignments are provided for **a**) the ferredoxin oxidoreductase Arh1, **b**) the ferredoxin Yah1, **c**) the monothiol glutaredoxin, **d**) the ABC transporter Atm1, **e**) the metal-binding P-loop NTPases Cfd1 and Nbp35, **f**) the CIA targeting complex protein Cia1, **g**) the CIA targeting complex protein Cia2, **h**) the iron-only hydrogenase-like CIA factor Nar1, and **i**) the electron transfer chain components Dre2 and **j**) Tah18.

a. Ferredoxin oxidoreductase Arh1

```
Arh1_Tra_hom    M------------------------------------------KICIIGA
Arh1_Enc_cun    M------------------------------------------KVCVIGG
Arh1_Enc_int    M------------------------------------------KVCVIGG
Arh1_Nos_cer    M------------------------------------------KVAIIGG
Arh1_Ent_bie    M-----------------------------------------KTVAIIGS
Arh1_Sac_cer    MSF---------------------------VQIRHISSQINRKTVSIVGS
Arh1_Neu_cra    MTFVARVKPGALGLRGRWPSQVHAPRRMYSQHPAHHTSPEHPLRVAVIGS
Arh1_Hom_sap    MASRC----WRWWGWSAWPRTRLPPAGSTPSFCHHFSTQEKTPQICVVGS
Arh1_Cry_par    MRP-----------------------------------YKPIYKLCIVGA

Arh1_Tra_hom    GPSTYYLTNHIHTHAP----STTIHVFEKNSSPLQSLKLFHTKPT-MR--
Arh1_Enc_cun    GPAGLYTAASLLARN------IDVTLHEKEAEVGGMYR-YSLLPASK---
Arh1_Enc_int    GPAGLYTAASLLSRD------VNVTLYEKEPELGGLYR-YSLLPESR---
Arh1_Nos_cer    GPSGLFLAKYLSKHA------NSITIYEKSGTFGGMYN-YSYNPKL----
Arh1_Ent_bie    GPSGLLVANYLANY-------MKINIYEASNKILGHYN-YSLNIKK----
Arh1_Sac_cer    GPSGFYTAYHLLKKSP---IPLNVTIWEKLPVPFGLSR-YGVAPDHPEVK
Arh1_Neu_cra    GPAGFYTTYKLMAKIQ----GTKVDMYESLPVPFGLVR-FGVAPDHPEVK
Arh1_Hom_sap    GPAGFYTAQHLLKHPQ-----AHVDIYEKQPVPFGLVR-FGVAPDHPEVK
Arh1_Cry_par    GPSGCYLAKYLLARSKKENIAIKIDLLDSLDKPFGLLR-YGIAPDRHDLK

Arh1_Tra_hom    -----INNVL--------------CNIKVHK-----------WMFERLSA
Arh1_Enc_cun    --MSPFAKLL---EH---KNFSLKLNSKVDLG-----------KLKTMEK
Arh1_Enc_int    --MSPFTKLL---EH-----KNFSLKLNSKIDL---------EKLSGMEK
Arh1_Nos_cer    ---NIFKNIL---NT-----KNIDFKPNFEITES---------NFKTIEP
Arh1_Ent_bie    ---SLFENIL---KN-----KNINLFLNTKIT-----------DLTTI--
Arh1_Sac_cer    NCEETFTTCAEEFSSPTNQKHKFSF---VG-GITIGKEILLKELL----D
Arh1_Neu_cra    NCQEKFEEVA---SS-----PDFRFIGNVSVGTKSDHPDGLTVPLASLFR
Arh1_Hom_sap    NVINTFTQTA---HS-----GRCAFWGNVEVGRDV--------TVPELRE
Arh1_Cry_par    KSISSIDNSLFKKYS-----DDIKFYGNVTLGYDV--------KLEELKR

Arh1_Tra_hom    FYDGIVVAVGGRARR-LD-------GA---LN-KFAVYGEDVVRKGGGLE
Arh1_Enc_cun    EFDAFVIATGSDGPRRLD-----IPGG------EHCVSSLDIAKSWTG--
Arh1_Enc_int    EFDAFVIATGPGGPRKLA-----IPGA------DHCIGSLDIAKSWAG--
Arh1_Nos_cer    YYDKFVLAIGGVPNF-NE--------N------SSYINALDIIKNK----
Arh1_Ent_bie    NADAIILATGGIAKS-IN----------------GTKTALDIIKQYDH--
Arh1_Sac_cer    NQDAVILSYGCTGDRKLN-----IPGE---LGTKGVFSSREFVNWYNGHP
Arh1_Neu_cra    HYNAIIFAYGAAQDRKLG-----IPGE---DQLKGVYSAREFVGWYNGLP
Arh1_Hom_sap    AYHAVVLSYGAEDHRALE-----IPGE---E-LPGVCSARAFVGWYNGLP
Arh1_Cry_par    KYDVVVLAVGGLQSF-HTLPVKYMNNELQNKIIGGVFSSRDWVFYYNSHP

Arh1_Tra_hom    GRTG------------RSARLLESVLESKRMLGRGRRSAEAMGLEKKNKI
Arh1_Enc_cun    --------------------------------------------------
Arh1_Enc_int    --------------------------------------------------
Arh1_Nos_cer    --------------------------------------------------
Arh1_Ent_bie    --------------------------------------------------
Arh1_Sac_cer    DFAK------------------DKRF------------------------
Arh1_Neu_cra    QH----------------------AN------------------------
Arh1_Hom_sap    EN----------------------QE------------------------
Arh1_Cry_par    MFKKMLYPTKNEHSNSINNKLIDNERIDMDIELNYLKKKNNQFFEYKSPL
```

```
Arh1_Tra_hom    TKNKNNEENENTRKD--VNSREMHESLVHAKPFSRLRRVAIVGAGNVSLD
Arh1_Enc_cun    -------------EELR---------------YTVGRKVLVVGMGDVSMD
Arh1_Enc_int    -------------EEPK---------------YTVGSKVLIVGMGDVSMD
Arh1_Nos_cer    --------------VSI---------------NSLGKKVCIIGMGNVALD
Arh1_Ent_bie    --------------NQD---------------VNIGKNICIIGAGNVAMD
Arh1_Sac_cer    -------------TDFD---------------WSKVSKVGIIGNGNVALD
Arh1_Neu_cra    -------------LNPD---------------LTQGEEAVVIGQGNVALD
Arh1_Hom_sap    -------------LEPD---------------LS-CDTAVILGQGNVALD
Arh1_Cry_par    SSTEISVPFKN--ENEDFKYGYGSDILRNYILNSSERNAVIIGNGNVSLD

Arh1_Tra_hom    VANMLYE---------------------------------QGVPQIFILS
Arh1_Enc_cun    IARFLFGWQ------GPQFRFPKSILEKV-----------KDVEDVTITS
Arh1_Enc_int    ITKYLFGWA------GPQFKFPKNALERV-----------KEVRDVTITS
Arh1_Nos_cer    VCRKII----------------------------------TKVNEIDIIS
Arh1_Ent_bie    LLLRLK----------------------------------NKLSTATVIS
Arh1_Sac_cer    ITRVLISNQIDEIWENTDISSLALNLLRR-----------APVKDVKLIA
Arh1_Neu_cra    VARMLLEDV--DVLRKTDIAAHALETLSQ-----------SRVKRVHVVG
Arh1_Hom_sap    VARILLTPP--EHLERTDITKAALGVLRQ-----------SRVKTVWLVG
Arh1_Cry_par    ITRLLSFYTHEQLSKNKYLNPDYLNLIDTSSKYSNSDIYRPLFKNIFIIG

Arh1_Tra_hom    RNELAKCAFGSYELRRCMHDKNVRVWGGTVGNDRRGMI-VGERLRKCEK-
Arh1_Enc_cun    RRGVSGSAFTNSGLRSVLEIPDLGFHWSEGGNTPQNHRPRPENFEETSN-
Arh1_Enc_int    RRNVLESSFSNHGLRSVLEIPRLGFSWSSPKDISCPDKDKTKDLEQKFT-
Arh1_Nos_cer    RGGLYISKFGNNVMREILNLANFKGH----------NISLPSNLKEN---
Arh1_Ent_bie    RTDLFNSKFTNNKLRNIKDYYQITTNIYN-INDL-----QPKNNEDI---
Arh1_Sac_cer    RRDFVHSKFTNKELRELWELEKYGIRGRIDPKFFQK--------------
Arh1_Neu_cra    RRGPMQAAFTIKEVRELMKLSNVSF----HPVDTSL--------------
Arh1_Hom_sap    RRGPLQVAFTIKELREMIQLPGARPI-LDPVDFLG---------------
Arh1_Cry_par    RRGWIQNSFKYPLLKEFIDKSRKSKYNSTNGMNIRVMM-SQEDFELSQD-

Arh1_Tra_hom    ---DS-----------GDEEIIETE-NESSRNSTKIENNKIANDTRCDDP
Arh1_Enc_cun    ---WAREHR-----DSEENGRIKK---WWERRM----G--------LLG-
Arh1_Enc_int    ---YLQKDD-------GKKDNINK---WKERRL----R--------LFQ-
Arh1_Nos_cer    -------------------------------RRY----N--------LLN-
Arh1_Ent_bie    -------------------------------RRL----H--------IIK-
Arh1_Sac_cer    ---------------------EMFDPSKYDRAFNRRVEMCSEYLKPFNER
Arh1_Neu_cra    ---------------------LPPDLKSLPRAPRRLMEMLAKGTTAISQS
Arh1_Hom_sap    ---------------------LQDKIKEVPRPRKRLTELLLRTATE-KPG
Arh1_Cry_par    ---RT-----------SLFELERSG-PEIKRRFLKMKSIFQEMVNNHQEY

Arh1_Tra_hom    FYTSPTLLSLFNLSYNPTETSFHNQSPSLQPKQTLYLLLNTVLQSIKKNV
Arh1_Enc_cun    ---AVRKG------------------ARRLRLMFNTNIKSIEKVGAQYKV
Arh1_Enc_int    ---GVREG------------------AKRLRLMFNTNIKSIERVGRQYKV
Arh1_Nos_cer    ---NNNIKIDK--------------TKNNINLFFDTSIKKVTKINGKYVV
Arh1_Ent_bie    ---KINNNIN---------------NPKISFIFNGTVKQIADNKVTY---
Arh1_Sac_cer    SKKNYKKAPPPSSGYDKFWELDYLKTPLKINRDDFGAIN-----------
Arh1_Neu_cra    -PS----------ETAKSWSLDFCLTPKAFSPSSSSSTP-----------
Arh1_Hom_sap    -----PAEAARQASASRAWGLRFFRSPQQVLPSPDGRRA-----------
Arh1_Cry_par    IANNDNIFHNDKTINIHFKNLFSTVNIKTEEVNIPENNVKKK--------
```

```
Arh1_Tra_hom    SLFNLALK------------------TR-E--GTKMLNNVDLVVNCTGYT
Arh1_Enc_cun    KME---------------------------QDGVQIEECFDSVISSIGFN
Arh1_Enc_int    KME---------------------------QGGIPIEEYFDTVISSVGFS
Arh1_Nos_cer    EFN---------------------------N---GCEHKYDSIITSFGFK
Arh1_Ent_bie    ---------------------L-----S-N--GETVEKIFTDIISSIGFI
Arh1_Sac_cer    S-LSLCNNRLNE-----D-----NSLQPLKDVNNIMTYKVDLLITSLGYA
Arh1_Neu_cra    TSTQLASTTFER--TTLSPSPFDPNAYA-LPTGETLTLPSSIAFRSIGYK
Arh1_Hom_sap    AGVRLAVTRLEG--VD-------EATRA-VPTGDMEDLPCGLVLSSIGYK
Arh1_Cry_par    SIP--FIKGIELARNIRDSKPI-TDKTK-LNEKEKYYLPCQLLITSLGFK

Arh1_Tra_hom    GRDL-----STYV------------------------------CTKPLYF
Arh1_Enc_cun    RTKE-----KAPG------------------------------IAKAVYH
Arh1_Enc_int    RADP-----RSLG------------------------------FTKPVYY
Arh1_Nos_cer    ANQL------KIN------------------------------TDKPVYK
Arh1_Ent_bie    PNIN-----VN--T--------------------------NTVNNVPVYK
Arh1_Sac_cer    GVPMPEFSKLSIGFDK--DHIANK-QGRVL--------TSSGEIFPHLYA
Arh1_Neu_cra    STPLPEFSDINIPFDERRGIISNDGRGRVQHEERTRGAEMSHGSFPGLYC
Arh1_Hom_sap    SRPV----DPSVPFDSKLGVIPNV-EG-------------RVMDVPGLYC
Arh1_Cry_par    PKYD-----YIFNGNKDY-------------------SFENNCFPCPIFK

Arh1_Tra_hom    LGWCKD-AKGNLSVVKGRAVELGDRMIDEMGL------------------
Arh1_Enc_cun    VGWARH-PRGNVERAKEDAQDVVNKIVQTEKI------------------
Arh1_Enc_int    VGWAKH-PKGNAERAKEDAQDVVNKIVEMKK-------------------
Arh1_Nos_cer    IGWCDI-PFGNISDAVQSAKKMVYKIL-----------------------
Arh1_Ent_bie    IGWCNK-PMGNIASLRINAQILADQIKTVLL-------------------
Arh1_Sac_cer    SGWIRKGSQGVIASTMQDAFEVGDRVIQDLVVSGALSLENSIDLS-----
Arh1_Neu_cra    AGWVKTGPTGVIASTMENAFATADAIIEDWVSRTPFLNADRNVHGWEGVK
Arh1_Hom_sap    SGWVKRGPTGVIATTMTDSFLTGQMLLQDL--KAGLLPSGPR-PGYAAIQ
Arh1_Cry_par    TGWMETNSKGDLNIALQKSLTLGNEILSLLKKMP----------------

Arh1_Tra_hom    --------------------------------------------------
Arh1_Enc_cun    --------------------------------------------------
Arh1_Enc_int    --------------------------------------------------
Arh1_Nos_cer    --------------------------------------------------
Arh1_Ent_bie    --------------------------------------------------
Arh1_Sac_cer    --NIK------HTTWKDWERINKKELLRGKKEHKTRSKFLTFEELWNGVE
Arh1_Neu_cra    SEVLKSGDDKRVVDWQGWRRIDEAERDRGRETGRSREKFTRTGEMLN--V
Arh1_Hom_sap    ALLSS--RGVRPVSFSDWEKLDAEEVARGQGTGKPREKLVDPQEMLR-LL
Arh1_Cry_par    ---------------------------------------------PK-NV

Arh1_Tra_hom    -K
Arh1_Enc_cun    -Q
Arh1_Enc_int    -M
Arh1_Nos_cer    -T
Arh1_Ent_bie    -K
Arh1_Sac_cer    GI
Arh1_Neu_cra    LG
Arh1_Hom_sap    GH
Arh1_Cry_par    EA
```

Abbreviations: Tra_hom, *Trachipleistophora hominis*; Enc_cun, *Encephalitozoon cuniculi*; Enc_int, *Encephalitozoon intestinalis*; Nos_cer, *Nosema ceranae*; Ent_bie, *Enterocytozoon bieneusi*; Sac_cer, *Saccharomyces cerevisiae*; Neu_cra, *Neurospora crassa*; Hom_sap, *Homo sapiens*; Cry_par, *Crystosporidium parvum*.

Conserved residues situated close to the prosthetic group FAD are highlighted in yellow[1,2]. Conserved residues situated close to the cofactor $NADP^+$ are highlighted in red.

In bold: Consensus sequence of four highly conserved peptide segments in Arh1 homologues. All of these polypeptide motifs map to the active-site of Arh1 and make contacts with both FAD and NADP. Three of the motifs are involved in binding FAD: 1) [VI]-[VI]-G-X-G-P; 2) G-L-X-R-X-G-X-A- P-D-H-X(3)-[KR] note that this motif is not conserved in microsporidia; and 3) G-W-X(3)-G-X(2)-G. The last motif is involved in binding NAD: G-X-G-N-V-X(2)-D- X(2)-R[3].

The Arh1-Yah1 complex displays a highly charged surface arising from interacting surfaces that are predominantly acidic (Yah1) or basic (Arh1). In green: basic residues from Arh1 involved in salt bridges with acidic residues of Yah1[4].
Dark green: Basic Amino acids from Arh1 that share properties with those residues shown empirically to interact with Yah1. The Arg 239 and 243 from the human sequence (underlined) were experimentally shown to have binding affinity for Asp76 and Asp79 of ferredoxin[5,6].

b. [2Fe-2S] ferredoxin Yah1

```
Yah1_Tra_hom  M-LKNV--------------------------------------------
Yah1_Enc_cun  MDMFSA--------------------------------------------
Yah1_Enc_int  MDMANA--------------------------------------------
Yah1_Sac_cer  MLKIVTRAGHTARI-------SNIAAHLLRTSPSLLTR-TT---------
Yah1_Neu_cra  MSTPRVLRQSLQRLAQHARCYSKTTTAPLRTQPQRLPTAWS---------
Yah1_Hom_sap  MAAAGGAR-------------------LLRAASAVLGG-PAGRWLHHAGS
Yah1_Gia_int  MSLLSS--------------------------------------------
Yah1_Tri_vag  MLASIS--------------------------------------------
Yah1_Cry_par  MVNNLIWR----IS-------R-------------------PISSRVFSA

Yah1_Tra_hom  -----------------------------KDE--KLINFIFLDK--TPKE
Yah1_Enc_cun  -----------------------------PDRIPEQIRIFFKTM-KQVVP
Yah1_Enc_int  -----------------------------PDKTSGKVGLLFKTM-GKMIP
Yah1_Sac_cer  ---------TTTRFLPFSTSSFLNHGHLKKPKPGEELKITFILKDGSQKT
Yah1_Neu_cra  TTTQLSASASASARRSLSTSSALQHGHVDPPKPGEELYVTFIDKDNQTHR
Yah1_Hom_sap  RAGSSGLLRNRGPGGSAEASRSLSVSARARSSSEDKITVHFINRDGETLT
Yah1_Gia_int  --------------------------IRR-----F-ITFRVVQQ-GVEHT
Yah1_Tri_vag  ---------------------------RS------AVKIHWTGK-GCDKI
Yah1_Cry_par  IPYFSKRTLFLSFKRFFHSDPELW-----TKDVHPKIELSFILRDGEKKV

Yah1_Tra_hom  VFSVPGKTLLEVAHANKIDLE--GACEGSLACSTCHVIL-DKKLYNSLEE
Yah1_Enc_cun  AKAVCGSTVLDVAHKNGVDLE--GACEGNLACSTCHVIL-EEPLYRKLGE
Yah1_Enc_int  VNAVYGDTVLETAHKNGVDLE--GACEGNLACSTCHVIL-EEPLYRRLGE
Yah1_Sac_cer  YEVCEGETILDIAQGHNLDME--GACGGSCACSTCHVIV-DPDYYDALPE
Yah1_Neu_cra  LAVSEGDNLLDIAQAHDLEME--GACGGSCACSTCHVIVQDQDMYDRMPE
Yah1_Hom_sap  TKGKVGDSLLDVVVENNLDIDGFGACEGTLACSTCHLIF-EDHIYEKLDA
Yah1_Gia_int  VSGAVGQSLLDAIKAAHIPIQ--DACEGHLGCGTCGVYL-DKKTYKRIPR
Yah1_Tri_vag  VEGHNGETLLKIAERNKLPLP--NACEGNRACATCQVYV-NKG-GDLLNE
Yah1_Cry_par  FNAPKNISLLEAAQHEELDIE--GACEASLACSTCHVIL-DKEIYDELEP

Yah1_Tra_hom  PSDREYDLLEQAFMPCNTSRLGCQVRVDERLRNSTIKLPRATRNMAVDG-
Yah1_Enc_cun  PSDKEYDLIDQAFGATGTSRLGCQLRVDKSFENAVFTVPRATKNMAVDG-
Yah1_Enc_int  PSDKEYDLIDQAFGITSTSRLGCQLKIDKSFEKTVLTIPRATKNMAVDG-
Yah1_Sac_cer  PEDDENDMLDLAYGLTETSRLGCQIKMSKDIDGIRVALPQMTRNVNNND-
Yah1_Neu_cra  PDDDENDMLDLAFGLTETSRLGCQVHMTKELDGLVVKLPSMTRNLQASD-
Yah1_Hom_sap  ITDEENDMLDLAYGLTDRSRLGCQICLTKSMDNMTVRVPETVADARQSI-
Yah1_Gia_int  ATKEEAVLLDQVPNPKPTSRLSCAVKLSSMLEGATVRIPSFNKNVLSESD
Yah1_Tri_vag  ISDAEYDTLDYAVDLREQSRLACTCVLQTDDGEMDVVIPERCRNIDVSE-
Yah1_Cry_par  PSEREEDMLDMAPQVCETSRLACQIKVDERLTKGNIHLPNMTRNFYVDG-

Yah1_Tra_hom  -----FKP--QPH
Yah1_Enc_cun  -----FKP--KPH
Yah1_Enc_int  -----FKP--KPH
Yah1_Sac_cer  -----FS------
Yah1_Neu_cra  -----FKS-----
Yah1_Hom_sap  -----DVG--KTS
Yah1_Gia_int  ILASEEKKRHGQH
Yah1_Tri_vag  -----FKKKKSIL
Yah1_Cry_par  -----FKP--SPH
```

Abbreviations: Tra_hom, *Trachipleistophora hominis*; Enc_cun, *Encephalitozoon cuniculi*; Enc_int, *Encephalitozoon intestinalis*; Sac_cer, *Saccharomyces cerevisiae*; Neu_cra, *Neurospora crassa*; Hom_sap, *Homo sapiens*; Gia_int, *Giardia intestinalis*; Tri_vag, *Trichomonas vaginalis;* Cry_par, *Crystosporidium parvum*.

Residues involved in coordination of the [2Fe-2S] cluster are highlighted in red[7,8]. Acidic residues that interact with Arh1 are highlighted in green[6]. The glutamic acid residue (D to E change) in ThYah1 is highlighted in dark green. The replacement of D76 (underlined) with E in human Yah1 resulted in a strong increase in the Km value while replacement of D79 with E (as in the *T. hominis* sequence) exhibited only a slightly reduced binding affinity[9]. The two-conserved acidic regions are in **bold**. Ferredoxins containing a [2Fe-2S] cluster comprise two major groups: the plant and the vertebrate-type. They exhibit distinctive biochemical and structural properties. The vertebrate type is present in almost all living cells except Archaea. The amino acid residues that distinguish the vertebrate type from the plant type are highlighted in light blue.

c. Monothiol glutaredoxin Grx

```
Grx3_Tra_hom     MDRSPHELSAEEKKNEDELNNIFFKLQNYTNIIAHENDE-QEIDALL--K
Grx5_Enc_cun     MALSQPL-----KMEVLDHNDAFEELKEYDVVVGYESES-NELCNML--R
Grx3_Enc_int     M-------------NGIEDSNPFEELKEYEVVVAYDDEN-KKLSNVM--K
Grx3_Nos_cer     MEYNK--------------KKNTFADIDYDNIVLF-------YENEI---
Grx3_Ent_bie     MDLKN--------------ICDTNDFVLFYKENNTAFEN---LNTQI-KE
Grx5_Sac_cer     MFLP-----------KFNPIRSFSPILR--------------AKTLL--R
Grx5_Neu_cra     MLTR-----------SLFSRQLFAAASRPAIAPKAVSSA---FRPVL--F
Grx5_Hom_sap     M--------------SGSLGRAAAALLRWGRGAGG-GGL---WGPGV--R
Grx5_Gia_int     MDQINGGL----FPKALALTRYASRGLILGIISAAGAELENQLAGFLTRR
Grx3_Cry_par     MSTTLNSF---VGITDFVLGNCLSAILILSGKEEIDGSL-LELENVLQES
```

```
Grx3_Tra_hom     YIDDDEYIIVNLSISPVLKERFMKTYSVEKLPILISYSTNI---------
Grx5_Enc_cun     NAGVD-YVEVNLGESERLKAEFMKHFNVSKLPVLIIKGSPVS--------
Grx3_Enc_int     DRGLD-YVEVNLAVSEKLRTEFMKHFRVSELPVLLIEGIPAL--------
Grx3_Nos_cer     PPGMEQNIRIINCSKQELRNAVVARYNLETLPALLFYKKVIY--------
Grx3_Ent_bie     FDDVA-YVD--LSISKKLEELVSKTFNVNSFPLLIFKNTKI---------
Grx5_Sac_cer     YQ------------------------------------------------
Grx5_Neu_cra     YQ------------------------------------------------
Grx5_Hom_sap     AA------------------------------------------------
Grx5_Gia_int     YPRSL-FLPNAQ--NSVSRFLM---------PVCAPLATVS-------RG
Grx3_Cry_par     FSNVK-FGKISP--SGVNEISAIKQFDVKELPSILLFTCQSLKPYKVIS-
```

```
Grx3_Tra_hom     --YNDENVDAY--LKERKVNEDSFIDHKIDKMVGEKKVMVFIKGSPDKPE
Grx5_Enc_cun     -GDPSDKIREY--AE----EREGDILRRIQSTVDPKRVTLFIKGSPENPK
Grx3_Enc_int     -DDPSERIKEY--IE----RKEKKSLEKIQSVIDPNKITLFIKGSPEHPR
Grx3_Nos_cer     --LKNDNIKNY--VE----DKQLLLEREVKRIINSCKIVLFIKGDLFDPY
Grx3_Ent_bie     --TPKDTVENV--L-------YNYYFKFCKEFVCQSKYVFFMKGTIEKPY
Grx5_Sac_cer     -------------NR---MYLSTEIRKAIEDAIESAPVVLFMKGTPEFPK
Grx5_Neu_cra     -------------NR----FLSDATRQAIDKAVASAPVVLFMKGTPETPQ
Grx5_Hom_sap     -------------GS---GAGGGGSAEQLDALVKKDKVVVFLKGTPEQPQ
Grx5_Gia_int     LERTSDLNATLAQSESIKQELKMFILPQIRELLAENPVVLFMKGTPDSPE
Grx3_Cry_par     GYNPSELHTNLEELIKIQNLSIPSQNEKFKILTNFKSLMVFMKGIKEEPY
```

```
Grx3_Tra_hom     CKFTKELISHFDELQLKNGKDYSYFNIKLDNKTRNRLKKRNNWPTFPQIY
Grx5_Enc_cun     CGFTKTLMDILYSAGVT-KDQIVYFDILSDEDVRRKLKEINSWPTFPQVY
Grx3_Enc_int     CGFTKSLIEILYGLGVT-RDKIEYLDVLSDEDIRERLKEINRWPTFPQVY
Grx3_Nos_cer     CHFSKEVIQILKDNNVN-LDEIVYYNVLKNKEMAEKIKEVNKWPTFPQLF
Grx3_Ent_bie     CKYSKQLVELCNKKNI---IDIIAFDIFQDNIMREYLKKINNWPTYPMIF
Grx5_Sac_cer     CGFSRATIGLLGNQGVD-PAKFAAYNVLEDPELREGIKEFSEWPTIPQLY
Grx5_Neu_cra     CGFSRASIQVLGLQGVD-PNKFAAFNVLEDAELRQGIKEYSDWPTIPQLY
Grx5_Hom_sap     CGFSNAVVQILRLHGV---RDYAAYNVLDDPELRQGIKDYSNWPTIPQVY
Grx5_Gia_int     CGFSKFASMLLKYNNI----SFVGVDVLDDPALRQGIKLYGNWPTIPQLY
Grx3_Cry_par     CRFAKGLVSLLDSIKV---KNYGHYNIFENEETRQGLKEYHNWPTFPQIC
```

```
Grx3_Tra_hom     IDKLFVGGLDTFKKMKEKKIVQKMLFPGDQ-------------EEIE
Grx5_Enc_cun     IGGRFIGGLDVVRKMSEKGELRREIQ-----------------EII
Grx3_Enc_int     IRGRFIGGLDIVRKMSEKGKLKDELS-----------------GII
Grx3_Nos_cer     VNGKLIGGCDILKKLNETKELTKIL------------------NKQ
Grx3_Ent_bie     VDGQFIGGLDAFTDIIRC-------------------------DKI
Grx5_Sac_cer     VNKEFIGGCDVITSMARSGELADLLEEAQALVPE-----EEEETKDR
Grx5_Neu_cra     IDKEFVGGCDIIVSMHQNGELAKLLEEKDVLVKGEEGAAEEQTEKKE
Grx5_Hom_sap     LNGEFVGGCDILLQMHQNGDLVEELKKLGIHSALL---DEKKDQDSK
Grx5_Gia_int     VKGELIGGSDIIQQLHESGELRKVCG-----------------LPD
Grx3_Cry_par     INGEFIGGLDILNEMHSNGELVNEIPK----------------DAF
```

Abbreviations: Tra_hom, *Trachipleistophora hominis*; Enc_cun, *Encephalitozoon cuniculi*; Enc_int, *Encephalitozoon intestinalis*; Nos_cer, *Nosema ceranae*; Ent_bie, *Enterocytozoon bieneusi*; Sac_cer, *Saccharomyces cerevisiae*; Neu_cra, *Neurospora crassa;* Hom_sap, *Homo sapiens; Gia_int, Giardia intestinalis;* Cry_par, *Cryptosporidium parvum*.

Monothiol glutaredoxins bind a [2Fe-2S] cluster in a bridging fashion. The iron atoms are coordinated by the cysteine in the active site CGFS (in yellow) of the protein and a cysteine from bound glutathione. Monothiol glutaredoxins from bacteria and Grx3 and Grx4 from yeast form homodimers and it has been proposed that switching from dimeric to monomeric conformation releases the [2Fe-2S] cluster to acceptor proteins. In human, the holo-GLRX5 is tetrameric, whereas the metal free protein is also monomeric[10-13]. The amino acids involved in inter-subunit interaction in human Grx5 are shown in green. The amino acids in pink stabilize a loop in the tetrameric structure that shields the [2Fe-2S] cluster. In light blue and **bold**: essential residues for the biological activity of yeast Grx5[14]. The glutaredoxin domain (PF00462) is underlined in blue. The N-terminal thioredoxin-like domain (SSF52833) identified in *E. cuniculi* and *E. intestinalis* glutaredoxins using HHPred (http://toolkit.tuebingen.mpg.de/hhpred) [15] is highlighted in red. A CDD search[16] identified a thioredoxin-like domain (cd02984) in the protein from *C. parvum* (red). By contrast, no thioredoxin-like domain was identified in the corresponding segment of the *T. hominis* and *G. intestinalis* sequences.

d. ABC transporter Atm1

```
Atm1_Tra_hom1    MAS-----------------------------------------------
Atm1_Tra_hom2    M-------------------------------------------------
Atm1_Tra_hom3    MQGSNNAYRN----------------------------------------
Atm1_Enc_cun1    MVICFRSPFK---QVNKYFR------------------------------
Atm1_Enc_int1    MGG-----------------------------------------------
Atm1_Nos_cer1    MKK-----------------------------------------------
Atm1_Nos_cer2    MLE-----------------------------------------------
Atm1_Nos_cer3    MKE-----------------------------------------------
Atm1_Sac_cer     MLLLPRCPVI-----GRIVR-SKFRSGLIRNHS-----------------
Atm1_Neu_cra     MAPSIKLSTM-----ATSLHRAHGTSALLRRPR--------LWAPRLSSI
Atm1_Hom_sap     MALLAMHSWRWAAA-AAAFE-KRRHSAILIRPLVSVSGSGPQWRPHQLGA
Atm1_Tri_vag     MES-----------------------------------------------
Atm1_Cry_par     MWAFTQKASN--L--K-ILK---PHPFVLNRST-----------------

Atm1_Tra_hom1    --------------------------------------------------
Atm1_Tra_hom2    --------------------------------------------------
Atm1_Tra_hom3    ----------------------------------------SSYVYDRLCD
Atm1_Enc_cun1    --------------------------------------------------
Atm1_Enc_int1    --------------------------------------------------
Atm1_Nos_cer1    --------------------------------------------------
Atm1_Nos_cer2    --------------------------------------------------
Atm1_Nos_cer3    --------------------------------------------------
Atm1_Sac_cer     ---PVIFTV-SKL-STQRPLLFNSAVNLWNQAQKDITHKKSVEQFSSAPK
Atm1_Neu_cra     HATPTIANLRASF-TTSSPRLFAPNGSAKDESK--PAVSTVPKTTGRGPS
Atm1_Hom_sap     LGTARAYQIPESLKSITWQRLGKGNSGQFLDAA--KALQVWPLIEKRTCW
Atm1_Tri_vag     --------------------------------------------------
Atm1_Cry_par     --KYGILYLGVFATSIRGKRAFFSSESNFAQGNCMHAMTTSTFKNKDLAK

Atm1_Tra_hom1    -------------I-----TTYEILQIIMTKYVK--DIPLIR-MTIMPTL
Atm1_Tra_hom2    ----------------------NIHLYIFKRYVI--LNTTLA-LFMPPIL
Atm1_Tra_hom3    IACTYTDTIKKLCERSPHMSKPFQIFIFVVKEMF--SPPLKPCFVIFTIV
Atm1_Enc_cun1    --KISASMGGKKKV-----SELQTLSIIVRKYIL--SIPQVR-IIVFPVL
Atm1_Enc_int1    ----------KKKV-----SELQTLSIIVRKYIL--GIPQAR-IIVFPVL
Atm1_Nos_cer1    -------------I-----SNRKIIKEIIYNDLL--KIRMLR-YVIVPII
Atm1_Nos_cer2    ----------KKSI---YGSDISIMKDLIVEYVI--SKPFIR-TFVIYII
Atm1_Nos_cer3    -------------K-----TNYEILYDTFVKYCY--NISYIR-YVMFPIL
Atm1_Sac_cer     VK-----TQVKKTSKAPTLSELKILKD-LFRYIWPKGNNKVR-IRVLIAL
Atm1_Neu_cra     DP--L--AAIDKTAQEQRKADWAIMKE-MSKYLWPKGSWGDK-ARVLLAI
Atm1_Hom_sap     HGHAGGGLHTDPKEGLKDVDTRKIIKA-MLSYVWPKDRPDLR-ARVAISL
Atm1_Tri_vag     ----------------------DVTYI-----------------------
Atm1_Cry_par     EN---KLVNILKKSRFIQEKDSKNIEI-LTKYLWP-KNREYR-KRIIFSL
```

```
Atm1_Tra_hom1    FLMFVARIMEVKVSEIVQNASLEFS-------G----------YREGGT
Atm1_Tra_hom2    TCIILSKYLKIYASSILKDIGQAIE-------D----------SHTVSRL
Atm1_Tra_hom3    AIIFITELLLYKIRIENLRSLSSCK-------NYSTLRSFLF-YTTICGI
Atm1_Enc_cun1    LSIFAGKYFEVKVASCISHISDGLD-------S----------EGIPRGR
Atm1_Enc_int1    LSIFAGKYFEVKVASCISYISEGLD-------L----------EEVPRSR
Atm1_Nos_cer1    FLTIFYTVLEIKISTLVKNLNQSIA-------N----------RKGEHTA
Atm1_Nos_cer2    FGTFAVCTFNVLTVEATRKLTSDIT-------D----------NKDLSQS
Atm1_Nos_cer3    LSTIIACYLEVQASNISKRIAEDFE-------N----------KINAGKS
Atm1_Sac_cer     GLLISAKILNVQVPFFFKQTIDSMNI------AWDDPTVALP--AAIGLT
Atm1_Neu_cra     GLLVGGKVLNVQVPFYFREIVDSLNI------DFSTTGGSVT--AVAGAM
Atm1_Hom_sap     GFLGGAKAMNIVVPFMFKYAVDSLNQMSGNMLNLSDAPNTVA--TMATAV
Atm1_Tri_vag     --------------------------------DRNSIGDSLN--------
Atm1_Cry_par     LSLGVAKLATIQVPLLLSRLIDNVGA-----ISSSNLSLSLTNKLNVLFL
                  TMD1

Atm1_Tra_hom1    FK--KYMIVGILSSLLIE-LQGFIFKSSVQRAYRTALKSS--LREYLLL-
Atm1_Tra_hom2    II--KLGACYLLYAFFNE-IYDFIAASPIQRAARHASNDF---LRNCLM-
Atm1_Tra_hom3    IYPTKFLVYQVIYSDYLERLSCRAFYTVLSRRWEITLQEEGLNSRFKNL-
Atm1_Enc_cun1    IA--MFLAVSLMGIMFTE-LQGFVFVSAVQYVYRYTLRTT--FEYFIQM-
Atm1_Enc_int1    II--MFLVISLTGIIFTE-LQGFVFVSAIQYVYRYTLRTT--FEYFIRM-
Atm1_Nos_cer1    LE--RFVLQSLLVAVLTE-LNGFIFTGVVQYIYRNTAKST--FKGFISL-
Atm1_Nos_cer2    LL--YFAVLTIVSITMSE-LNNFIFVTPVQHVFRLTGKNS--FKNFINM-
Atm1_Nos_cer3    IF--KYILFLTSSIVLRQ-INDIVFSGPIQFLYKIVGVEA--FYHYISL-
Atm1_Sac_cer     IL--CYGVARFGSVLFGE-LRNAVFAKVAQNAIRTVSLQT--FQHLMKL-
Atm1_Neu_cra     IL--GYGAARVGAVVSQE-LRNAVFASVAQKAIRKVARNT--FEHLLNL-
Atm1_Hom_sap     LI--GYGVSRAGAAFFNE-VRNAVFGKVAQNSIRRIAKNV--FLHLHNL-
Atm1_Tri_vag     --------------LLNE--------DPAQISSVFN------FSKY----
Atm1_Cry_par     IS--SYGIARISSSGFNE-LRNALFSEVSQYACKDLSLKA--FHHFHNVS
                      TMD2

Atm1_Tra_hom1    NWSEFKMKGMGEITASIERRSSAVSEILDVFIINLLPVFFVLFLAVLKIY
Atm1_Tra_hom2    ERVRLYECSSGEVGRTLVRSASAVSDLIDITLLEILPLLITFVLALTEMY
Atm1_Tra_hom3    GWLVESVTDNGIFQSFVHRGVKGMTNLIRQLVLSLLARIFSYFFVMKETN
Atm1_Enc_cun1    ETETFESYGSGTIQSIITRKSKAISDFVEVSVQNLFPVVASLAFVGLEAY
Atm1_Enc_int1    ETEMFESYGSGTIQSIITRKSKAISDFVEVSIQNLFPVVASLTFVGLEVY
Atm1_Nos_cer1    SPKNFSTIGGGEIQTIIDRKSKSASELIEVFLTSLLPICLKLLFALIAVI
Atm1_Nos_cer2    ELSRYNKIGCGEIQTIIDRESKAISELIEVLFVNIINIFFTVILACSSIY
Atm1_Nos_cer3    NLENFNKIGSGEIQMIIERKSRAYGDILELTTLSAVPTCIVVIMSFFSVY
Atm1_Sac_cer     DLGWHLSRQTGGLTRAMDRGTKGISQVLTAMVFHIIPISFEISVVCGILT
Atm1_Neu_cra     DLSFHLSKQTGGLTRAIDRGTKGISFLLTSMVFHIVPTALEISMVCGILT
Atm1_Hom_sap     DLGFHLSRQTGALSKAIDRGTRGISFVLSALVFNLLPIMFEVMLVSGVLY
Atm1_Tri_vag     ----MQLRNIGQIIA--------------------------FVFTGFTIS
Atm1_Cry_par     NLSFIQSHRSGELLTIITRGFKSVSKLLNIMIFQIIPTTAEFLMVLGILL
                                                    TMD3
```

```
Atm1_Tra_hom1    AIMGFTSSLIILISLITYTTVTIKMAVWRNEIRKKLNLSINESNNKLIDI
Atm1_Tra_hom2    RKFGYAAFMYNVMMLIVYIVITFAITRYRMRYRRRNNMYENRAHSKCLEC
Atm1_Tra_hom3    DQLGTTILLAIIFMIVITLLTNIIIIYILIYYRRRYNFLRAQLDNHVSEC
Atm1_Enc_cun1    LRLGVLASLIIILAVGVYGTMTISIALRRNRIRGALNNAENTASNIVYDT
Atm1_Enc_int1    LKLGVLASFIVALAVAAYGFMTISIALRRNRIRGALNSAENAASNIVYDT
Atm1_Nos_cer1    KNMGGLAGFIMFVCVSCYATVTILIAQWRSNIRRELNNSENRSSNKLQDG
Atm1_Nos_cer2    TNLGLTNMFIILITLLVYILATAKIVHWRTGIRKEYNCAQQRCSNHLHDS
Atm1_Nos_cer3    KNLGRDALYVMLATSALYVYFTFVFSIWRNNIRRQYNKSQDKLSNKLQDF
Atm1_Sac_cer     YQFGASFAAITFSTMLLYSIFTIKTTAWRTHFRRDANKADNKAASVALDS
Atm1_Neu_cra     YNFGWQYAALTALTMVSYTAFTILTTAWRTKFRRQANAADNKASTIAVDS
Atm1_Hom_sap     YKCGAQFALVTLGTLGTYTAFTVAVTRWRTRFRIEMNKADNDAGNAAIDS
Atm1_Tri_vag     APLTLFAMFMSIFT--TYIFHKIR-DYAVNNIRAKFK-VNAAAMTVCLET
Atm1_Cry_par     HKVGSEVALITLATMVAYMDFTRRITHKRTIYRKNMNTSEQKSNGLLSDS
                                TMD4

Atm1_Tra_hom1    LSNYESILAFNNQNLELYKYDNKLATSEKHYVKLWRTFYLLNFLQRFVLC
Atm1_Tra_hom2    IRNVDTITVYKTVQFELNQYDDINKKVQFYSSRQYQSLALLNLTQKVILY
Atm1_Tra_hom3    LSNHLLVTYCHKEMDEYQRYKMKVKNYRTAVVMLESVEHVLEILLYLITN
Atm1_Enc_cun1    LSNHESVVSFNNYDIETRRYDAKLMEIERFGTNLFRGLYILNMLQKLIFA
Atm1_Enc_int1    LSNHESVMSFNNYCIEVRRYDGKLMEIERFGTNLFRGLYILNMLQKLIFA
Atm1_Nos_cer1    LNNHETIVSFGTTDLEVDEYDQFLKINASNSNRLWRALYILNLSQRAIFL
Atm1_Nos_cer2    LINHETILAYKTEEEESLKYEKYVSEVESECNRIWRSLSFLYLVNKIIFA
Atm1_Nos_cer3    LANHETIKAYNMEEETITYFDENQKPVEYFGVKSHRILFSLLYIQKMTFA
Atm1_Sac_cer     LINFEAVKYFNNEKYLADKYNGSLMNYRDSQIKVSQSLAFLNSGQNLIFT
Atm1_Neu_cra     LINYEAVKYFNNEAYEVGRYDKALAQYEKNSIKVATSLAFLNSGQNIIFS
Atm1_Hom_sap     LLNYETVKYFNNERYEAQRYDGFLKTYETASLKSTSTLAMLNFGQSAIFS
Atm1_Tri_vag     ISNPRTVYFFDQEERSINKYYTIVDRVCQ-LERIFHGIFSISFGGERTFN
Atm1_Cry_par     LINAETLKYLNGEKYIYDLYSKYQEIYKNSNVKVQTSLAFLNFGQNFIFT

Atm1_Tra_hom1    VQTG-MIIYVG----MCNKITSDQFVLYLSISKILASNLDKLGYMYSRFT
Atm1_Tra_hom2    IFMV-TYLYR-----VRESLGNDQLLQYFSICSTIIEELSNLGCIYHRFS
Atm1_Tra_hom3    ITKF-VVYYV-IYK-NNDVYPVHLVMFVMQKIDVIEQNTKWAGSFYEKLR
Atm1_Enc_cun1    FLNA-SVIALGAYGVLSSKMDGKMLIFYVTTSRILLMNLNNLGFTYCRFT
Atm1_Enc_int1    FLNS-SMIALGVYGLLSSKMDGRVLIFYVTTSRILLINLNNLGYTYCRFT
Atm1_Nos_cer1    LQSF-FIIYAGISGILSQNMSSNQLIYLMSITSTVTINLSNLGYLYTRYT
Atm1_Nos_cer2    LQCF-IVITLGNYGILTAKLSARDVVFYIGINRTLYSSFGQLGFFYSRYT
Atm1_Nos_cer3    IQAI-IIITLGSFGYFPIKLSSQQLVFYISISKTLTNSLSEMGMLYTRYV
Atm1_Sac_cer     TALT-AMMYMGCTGVIGGNLFVGDLVLINQLVFQLSVPLNFLGSVYRDLK
Atm1_Neu_cra     SALT-VMMYMGAHGVATGQLTVGDLVLINQLVFQLSVPLNFLGSVYRELR
Atm1_Hom_sap     VGLT-AIMVLASQGIVAGTLTVGDLVMVNGLLFQLSLPLNFLGTVYRETR
Atm1_Tri_vag     EGTFAVVLSFGGYLVISDRMSGGNLVAMLRIISSFSFLFGLLMGTANNEA
Atm1_Cry_par     GGLL-SAMLITTNKVLAGTLPIGSIVLVTSLLFQLAIPLNFIGMIYRESK
                 TMD5                                  TMD6       ★

Atm1_Tra_hom1    AAILNAKMSF-LDTV-----LPKKLYPIR---------------------
Atm1_Tra_hom2    KAIVDLNTPFVRDMAETNQTYDRKPSVNLTEEPRTKRNTHVGNAVDQYHA
Atm1_Tra_hom3    KAILDAEFAYDFVSSSKN-LKASHDSDLIIAGIGQSRDTCTDHGLSDSHS
Atm1_Enc_cun1    EAMLNAREVLSEDYDLK----TSARLSVA---------------------
Atm1_Enc_int1    EAMLNAREVLSEDYDLK----TNENISMV---------------------
Atm1_Nos_cer1    QAIINARSTYDTMLDIK---KENNKFKIT---------------------
Atm1_Nos_cer2    QAILNIRTSFKPELIKE----EPNLVDVN---------------------
Atm1_Nos_cer3    QGFLNAKSGYYEFKEDT----QTDKIRLI---------------------
Atm1_Sac_cer     QSLIDMETLFKLRKNEVK-IKNAERPLML---------------------
Atm1_Neu_cra     QSLLDMETLFNLQKVNVT-IKEQPNAKPL---------------------
Atm1_Hom_sap     QALIDMNTLFTLLKVDTQ-IKDKVMASPL---------------------
Atm1_Tri_vag     RSMEAANRVFNLLENTQT-VDLKKGLEPE---------------------
Atm1_Cry_par     LTLIDLSKLNEYLTIKPK-NSSNSQCRTI---------------------
```

```
Atm1_Tra_hom1    ------------YF------------------------------------
Atm1_Tra_hom2    RGHEETNNDGRNSNNYAQKGKNICREENINTDQESKNDIQENGRAIPFIN
Atm1_Tra_hom3    DEGEQPAAKRYLETTVTLPSKEIYYDENENVTNTAPSR---------NTK
Atm1_Enc_cun1    ------------RF------------------------------------
Atm1_Enc_int1    ------------RF------------------------------------
Atm1_Nos_cer1    ------------EF------------------------------------
Atm1_Nos_cer2    ------------EF------------------------------------
Atm1_Nos_cer3    ------------DF------------------------------------
Atm1_Sac_cer     ------------PEN-----------------------------------
Atm1_Neu_cra     ------------TLT-----------------------------------
Atm1_Hom_sap     ------------QIT----------------------------------P
Atm1_Tri_vag     ------------SL------------------------------------
Atm1_Cry_par     ------------QLNPKTKVLDFYKEMNL--------------NGTGSSS

Atm1_Tra_hom1    -DKKIVFRNVALPLSSENILNTSNDVFTYAISDNYIFKNMSFEIKKGEKI
Atm1_Tra_hom2    TKPLLQFQNVTIKH-----------------KNTPILTNLTFNVAQYAKV
Atm1_Tra_hom3    GTLIMQFRDFTIIM------------------NQKLLFKPLNLSICNDKI
Atm1_Enc_cun1    -RKNIVFNDVHSYY-----------------GDKKVLRGVNLTIEKGDKV
Atm1_Enc_int1    -GKNIVFKNVRLYY-----------------GDKKILNDVNLTIKKGDKV
Atm1_Nos_cer1    -KESIKFNNLSFGY-----------------ESRQIFNKINLEIYKGEKV
Atm1_Nos_cer2    -NNDIRLENASFNY-----------------YSKKILTDINILIKKGEKV
Atm1_Nos_cer3    -NEKLEFRNVSFSY-----------------LNKPILVNANFVINKGERV
Atm1_Sac_cer     VPYDITFENVTFGY----------------HPDRKILKNASFTIPAGWKT
Atm1_Neu_cra     RGGEIEFKDVTFGY----------------HPESPILRDLSLTIPAGKKV
Atm1_Hom_sap     QTATVAFDNVHFEY----------------IEGQKVLSGISFEVPAGKKV
Atm1_Tri_vag     -KGDIEFKNVWFKYP---------------TRDQWVLKNVSFKINSGDIV
Atm1_Cry_par     KKNSIKLENVSFGFPS-TFGNEHTYIETSNSSDDLVVNDLSLEIPLGKRM

Atm1_Tra_hom1    ALIGPNGIGKSNFLKMLLKF-NEYTGSIKIDDDELCTIDNYSLRDLISYV
Atm1_Tra_hom2    AIVGPNGAGKSSIIKAILKL-TPYSGQI-------QKIENTR----LTYT
Atm1_Tra_hom3    LIKGPNGIGKSSIIRAIFGM-IDYIGDVTIKGVPLQNINTSKLCAMMAIC
Atm1_Enc_cun1    AIVGSNGSGKSTILKTLLKF-NSYQGSICIDGINIKAIENGSFRRTIGYV
Atm1_Enc_int1    AIVGSNGSGKSTILKTFLRF-NRYQGSICIDGISIDAIENGSFRRIIGYV
Atm1_Nos_cer1    AIIGKNGSGKSTLLKLLLKFDEDYKGSILIDDIDIKKIKDEFYRNLLGYI
Atm1_Nos_cer2    AIIGKNGSGKTSLIKMIMRF-ENYGGNIYLDNRDIKEISNSSYRSLISFA
Atm1_Nos_cer3    AIIGKNGTGKSTIIKLLMKF-YKYDGDILIDDTEIDNISDRSYRSLISYA
Atm1_Sac_cer     AIVGSSGSGKSTILKLVFRFYDPESGRILINGRDIKEYDIDALRKVIGVV
Atm1_Neu_cra     AIVGPSGCGKSTLLRLLFRFYDPQKGAIYIDGQDIRSVTLESLRRAIGVV
Atm1_Hom_sap     AIVGGSGSGKSTIVRLLFRFYEPQKGSIYLAGQNIQDVSLESLRRAVGVV
Atm1_Tri_vag     AFVGHSGCGKSTIVQLLLRFYDVNSGEVLIDGRNIKEYSPSFIHRNIGVV
Atm1_Cry_par     GFVGSSGSGKTTLAKLIYRIFEPNSGKIRIFGKEIEDYEINEYRNCFAVL

Atm1_Tra_hom1    PQNSYLVSGTVKENIKYGNLVATDEEMIELCRSLNFHESFVRLSSGYETC
Atm1_Tra_hom2    SQEPQLFADTVLYNVAYGS-KAKLRCIIAIAMKMGVHKDILRMR-GYGSL
Atm1_Tra_hom3    PQSPLVFKNTIRFNLGYGN-CATDEGMTKMCKHMNLFDKLVEMEDGLDSL
Atm1_Enc_cun1    PQNSSLFNETVMYNIKYGSPSVSDYAVVELAKRFNIHDSIMRLERGYFTN
Atm1_Enc_int1    PQNSSLFNETVMYNIKYGNPNVSDYTVVELAKRFNIHDSIMRLEKGYFTN
Atm1_Nos_cer1    PQNTFLFNESVKYNIKYGSFGISDEDIFALCKEFGLYDVFMNLENGFDTN
Atm1_Nos_cer2    PQTSFLFNETVYYNLTYGKEIFDKEEVIKISKKLCVHDSINNLEDEYNTH
Atm1_Nos_cer3    TQNTFLFDNTVTYNIFYGTKNVTEKEVLELAKKIGVLESIQEFKDGFSTS
Atm1_Sac_cer     PQDTPLFNDTIWENVKFGRIDATDEEVITVVEKAQLAPLIKKLPQGFDTI
Atm1_Neu_cra     PQDTPLFNDTVEHNIRYGNLSATPEQVIEAAKAAHIHEKIISWRDGYNTK
Atm1_Hom_sap     PQDAVLFHNTIYYNLLYGNISASPEEVYAVAKLAGLHDAILRMPHGYDTQ
Atm1_Tri_vag     QQDSALFTLSVRDNILYGKTDSTNEDVENAAKVAFAHNFIIKLPHQYDSM
Atm1_Cry_par     PQEVLLLNMSIIDNLKIANNNATLDEIKSACKLAGVHENILKMKNGYETI
```

```
Atm1_Tra_hom1    IGENNSVLSGGEKQKVAIARAML---------------------------
Atm1_Tra_hom2    LTENARNLSGGERQKITLLRNVVYGICACGDGVDCAHCEGCRDGSVQEWS
Atm1_Tra_hom3    ILSGGENFSGGEKKRLCVARAAL---------------------------
Atm1_Enc_cun1    VGECGRHISGGERQKIVILRALL---------------------------
Atm1_Enc_int1    VGEAGRHISGGERQKIIILRALL---------------------------
Atm1_Nos_cer1    VGERGRLLSGGEKQKILLMRTML---------------------------
Atm1_Nos_cer2    VGDKGHKLSGGERQKVILLRSAL---------------------------
Atm1_Nos_cer3    VGERGRFLSGGERQKIMLMRALL---------------------------
Atm1_Sac_cer     VGERGLMISGGEKQRLAIARVLL---------------------------
Atm1_Neu_cra     VGERGLMISGGEKQRLAVSRLIL---------------------------
Atm1_Hom_sap     VGERGLKLSGGEKQRVAIARAIL---------------------------
Atm1_Tri_vag     VGEKGTTLSGGQRQRIAIARAVL---------------------------
Atm1_Cry_par     VGERGCSLSGGEKQRLGFARMLI---------------------------

Atm1_Tra_hom1    --------------------------------------------------
Atm1_Tra_hom2    DEEERNVKISVADRESITQFNDDHQAFNSDFVPYEVTMQDDTYQNATEDR
Atm1_Tra_hom3    --------------------------------------------------
Atm1_Enc_cun1    --------------------------------------------------
Atm1_Enc_int1    --------------------------------------------------
Atm1_Nos_cer1    --------------------------------------------------
Atm1_Nos_cer2    --------------------------------------------------
Atm1_Nos_cer3    --------------------------------------------------
Atm1_Sac_cer     --------------------------------------------------
Atm1_Neu_cra     --------------------------------------------------
Atm1_Hom_sap     --------------------------------------------------
Atm1_Tri_vag     --------------------------------------------------
Atm1_Cry_par     --------------------------------------------------

Atm1_Tra_hom1    ---------------------------KNAE--IYLFDEPTANLD-----
Atm1_Tra_hom2    GSTTNYNMMYDVDEISSKNEETFYPSAKNSPSILMLFDEATSAMD-----
Atm1_Tra_hom3    ---------------------------KRCE--IYWFDEPTAGLD-----
Atm1_Enc_cun1    ---------------------------KRSE--ILVMDEPTSNLD-----
Atm1_Enc_int1    ---------------------------RRPE--ILAMDEPTSNLD-----
Atm1_Nos_cer1    ---------------------------RNKE--IMALDEPTAALD-----
Atm1_Nos_cer2    ---------------------------KNSP--IIILDEPTAALD-----
Atm1_Nos_cer3    ---------------------------KNSE--IVLLDEPTSALD-----
Atm1_Sac_cer     ---------------------------KNAR--IMFFDEATSALD-----
Atm1_Neu_cra     ---------------------------KDPP--LLFFDEATSALD-----
Atm1_Hom_sap     ---------------------------KDPP--VILYDEATSSLD-----
Atm1_Tri_vag     ---------------------------KNPS--LLITDEATAALD-----
Atm1_Cry_par     ---------------------------KKSP--IWILDEPTSALDLINHN

Atm1_Tra_hom1    -------------------------------------------KKSEETF
Atm1_Tra_hom2    -------------------------------------------KSSEYDV
Atm1_Tra_hom3    -------------------------------------------STNMHAV
Atm1_Enc_cun1    -------------------------------------------KEAEIDI
Atm1_Enc_int1    -------------------------------------------KEAEIDI
Atm1_Nos_cer1    -------------------------------------------KESEKKI
Atm1_Nos_cer2    -------------------------------------------KKAEYEI
Atm1_Nos_cer3    -------------------------------------------KKSELET
Atm1_Sac_cer     -------------------------------------------THTEQAL
Atm1_Neu_cra     -------------------------------------------THTEQAL
Atm1_Hom_sap     -------------------------------------------SITEETI
Atm1_Tri_vag     -------------------------------------------SVSEKKV
Atm1_Cry_par     FMVKMLSFLHEYSLSTSNSVNLNDKYDLGSKYKDIFSLIPYVKDKKEIQS
```

```
Atm1_Tra_hom1   FKRLAEDT-------QNKTVIVILHNLELLDYFDRIMFLQKDRIQEIKRE
Atm1_Tra_hom2   IERLFDVC-------SECTVLMVIHNLTILHLFDTIIYVDKE--LEIGSF
Atm1_Tra_hom3   VDFITR---------MKGTVIAIMHTDEYDRCFDQVIYLERM--------
Atm1_Enc_cun1   IRNIIDSE-------GSVTVIAIVHNLDLLPFFNKVCFVDKGSAKMISQT
Atm1_Enc_int1   IKNIIDFE-------SSVTVMAIVHNLDLLPLFNKVCFVDRGSVRMIDQT
Atm1_Nos_cer1   LEIILQQ--------EDRTVLMIIHNLELINKFDKIIYIDENNIEVYKND
Atm1_Nos_cer2   LKSLISNN-------LEKTIIIVLHNLDLLHLFDKVLSIKNKTVSL----
Atm1_Nos_cer3   MTYIFNEF-------KYHTFVIIVHNLELLALFDKILFVNGNEVTMIEDI
Atm1_Sac_cer    LRTIRDNF-----TSGSRTSVYIAHRLRTIADADKIIVLDNGRVREEGKH
Atm1_Neu_cra    MENINAILKGLGQKGEKKTSLFVAHRLRTIYDSDLIIVLKEGRVAEQGTH
Atm1_Hom_sap    LGAMKDVV-------KHRTSIFIAHRLSTVVDADEIIVLDQGKVAERGTH
Atm1_Tri_vag    EKALRSVM-------SSRTSIIIAHRLGTIRCASHIFVLDDGEVVEEGSH
Atm1_Cry_par    IKKLIDDIV-----KLPLTIIVIAHRLSSVRNFDLIAYLEEGNVKEVGNH

Atm1_Tra_hom1   EALKLMNSTKNEKSTKNAQEM-----------------------------
Atm1_Tra_hom2   ---DRLMQKKG-NFYLFYERM-----------------------------
Atm1_Tra_hom3   --------------------------------------------------
Atm1_Enc_cun1   NGAARSIAERL-RDC-----------------------------------
Atm1_Enc_int1   GTSSESAAEGL-RSY-----------------------------------
Atm1_Nos_cer1   IEGQKKFSTNL-QYFLS---------------------------------
Atm1_Nos_cer2   ----HKIEENI---------------------------------------
Atm1_Nos_cer3   ---NKKLEENSKYFA-----------------------------------
Atm1_Sac_cer    ---LELLAMPGSLYRELWTIQEDLD-----------------------HL
Atm1_Neu_cra    ---RELMERNG-VYAQLWRAQEMLMTEEGEVS--------------KKGE
Atm1_Hom_sap    ---HGLLANPHSIYSEMWHTQSSRVQNHDNPKWEAKKENISKEEERK-KL
Atm1_Tri_vag    ---DELISRRG-VYYELVKI-------------------------------
Atm1_Cry_par    ---DQLIENKM-QYYQLWNKQ------------------------------

Atm1_Tra_hom1   ---CDT------SEEQ
Atm1_Tra_hom2   ----RK------ERRD
Atm1_Tra_hom3   ----------------
Atm1_Enc_cun1   ----GM------LGVK
Atm1_Enc_int1   ----EI------PGVK
Atm1_Nos_cer1   -----Y------KLGK
Atm1_Nos_cer2   ----IS------TLNL
Atm1_Nos_cer3   ----DI------LNNE
Atm1_Sac_cer    ENELKD------QQEL
Atm1_Neu_cra    KEEVGE------KKEA
Atm1_Hom_sap    QEEIVNSVKGCGNCSC
Atm1_Tri_vag    ------------QLDA
Atm1_Cry_par    ---HID------SILK
```

Abbreviations: Tra_hom, *Trachipleistophora hominis*; Enc_cun, *Encephalitozoon cuniculi;* Enc_int, *Encephalitozoon intestinalis*; Nos_cer, *Nosema ceranae*; Sac_cer, *Saccharomyces cerevisiae*; Neu_cra, *Neurospora crassa*; Hom_sap, *Homo sapiens;* Tri_vag, *Trichomonas vaginalis*; Cry_par, *Cryptosporidium parvum.*

Atm1 proteins are half size transporters that belong to subfamily B of the ABC transporter family[17,18]. The structure of these proteins includes an N-terminal domain with six transmembrane domains (TMD1-6) followed by a nucleotide-binding fold (NBF). The predicted TMD are labelled in brown. These were predicted with the TOPCONS web server: http://topcons.cbr.su.se [19].

The motif for the ATP binding box A (Walker A motif; G-X-X-G-X-G-K-S/T-X-X-X-X-X-I/V) is labelled in red. Note that in some cases the I/V residue is replaced by another hydrophobic amino acid. The mutation K475M within this motif resulted in the complete loss of *Saccharomyces cerevisiae* Atm1 function[20]. The conserved basic amino acid that precedes the Walker A motif is labelled in blue. The ATP binding box B (Walker B motif; Φ-Φ-Φ-Φ-D, where Φ represents hydrophobic residue) is labelled in pink. The Q-loop of the conserved ATP-binding motif is labelled in green. The conserved glutamate that acts as a catalytic base is labelled in dark green[21].

The ABC signature motif (LSGG), also called the C-loop, which is present in the nucleotide-binding fold, is labelled in grey[22]. The D-loop (SALD), which interacts with the Walker A motif is labelled in teal[23]. The conserved histidine that is proposed to be involved in the catalytic reaction is labelled in light grey[24]. The residues identified in *Saccharomyces cerevisiae* that interact with glutathione are labelled in yellow. The star (★) corresponds to the human Atm1 E433K mutation found in patients with X-linked sideroblastic anemia and cerebellar ataxia (XLSA/A)[25].

e. P-loop NTPase Cfd1 and Nbp35

```
Cfd1_Tra_hom     M-------------------------------------------------
Cfd1_Enc_cun     M-------------------------------------------------
Cfd1_Enc_int     M-------------------------------------------------
Cfd1_Nos_cer     M-------------------------------------------------
Cfd1_Ent_bie     MP------------------------------------------------
Cfd1_Sac_cer     MEE-----------------------------------------------
Cfd1_Neu_cra     MS------------------------------------------------
Cfd1_Hom_sap     MEA-----------------------------------------------
Cfd1_Tri_vag     M-------------------------------------------------
Cfd1_Ent_his     MTE-----------------------------------------------
Nbp35_Tra_hom    MSK------------------------------------------CPGIH
Nbp35_Enc_cun    MGES-----------------------------------------CPGVS
Nbp35_Enc_int    MGES-----------------------------------------CPGVS
Nbp35_Nos_cer    MQNN------------------------------------------YKCP
Nbp35_Ent_bie    MNN------------------------------------------CSNVD
Nbp35_Sac_cer    MTEIL-----PHVNDEVL--------------PAEYELNQPEPEHCPGPE
Nbp35_Neu_cra    MAPSLEAEPESVASVLAN--------------PQKPQLVAPEPEHCPGPE
Nbp35_Hom_sap    MEEV--------------------------------------PHDCPGAD
Nbp35_Tri_vag    MSCS----------------------------------------------
Nbp35_Gia_int    MDCIAYPPRSHRENKMPCCGNSGNGPCACHSGANGVESDLPKSGNKPVSD
Nbp35_Ent_his    C-------------------------------------------------
Nbp35_Cry_par    IQI----------------------------------------GNCVGVD

Cfd1_Tra_hom     --------------------------------------------------
Cfd1_Enc_cun     --------------------------------------------------
Cfd1_Enc_int     --------------------------------------------------
Cfd1_Nos_cer     -----------------------------------------------ERI
Cfd1_Ent_bie     ----------------------------------------------KKNE
Cfd1_Sac_cer     -------------------------------------QEIGVPAASLAGI
Cfd1_Neu_cra     ----------------------------------------------LAKV
Cfd1_Hom_sap     ----------------------------------------AAEPGNLAGV
Cfd1_Tri_vag     ------------------------------------------------ST
Cfd1_Ent_his     -------------------------------------LNSDRNFVGVDHV
Nbp35_Tra_hom    SE--------RAGQAEACASCPNSTYCQQTTN---SSTITKSRIARNTAG
Nbp35_Enc_cun    SK--------DAGKAEECKGCPNVGYCSQPVQ---QDPDIKAIQENLSGV
Nbp35_Enc_int    SK--------DAGKAKECKGCPNASYCSQPAQ---PDPDIKIIQENLRGV
Nbp35_Nos_cer    TS--------NFGKSEQCNECPNQSICGT-VK---PNDSLPLISANVSHF
Nbp35_Ent_bie    KK--------------QCENCPNRDNCYG-N--C-EDEDINLIKNKLQCF
Nbp35_Sac_cer    SD--------MAGKSDACGGCANKEICESLP--KGPDPDIPLITDNLSGI
Nbp35_Neu_cra    SQ--------QAGTADSCAGCPNQAICATAP--KGPDPDIPLITARLSGV
Nbp35_Hom_sap    SA--------QAGRGASCQGCPNQRLCASGA-GATPDTAIEEIKEKMKTV
Nbp35_Tri_vag    ---------------GNCGSCSHAGTCSSHGTPEALQGALEECKTVLENV
Nbp35_Gia_int    APTPEQISLKGECAPDKCSGCPARGACSSRGA---DSSTSIAIAERIQHV
Nbp35_Ent_his    ---------------------------GGG--NNGPDRELEEIIEKLKGI
Nbp35_Cry_par    SP--------DAGIADSCAGCPNALICAS-G--Q-AKKKPTENIENLSKI
```

```
Cfd1_Tra_hom      -LFISVVSGKGGVGKSTIAALIAAKLSKQ--APTLLLDFDICGPSIGNIF
Cfd1_Enc_cun      -SRIAVMSGKGGVGKSSVSIMLSTVLSEK--GRTLLLDFDLCGPSIASGF
Cfd1_Enc_int      -VMVAVMSGKGGVGKSSISIMLSTAMSER--GKTLLLDFDLCGPSVASGL
Cfd1_Nos_cer      PQKIAVMSGKGGVGKSSISILLSTILSEK--HKCLLLDFDLCGPSCFSSL
Cfd1_Ent_bie      PKIYCILSGKGGVGKSAVAAFLALQLKKN--LKVLFIDFDICGPSAAIYF
Cfd1_Sac_cer      KHIILILSGKGGVGKSSVTTQTALTLCSMG-FKVGVLDIDLTGPSLPRMF
Cfd1_Neu_cra      KHIVLVLSGKGGVGKSSVTTQLALSLSLAG-HSVGVLDVDLTGPSIPRMF
Cfd1_Hom_sap      RHIILVLSGKGGVGKSTISTELALALRHAG-KKVGILDVDLCGPSIPRML
Cfd1_Tri_vag      QNFILVMSGKGGVGKSTTAANIARAYAAKY-GKVGLLDLDLTGPSIPTLF
Cfd1_Ent_his      KNVILVLSGKGGVGKSTIATVLARSFALAG-KKTGILDIDLCGPSIPKMM
Nbp35_Tra_hom     KKIIAVMSGKGGVGKSTMCMQIAHALK-----KCCVLDFDVSGPSIAKMS
Nbp35_Enc_cun     KAVIAVMSGKGGVGKSTVTRNIAELMSSRG-IATCILDLDLSGPSIPRLT
Nbp35_Enc_int     KTIVAIMSGKGGVGKSTVVRNIAESVSSRG-ITTCILDLDLSGPSIPRLT
Nbp35_Nos_cer     KLIFSIFSGKGGVGKSTITRNIAEFLSLKN-YKVLLLDLDLSGPSIPKMT
Nbp35_Ent_bie     KLILCIMCGKGGVGKSLLSVILAQYFSEK--FKTILIDLDLAGSSIPRLT
Nbp35_Sac_cer     EHKILVLSGKGGVGKSTFAAMLSWALSADEDLQVGAMDLDICGPSLPHML
Nbp35_Neu_cra     KHKILILSGKGGVGKSTFTSLLAHAFATNAEQTVGVMDTDICGPSIPKML
Nbp35_Hom_sap     KHKILVLSGKGGVGKSTFSAHLAHGLAEDENTQIALLDIDICGPSIPKIM
Nbp35_Tri_vag     THKILILSGKGGVGKSTLTYILTKYLAK-T-KKVGVLDLDLCGPSIPILF
Nbp35_Gia_int     GRIILVLSGKGGVGKSTLATQLAFFLADTMGKYVGLLDLDICGPSIPTMT
Nbp35_Ent_his     KHKYVILSGKGGVGKSTFATQFSWVLSED--KQVGLCDYDICGPSIPQMF
Nbp35_Cry_par     KNIILVLSGKGGVGKSTISSQISWCLSSKK-FNVGLLDIDICGPSAPKMM
                                       Walker A motif

Cfd1_Tra_hom      PASGK-VVKTRT-GLKPLQVNN-------SS-LYILSMSSLI-KNDSSVI
Cfd1_Enc_cun      GAKEN-VYKGEK-GLVPIRVSK--------N-LYILSMALLM-KDSDSVI
Cfd1_Enc_int      GIKEK-IYKGEK-GLIPAKASE--------N-LYILSMALLM-KESDSVI
Cfd1_Nos_cer      NGKGE-VKKAKK-GLTPIQITN--------N-LYVLSMGSMI-KPDDAVI
Cfd1_Ent_bie      NVTGK-ITKHKN-GFKPLTLDS--------N-LDILSFGNIL-GENDVVI
Cfd1_Sac_cer      GLENESIYQGPE-GWQPVKVETN----STGS-LSVISLGFLLGDRGNSVI
Cfd1_Neu_cra      GIEDAKVTQAPG-GWLPITVHEADPSAGVGS-LRVMSLGFLLPKRGDAVV
Cfd1_Hom_sap      GAQGRAVHQCDR-GWAPVFLDR------EQS-ISLMSVGFLLEKPDEAVV
Cfd1_Tri_vag      GIQDKEIKSRNG-KMVP-QVVD--------G-VQIISLGLMLSDPHDAVI
Cfd1_Ent_his      GLDNQGVYQGEHGGILPAKSKI-----GDTF-IDTLSVGFMLSSPDSPVI
Nbp35_Tra_hom     GTENAIITNVQD-TFVPVHVHG--------TCIGVVSAYHVNEWHSVEQL
Nbp35_Enc_cun     GTDGQLMCETNG-RLQPVEVHG--------L-LKAVSAGYLQDPCEEGVV
Nbp35_Enc_int     GTDGMSMCETSG-IIQPIEVNK--------F-LKVVSVGYLQD-CGEGIM
Nbp35_Nos_cer     HTEGEIIIESNK-RFYPVKLSE--------N-LGCISVGYFADSQPSQNL
Nbp35_Ent_bie     NTTDYFITNVEN-QFNPIKVNE----------LSVVSMGHIHNNISD--I
Nbp35_Sac_cer     GCIKETVHESNS-GWTPVYVTD--------N-LATMSIQYMLPEDDSAII
Nbp35_Neu_cra     GVEGETIHVSST-GWSPAWAMD--------N-LAVMSIQFMLPNRDDAII
Nbp35_Hom_sap     GLEGEQVHQSGS-GWSPVYVED--------N-LGVMSVGFLLSSPDDAVI
Nbp35_Tri_vag     NCDVEPLLDTTF-GFQPYHAAK--------N-INVVSIQFFLPDFDSPLV
Nbp35_Gia_int     FTKTEQVQNLPM-GWEPVSVSH--------T-LQALSVGHLVTQEDAPVI
Nbp35_Ent_his     GQIGVNVTSGMT-GLQPIYVTE--------N-LCTMSIGYLV-ATETAVV
Nbp35_Cry_par     GVQGNDVHISAN-GWSPVYVND--------N-LSVMSTAFLLPQSDDAVI
```

```
Cfd1_Tra_hom     WRAPRKLQLYEMFYNSAYENILQNTTESDPDSYDILDKHNNIGNKTAQKN
Cfd1_Enc_cun     WRGPKKMSVLSMFYESID--------------------------------
Cfd1_Enc_int     WRGPKKMSVLSMFYESAD--------------------------------
Cfd1_Nos_cer     WRGPKKLSLLNLFYDSID--------------------------------
Cfd1_Ent_bie     WRGAKKQIFLELMFNTSLFK------------------------------
Cfd1_Sac_cer     WRGPKKTSMIKQFISDVAWG------------------------------
Cfd1_Neu_cra     WRGPKKTAMVRQFLSDVFWD------------------------------
Cfd1_Hom_sap     WRGPKKNALIKQFVSDVAWG------------------------------
Cfd1_Tri_vag     WRGPKKSAMINQFFQLIEW-------------------------------
Cfd1_Ent_his     WRGPKKGAAIEQFLNDVEWG------------------------------
Nbp35_Tra_hom    YQPSFISSFLINVLSNCNFD------------------------------
Nbp35_Enc_cun    FSSTLKTSAMKKLLKWCSYE------------------------------
Nbp35_Enc_int    FSSSFKTGIIKKFLAQCNYE------------------------------
Nbp35_Nos_cer    FSSTYKTNTIRNILINGDIA------------------------------
Nbp35_Ent_bie    YTSEIKRYFIKNILKNCTMD------------------------------
Nbp35_Sac_cer    WRGSKKNLLIKKFLKDVDWD------------------------------
Nbp35_Neu_cra    WRGPKKNGLIKQFLKDVEWG------------------------------
Nbp35_Hom_sap    WRGPKKNGMIKQFLRDVDWG------------------------------
Nbp35_Tri_vag    ARGPKKNALVLQLINQIDWS------------------------------
Nbp35_Gia_int    LRGPKKHGMVKQMLTETNWE------------------------------
Nbp35_Ent_his    WKGPKKNSLIRQFIHDVDWG------------------------------
Nbp35_Cry_par    WRGPKKNGLIKQFLSDVVWG------------------------------

Cfd1_Tra_hom     VTVDESCQLGYKYVVIDTPPGITIVHSFIKE----------------KN-
Cfd1_Enc_cun     ---------GFDNVVFDMPPGISEEHGFLIG----------------KD-
Cfd1_Enc_int     ---------GFDNVVIDMPPGISEEHGFLVG----------------KD-
Cfd1_Nos_cer     ---------DFDFVIIDTPPGVSEEHGFLID----------------KN-
Cfd1_Ent_bie     ---DEDGNFIYDAILIDTPPGISEEHGFLVG---------------KKN-
Cfd1_Sac_cer     ---------ELDYLLIDTPPGTSDEHISIAE-------EL----RYSK-P
Cfd1_Neu_cra     ---------ETDYLLIDTPPGTSDEHISLAE-------NLLQKARPGQ-L
Cfd1_Hom_sap     ---------ELDYLVVDTPPGTSDEHMATIE-------AL----RPYQ-P
Cfd1_Tri_vag     ---------DCNTVIVDLPPGTSDEHLSTFD-------VLN---RN-NFS
Cfd1_Ent_his     ---------DKDVLVVDTPPGTSDEHITIMD-------FF----RKRNQE
Nbp35_Tra_hom    ---------TYTHVVIDTPPGITDEHLIISN-------YV---------D
Nbp35_Enc_cun    ---------GTDVLLLDTPPNVTDEHLGMVN-------FI----R----P
Nbp35_Enc_int    ---------GVDVLLLDTPPNVTDEHLGMVN-------FI----K----P
Nbp35_Nos_cer    ---------DYEILIIDTPPNVTDEHLGIVN-------YL----K----L
Nbp35_Ent_bie    ---------NKEILILDTPPNITEEHFAIYN-------YI----C----N
Nbp35_Sac_cer    ---------KLDYLVIDTPPGTSDEHISINK-------YM----RESG-I
Nbp35_Neu_cra    ---------DLDFLLVDTPPGTSDEHLSVNT-------YL----KKSG-I
Nbp35_Hom_sap    ---------EVDYLIVDTPPGTSDEHLSVVR-------YL----ATAH-I
Nbp35_Tri_vag    ---------DQDFLLVDTPPGTSDEHLSVVS-------FM----RDSE-I
Nbp35_Gia_int    ---LDPRFPKSNIIIVDTPPGTSDEHLSIIDMYQNAIRYMQSNAFPNVPV
Nbp35_Ent_his    ---------ELDYLIIDTPPGTSDEHLTIVS-------IL----NKCN-V
Nbp35_Cry_par    ---------ELDFLIIDTPPGTSDEHLSIVS-------YL----NGSN-V
                          Walker B motif
```

```
Cfd1_Tra_hom     TKILLVTTSQNVAISDSINTINFFGK----ISGIIENMSGLKCPNCKKIT
Cfd1_Enc_cun     VGALIITTPQNVSLGDSSKAIDFCASNGIRILGLVENMSGYCCECCGSSV
Cfd1_Enc_int     ISVLIATTPQNISLGDSSRAIDFCISNGIQILGLVENMSGYCCESCGNPT
Cfd1_Nos_cer     IYSLIVTTSQNVALSDTVKAIDFCKINNIKILGIIENLSGYKCNCCGHIT
Cfd1_Ent_bie     VHSLIVTTGQNLALNCCQSTIEFCLYHNLNIIGVIQNMSYYVCECCHEKI
Cfd1_Sac_cer     DGGIVVTTPQSVATADVKKEINFCKKVDLKILGIIENMSGFVCPHCAECT
Cfd1_Neu_cra     AGAVVVTTPQAVATADVRKELNFCTKTNIRVLGVVENMCGFVCPNCSECT
Cfd1_Hom_sap     LGALVVTTPQAVSVGDVRRELTFCRKTGLRVMGIVENMSGFTCPHCTECT
Cfd1_Tri_vag     YSVIIVTTPNVLAVADVRKGINLCLKVNAKIIGIIENFCGVVCPCCNKVS
Cfd1_Ent_his     TKAVIVTTPQLVSTNDVEKEIDFCNECQIPIIGLVENMSGYLCPHCSTVT
Nbp35_Tra_hom    AVCVLVSTPGVLAVNDLVRQIDFCERAGVKVLGVVENMREFVCE-CGCVV
Nbp35_Enc_cun    RFGIVVTTPQKFSLQDVARQVDFCRKARIEVLGIIENMKRFTCQKCGHSK
Nbp35_Enc_int    KFAIVVTTPQKFSLQDVIRQIDFCRKAKISVLGVIENMKRFVCPRCSHQK
Nbp35_Nos_cer    NFAIVVTTPQLISFQDVIRQYTFCYKNNIKILGIIENMKGFRCEKCDSLQ
Nbp35_Ent_bie    AKAILISTPHVLCTTELNRQFIFCQKANIDIVGIVSNMDGIRCSKCNHIN
Nbp35_Sac_cer    DGALVVTTPQEVALLDVRKEIDFCKKAGINILGLVENMSGFVCPNCKGES
Nbp35_Neu_cra    DGAVMVTTPQEVSLLDVRKEIDFCRKAGIKVLGLVENMSGFVCPKCTHES
Nbp35_Hom_sap    DGAVIITTPQEVSLQDVRKEINFCRKVKLPIIGVVENMSGFICPKCKKES
Nbp35_Tri_vag    DGAVIVTTPDEVSISDVRREIEFCQKAGVKILGVVENMSQYKCPMCGKTS
Nbp35_Gia_int    LEAVVVSTPQEVALADVRKEINFCKQLNLHIKGVIENMSGFVCPFCETET
Nbp35_Ent_his    DGAIIITTPQDVSLIDVRKEINFCKKIGLPIIGVVENMSGFICPCCHKES
Nbp35_Cry_par    NGALIVTTPQEIALQDVRKEINFCKKVGLNILGVVENM-GMIFKNAEHDS

Cfd1_Tra_hom     NIY--SRNGGSQLAEEFNIPFYGTLEIDQNISQFIENGTLY---------
Cfd1_Enc_cun     NIF--GSKGGERLAEETGIPFVCRLPIDSLLCEALDEGRFV---------
Cfd1_Enc_int     NIF--GARGGERLAMEMGVRFICELKIDPLLCEALDEGKFL---------
Cfd1_Nos_cer     NIF--ASKGGQQLSQHYLINFIEKLPIEPLFGELLDTKEFI---------
Cfd1_Ent_bie     YLY--GKNGGKLLAEEYGIEYLGEIPMESQMLNAIEQGQFP---------
Cfd1_Sac_cer     NIF--SSGGGKRLSEQFSVPYLGNVPIDPKFVEMIENQVSSKK-------
Cfd1_Neu_cra     NIF--MSGGGEVMANDFGVRFLGRVPIDPQFLVLIETGKRPTYPAGTTVD
Cfd1_Hom_sap     SVF--SRGGGEELAQLAGVPFLGSVPLDPALMRTLEEGHDF---------
Cfd1_Tri_vag     PLL--GDKAAEIMSEELQLDILAKIPFLPQAASAADKGEKS---------
Cfd1_Ent_his     NIF--SSNGGKELADKYQLKFVGAIPIEPKICLAGETGLN----------
Nbp35_Tra_hom    PM---GTVDVREVCLRRGVRYLGGLQCVKAVGMFADGGMVY---------
Nbp35_Enc_cun    SIF--RSVGVESYCMSNGIAYLGSIDLKQDIAKRSDSGDTI---------
Nbp35_Enc_int    NVF--VNTEVESYSKSNGIPYLGSIDLRQDIAKASDIGRPT---------
Nbp35_Nos_cer    DIF--YNSDIEQKCKENNLNYIGSLPLNIEYGKSGDNGILI---------
Nbp35_Ent_bie    QLF--SKDIILKFCQNKYIQFLGEIEFNSQIVKNIDKGEVI---------
Nbp35_Sac_cer    QIFKATTGGGEALCKELGIKFLGSVPLDPRIGKSCDMGESF---------
Nbp35_Neu_cra    EIFKATTGGGRKLAEEMGIAFLGSVPLDPRIGMACDYGESF---------
Nbp35_Hom_sap    QIFPPTTGGAELMCQDLEVPLLGRVPLDPLIGKNCDKGQSF---------
Nbp35_Tri_vag    SIYGHEFGGAEELCKQENLDLLGRIPIDPYIVAGQFEPQK----------
Nbp35_Gia_int    PVIEATTGGVKKMCEDMHVPYIGSMPLDPQLMKAGEDGVAW---------
Nbp35_Ent_his    TIFPPTHGGAKQMCEEMGVKFLGKIPLDPIIAHSCDIGAPY---------
Nbp35_Cry_par    SV--------KDMCDNMEVEYLNKIPWDKELLYVCDLGLSI---------
```

```
Cfd1_Tra_hom     -------------------------ENINSLGCNNVLDTVVR---K----
Cfd1_Enc_cun     -------------------------ERCGSIEAYMKFRKAVL---G----
Cfd1_Enc_int     -------------------------EKCGSIESYIRLRRSVL---E----
Cfd1_Nos_cer     -------------------------LKYQELKTYKILKKWINR--E----
Cfd1_Ent_bie     -------------------------SYCEATGIFNDIQRKIS---H----
Cfd1_Sac_cer     ----------------------TLVEMYRESSLCPIFEEIMKKLRKQDTT
Cfd1_Neu_cra     GKDISTPAGASTSEEEEVKDGSRLVHKYKDCSLAPIFSKITADVISA---
Cfd1_Hom_sap     ------------------------IQEFPGSPAFAALTSIAQKILDATP-
Cfd1_Tri_vag     -------------------------D-----VILSFFNEVIDKIFPQQ--
Cfd1_Ent_his     --------------------------PFADEPSANALKPITDFVADLA--
Nbp35_Tra_hom    -------------------------ED-------ALFAGVVRNITDE---
Nbp35_Enc_cun    -------------------------EE-------EVLGKIVDAIMVVC--
Nbp35_Enc_int    -------------------------RE-------EIFDRMADAVLSI---
Nbp35_Nos_cer    -------------------------DD-------QIFSKTIDLIINE---
Nbp35_Ent_bie    -------------------------HIPQLKSIYSKLLSIIMEFVPFNK-
Nbp35_Sac_cer    ------------------------LDNYPDSPASSAVLNVVEALRDA---
Nbp35_Neu_cra    ------------------------FDSFPDSPACRALKGVVKGLATEMGL
Nbp35_Hom_sap    ------------------------FIDAPDSPATLAYRSIIQRIQEFCNL
Nbp35_Tri_vag    -----------------------DLPEAINDAASVICEKIQQKLS-----
Nbp35_Gia_int    ----------------------STICDIDTSPGYDAFANICGKII-----
Nbp35_Ent_his    ------------------------FLEHPDSEATKNFKRIYKEIIT----
Nbp35_Cry_par    ------------------------CEKFPQSPSSIGIKKLVDIIIYQ---

Cfd1_Tra_hom     ------------LVGS
Cfd1_Enc_cun     ------------LADI
Cfd1_Enc_int     ------------ITNI
Cfd1_Nos_cer     ------------NWTF
Cfd1_Ent_bie     ------------L--L
Cfd1_Sac_cer     TPVVDKHEQPQIESPK
Cfd1_Neu_cra     -------------VQQ
Cfd1_Hom_sap     ------------ACLP
Cfd1_Tri_vag     ------------KAAQ
Cfd1_Ent_his     ------------KTFA
Nbp35_Tra_hom    -------------LEK
Nbp35_Enc_cun    ------------SSKA
Nbp35_Enc_int    -------------HES
Nbp35_Nos_cer    --------------IK
Nbp35_Ent_bie    -----------KNDFN
Nbp35_Sac_cer    ------------VGDV
Nbp35_Neu_cra    DPEV----VMPEEDDA
Nbp35_Hom_sap    HQS-----KEENLISS
Nbp35_Tri_vag    ---------------A
Nbp35_Gia_int    ---------------E
Nbp35_Ent_his    --------------NL
Nbp35_Cry_par    ------------SKIN
```

Abbreviations: Tra_hom, *Trachipleistophora hominis*; Enc_cun, *Encephalitozoon cuniculi*; Enc_int, *Encephalitozoon intestinalis*; Nos_cer, *Nosema ceranae*; Ent_bie, *Enterocytozoon bieneusi*; Sac_cer, *Saccharomyces cerevisiae*; Neu_cra, *Neurospora crassa*; Hom_sap, *Homo sapiens*; Tri_vag, *Trichomonas vaginalis*; Gia_int, *Giardia intestinalis*; Ent_his, *Entamoeba histolytica;* Cry_par, *Cryptosporidium parvum.*

The major structural difference between Cfd1 and Nbp35 is the presence in Nbp35 of an N-terminal Cys-X_{13}-Cys-X_2-Cys-X_5-Cys ferredoxin-like motif (labelled in red) coordinating a [4Fe-4S] cluster[26-28]. The insertion of this cluster depends on electron transfer from the Tah18-Dre2 complex[29]. The genome of *E. histolytica* lacks homologues of Tah18 and Dre2, which is consistent with the loss of the ferredoxin-like motif in *E. histolytica* Nbp35[30]. Cysteine residues in the $CX_{18}CPXCX_2C$ (Cfd1) and $CX_{18}CX_2CX_{38}C$ (Nbp35) motifs that are conserved at the C termini of yeast Cfd1 and Nbp35 homologues are labeled in yellow and dark green respectively. The two central cysteine residues of both motifs, which are conserved in microsporidian sequences, are essential for yeast viability and for maturation of cytosolic and nuclear Fe/S proteins. These motifs also have a critical role in Cfd1-Nbp35 complex formation[26,28,31]. Cfd1 and Nbp35 are classified as Mrp-like proteins that belong to the Mrp/Nbp35 subfamily of P loop NTPases. Nucleotide binding and/or hydrolysis is apparently critical for loading an Fe/S cluster onto the Cfd1-Nbp35 complex. The consensus Walker A motif of the Mrp family and the ENMS motif are labeled in pink and light blue respectively. The Asn in the ENMS motif is predicted to form a contact with the adenine from ATP[32]. The Walker B motif with a conserved Gly residue (typically, within the signature hhhhDxxG, where h is a hydrophobic residue) is labeled in green.

f. WD40 protein Cia1

```
Cia1_Tra_hom     M-----K--------------ITERGSAQFDEKLL--SLAVTKDSVLTGG
Cia1_Enc_cun     M--------------------KYRITSKKLGEKIL--AVHA-GKSIYTGG
Cia1_Enc_int     M--------------------KYKITSKRLDEKIL--AVHV-NGAVYTGG
Cia1_Sac_cer     MASI--NLIKS-----------LKL----YKEKIW--SFDFSQGILATGS
Cia1_Neu_cra     MATETTTATPTAPSATTAEITPLPAFSPDLYQRAWASIPHPSLPLIATCH
Cia1_Hom_sap     MKDSL-VLLGR-----------VPA---HPDSRCWFLAWNPAGTLLASCG
Cia1_Gia_int     MVHDRVSLLNH-----------TAA----HTDRIWRLRASHTGELVASCS
Cia1_Ent_his     M-----QLVDS-----------FEV---------------P---------
Cia1_Cry_par     MGSL--EKLGI-----------IGV----LDSAIWSVASHPKDRIIASCG

Cia1_Tra_hom     TKK-ILHKL-------------------STDLH----TQKILC--QHEK-
Cia1_Enc_cun     TSR-ML-----------------------VNQD----TGEVMC--RCKK-
Cia1_Enc_int     TSR-TL-----------------------VNQD----TGEVMC--RCRK-
Cia1_Sac_cer     TDRKIKLV--------------------SVKY-DDFTLIDVLDETAHKK-
Cia1_Neu_cra     A-HSVTVF--------------------SLST-LS--KHSVLT-GGHTR-
Cia1_Hom_sap     GDRRIRIW--------------------GTEG-DSWICKSVLS-EGHQR-
Cia1_Gia_int     ADGSMAIW--------------------RASKDLSLQLVQRLQ-PGHDNP
Cia1_Ent_his     -------------------------------------LKPLLT--PHNR-
Cia1_Cry_par     S-S-IVVWMDTKLKNHKWYQEIEIVNKCSMAG-NSWVKAYEFGSLEHKR-

Cia1_Tra_hom     -SIRCIA--AKD----------GVIVCGSYDGNATVLY------------
Cia1_Enc_cun     -SVRSIA--SHG----------RYVCCGSYDCTAVLFH------------
Cia1_Enc_int     -SIRSIA--SHG----------RYICCASYDCTAVLFH------------
Cia1_Sac_cer     -AIRSVAWRPHT----------SLLAAGSFDSTVSIWAKEE---------
Cia1_Neu_cra     -SVRSAAWQPPRGKVSGKEAKRLRLVTGSFDTTAGVWTWDQGRREESLER
Cia1_Hom_sap     -TVRKVAWSPCG----------NYLASASFDATTCIWKKN----------
Cia1_Gia_int     VIVRDCAFSAND----------QHLVVAAYDGSMYVYDLID---------
Cia1_Ent_his     -TIRRVKCSKNG-----------LLACCSFDSTVSLWE------------
Cia1_Cry_par     -LIRKIAWSPCG----------GMIISASFDSSISVWEFVS---------

Cia1_Tra_hom     ---------------------EDKILDLI-EGPETEIKGVDLLNGNRRDN
Cia1_Enc_cun     ---------------------DGKVVDVI-EGPDTEVKCVAFSEDG---R
Cia1_Enc_int     ---------------------DGKVVDVI-EGPDTEIKCVGFSEDG---R
Cia1_Sac_cer     ---------------SADRTFEMDLLAII-EGHENEVKGVAWSNDG---Y
Cia1_Neu_cra     EIRLQSGENDQEAEEEEEAEDEWELTLVL-EGHENEVKSVNYSPSG---Q
Cia1_Hom_sap     -------------------QDDFECVTTL-EGHENEVKSVAWAPSG---N
Cia1_Gia_int     ---------------ELTKGDPFQLTAIIANAHEKEIKSVDISKEG----
Cia1_Ent_his     -------------------LNENTIIGTL-EGHESEVKCVDWSFGS---N
Cia1_Cry_par     ------------------RDIGWACICKI-LGPESEVKCVDWSPFN---N

Cia1_Tra_hom     YIALSTRGKTVWVC--------------KLN---DKIEIDSILEDHTQDV
Cia1_Enc_cun     YLAMATRGRSVWVV--------------KID---GEIEIDGVIEDHLHDV
Cia1_Enc_int     YLAMATRGKSVWVV--------------KID---PEIEIDEIIEDHLHDV
Cia1_Sac_cer     YLATCSRDKSVWIW--------------ETDESGEEYECISVLQEHSQDV
Cia1_Neu_cra     YLATCSRDKSVWIWEDVGNPNPSSEEEDEEEEDEDEWETVAVLQEHDGDV
Cia1_Hom_sap     LLATCSRDKSVWVW--------------EVD-EEDEYECVSVLNSHTQDV
Cia1_Gia_int     TVAACSRDRFVSFW--------------RPCSDSPDYDCIGLFNNHTEDI
Cia1_Ent_his     MVATCSRDKSVWLWKSY---------------SGIDYECCSVLTGHSGDV
Cia1_Cry_par     FVAACCRDRAIWFFSLDI-------GENRKLGTLIEYDCIGVVTAHTNDI
```

```
Cia1_Tra_hom     KGVKFF------------------NNLLYTYGYDNTVKVYQRFTMY-----
Cia1_Enc_cun     KGCIFH------------------GGLLFTYGYDNTVKVYDRF-DY-----
Cia1_Enc_int     KGCVFH------------------KGFLFTYGYDNTIKIYERF-DY-----
Cia1_Sac_cer     KHVIWHPS----------------EALLASSSYDDTVRIWKD---Y-----
Cia1_Neu_cra     KAVAWCPDVPGRKGKYAPPRRYGDDVLASASYDNTVRLWRED--G-----
Cia1_Hom_sap     KHVVWHPS----------------QELLASASYDDTVKLYRE---E-----
Cia1_Gia_int     KCVRFSRN----------------GHYLVSASYDNNICLYKRCLETADDLG
Cia1_Ent_his     KTVLFHPS----------------GTILFSGSFDGTIKVWKGE--E-----
Cia1_Cry_par     KKIKWHPT---------------IPMVLLSCSYDNTIIAWAPS--S-QLLG

Cia1_Tra_hom     -----DDSWVLLQSLEAQKE---------------------TVWDVEML-
Cia1_Enc_cun     -----DDSWELVQSID-ERS---------------------TVWCVIFH-
Cia1_Enc_int     -----DDSWELVQSIS-EKN---------------------TVWCVIFH-
Cia1_Sac_cer     -----DDDWECVAVLNGHEG---------------------TVWSSDFDK
Cia1_Neu_cra     -----DGEWVCVAVLEGHEG---------------------TVWGVAWEG
Cia1_Hom_sap     -----EDDWVCCATLEGHES---------------------TVWSLAFDP
Cia1_Gia_int     E--EEIESWIFAGSTKSELDLNSCEMNAPDSEIVASGASCHTVWTAIFMA
Cia1_Ent_his     -----ETEWSELQTIQAYGK---------------------TVWDLKITK
Cia1_Cry_par     HDEVKGLEWVKLYTLNGHSS---------------------TVWDFTYSP

Cia1_Tra_hom     --------TKLFVASNDGCIYVYRK-------------------------
Cia1_Enc_cun     -------NGRMVCTTEEGTVSIYAL-------------------------
Cia1_Enc_int     -------GDKMVCSTEEGTISSYVL-------------------------
Cia1_Sac_cer     ----TEGVFRLCSGSDDSTVRVWKYM-GDDEDD-----------------
Cia1_Neu_cra     RPRENDKFPRLLSWGADEVIRVWSLK-EPEEEEHGE----GAAGGGGNNT
Cia1_Hom_sap     ----S--GQRLASCSDDRTVRIWRQYLPGNEQG--------VACSGS---
Cia1_Gia_int     ----N--NSSILAVDGNGCIRCYNII------------------------
Cia1_Ent_his     ----E--GKFIVAGCANGVIILYEFK------------------------
Cia1_Cry_par     ----N--GEFLLSCSDDSSIVLWNSN-QGNENKFKNLNSVNFALTDTFKM

Cia1_Tra_hom     -------------------EKDWV-FDYCCNISVYPILSLCRIR------
Cia1_Enc_cun     -------------------RSGWT-LEMSRKLSVLPIYSICSVG------
Cia1_Enc_int     -------------------RNGWE-LEACKKLSIFPIYSICSVG------
Cia1_Sac_cer     -------------------QQEWVCEAILPDVHKRQVYNVAWG-FN----
Cia1_Neu_cra     WGFGVPNTMRR------SLKEEWECTAVLPKVHKGDIYSVAWSTET----
Cia1_Hom_sap     -------------------DPSWKCICTLSGFHSRTIYDIAWCQLT----
Cia1_Gia_int     -------------------EGGVKQLGCTILHGRRPVYDISLVEPRHASK
Cia1_Ent_his     -------------------DNLLVELDTINNEKYRDIYSIDIND------
Cia1_Cry_par     IFYNTPNTKRLSKYIQID-QANSFINNYDKELYSYPIYSIEWCNYI----

Cia1_Tra_hom     ---DFLALAVDSKSLVI------------------------VDESLQVKC
Cia1_Enc_cun     ---ENMAYVLNRSSIGI------------------------VDSNLNLVM
Cia1_Enc_int     ---RDMAYVLNRNNIGI------------------------IDSNLNLTT
Cia1_Sac_cer     ---GLIASVGADGVLAVYEE---------------------VDGEWKVFA
Cia1_Neu_cra     ---GLLSSVGSDGVLALYQETANTTEKNEENETNGEAPTTTSAGGWKVLT
Cia1_Hom_sap     ---GALATACGDDAIRVFQEDPNSD---------------PQQPTFSLTA
Cia1_Gia_int     ASSIYIATAGQDGVVCLSIIN--P-----------------ITGTATPIV
Cia1_Ent_his     ---NNVLVGSGDNAIRLFKIN--T-----------------IKKKLELIE
Cia1_Cry_par     ---NCIIVSSADKSLHLFSV---T-----------------DSKRLKHIC
```

```
Cia1_Tra_hom     SLEDAHQK-DINCVKYSDD----------NNMLVTCSDDGCLKVYHVDF-
Cia1_Enc_cun     SIENVHED-SINSIVYDEG----------RNRIVSGGDDGILNTIEL---
Cia1_Enc_int     TIEDIHED-FINGIAYDEG----------RGRIISGGDDGILNVIEM---
Cia1_Sac_cer     KRALCHGVYEINVVKWLEL--------NGKTILATGGDDGIVNFWSLEK-
Cia1_Neu_cra     TVKGAHGPYEINHITWCKRYDAGSERKGEEEMLVTTGDDGVVRPWQVR--
Cia1_Hom_sap     HLHQAHSQ-DVNCVAWNPK---------EPGLLASCSDDGEVAFWKYQRP
Cia1_Gia_int     HITGAHDG-EVNSVCDITQ----AVATDGHVVVCSGGDDGCINIWRIST-
Cia1_Ent_his     EKQDAHTN-DVNCVKWIN-----------KTLSISVGDDNMLKIWKI---
Cia1_Cry_par     ERPNAHNS-EINSVSWLND--------NKRGEFISAGDDGEIALWRFD--

Cia1_Tra_hom     -SE
Cia1_Enc_cun     --L
Cia1_Enc_int     --F
Cia1_Sac_cer     -AA
Cia1_Neu_cra     -IQ
Cia1_Hom_sap     EGL
Cia1_Gia_int     -EE
Cia1_Ent_his     -VN
Cia1_Cry_par     -FE
```

Abbreviations: Tra_hom, *Trachipleistophora hominis*; Enc_cun, *Encephalitozoon cuniculi*; Enc_int, *Encephalitozoon intestinalis*; Sac_cer, *Saccharomyces cerevisiae*; Neu_cra, *Neurospora crassa*; Hom_sap, *Homo sapiens*; Gia_int, *Giardia intestinalis*; Ent_his, *Entamoeba histolytica*; Cry_par, *Crystosporidium parvum*.

Cia1 belongs to the WD40-repeat protein family. WD40 repeats are conserved domains of approximately 44-60 residues that typically contain the GH dipeptide 11-24 residues from its N terminus and the WD dipeptide at the C-terminus. The WD repeat combines a conserved core structure with variable regions that are probably surface-exposed. Most WD proteins contain a cluster of at least 7 or more copies of WD-repeats with as many as 16 but as few as four. A WD protein forms a propeller-like structure with several blades where each blade is composed of a four-stranded anti-parallel β-sheet. Each WD40 sequence repeat forms the first three strands of one blade and the last strand in the next blade. The proposed common function of this protein family is to coordinate the assembly of multi-protein complexes by functioning as a docking site for other proteins. Residues on the top and bottom surface of the propeller are proposed to coordinate interactions with other proteins and/or small ligands[33,34]. The 3D structure of the Cia1 protein from *S. cerevisiae* shows the typical architecture of other WD40-repeat proteins[35,36]. The β propeller structure contains three potential interacting surfaces: The top, the bottom and the circumference[35,36]. The WD40 repeats are shadowed in yellow. The seven WD40 repeats in *S. cerevisae* were obtained from Unipropt KB: http://www.uniprot.org/uniprot/Q05583 and the WD40 repeats from the other organisms were obtained from SMART: http://smart.embl-heidelberg.de/. Note that Tra_hom has four inferred WD repeats, Enc_cun and Enc_int have five WD repeats, Gia_int and Ent_his have six WD repeats. The arginine (R) in green is critical for the function of Cia1 in yeast[36] and is conserved in all of the microsporidian sequences. In **bold** are the invariant residues present also in the microsporidian sequences.

g. Cia2

```
Cia2_Tra_Hom    ---------------------------------MNREPELFE-SAVPECT----------
Cia2_Enc_cun    ---------------------------------MNEFPFVAS-SLEERHP----------
Cia2_Enc_int    ---------------------------------MNKSPFVSS-SLERRYP----------
Cia2_Nos_cer    ---------------------------------MNISPQIKNKNFENRFD----------
Cia2_Ent_bie    ---------------------------MNNPNIINQFPSLRS-KSYSRVN----------
Cia2_Sac_cer    MSEFLNENPDILEENQLPTRKEDSTKDLLLGGFSNEATLERRSLLLKIDHSLKSQVLQDI
Cia2_Neu_cra    ---------------------------MAKSDLDNANPTVLSVSQLPSRNLAKGHVRKGP
Cia2B_Hom_sap   ---------------------------MVGGGGVGGGLLENANPLIYQ------------
Cia2A_Hom_sap   ---------------------------------MQRVSGLLSWTLSRVLWLSG-------
Cia2_Gia_lam    ---------------------------------MAPHAPYTAGPFFNRGR----------
Cia2_Tri_vag    -------------------------------MAANPNPVVYGSAKYVR------------
Cia2_Cry_par    ------------------------------------------------------------

Cia2_Tra_Hom    ---------------------------------EQFDP---NKLTKGMVF----------
Cia2_Enc_cun    ---------------------------------ISMSNGVLQNVTQRSVF----------
Cia2_Enc_int    ---------------------------------IDISDGILQEITQYSVF----------
Cia2_Nos_cer    ---------------------------------LHFKDKLLTDISVDSVF----------
Cia2_Ent_bie    ---------------------------------LDFENGYLREVTAEAIF----------
Cia2_Sac_cer    EVLDKLLSIRIPPELTSDEDSLPAESEDESVAGGGKEEEEPDLIDAQEIY----------
Cia2_Neu_cra    DSKYDHILFPKQWWAGSSLNTDPSVWTSDEDEDDDLTLATEEPIDEQEIYGENYDPSSCT
Cia2B_Hom_sap   ------------------------RSGERPVTAGEEDEQVPDSIDAREIF----------
Cia2A_Hom_sap   -----------------------------LSEPGAARQPRIMEEKALEVY----------
Cia2_Gia_lam    -------------------------------------PEDYEPITPEEVF----------
Cia2_Tri_vag    ------------------------------STEDDLDSPEREAIDSLELY----------
Cia2_Cry_par    -----------------------------------------------DVY----------

Cia2_Tra_Hom    ELIRHIKDPEHP-YSLEILNVVNLDSIEIKEISTTY------GKNLQQVVVHFQPTIPHC
Cia2_Enc_cun    ELIRDIRDPEHP-YTLEQLGVVSREGVSIGCIGPDG-IAPNVGLPIRCVKVVFKPTIPHC
Cia2_Enc_int    ELIRDIRDPEHS-YTLEQLGVVSREGITIGLIDSDG-IAPSAGLPIKYIKVMFKPTIPHC
Cia2_Nos_cer    ELIRDIKDPEHP-YTLEELNVVRKDLIKIYQLKDEY-VVEDI---INCIEVQFEPTIPHC
Cia2_Ent_bie    ELIRDIQDPEHP-YTLEDLGVVSLSDIKIYTVYNNTNIKCTDGFPLKFIEVQFTPTVPHC
Cia2_Sac_cer    DLIAHISDPEHP-LSLGQLSVVNLEDIDVHDSGNQN--------EMAEVVIKITPTITHC
Cia2_Neu_cra    YLLSTISDPEHP-VTLGQIAVVRLDDIHLSPSPAER----LDPNTLTNVEVDLTPTVNHC
Cia2B_Hom_sap   DLIRSINDPEHP-LTLEELNVVEQVRVQVSDPE-------------STVAVAFTPTIPHC
Cia2A_Hom_sap   DLIRTIRDPEKP-NTLEELEVVSESCVEVQEINEEE----------YLVIIRFTPTVPHC
Cia2_Gia_lam    DIIRSVRDPEHMNMTLEDLRVVNLNDITVMDEQG-------------LVRVVYTPTTPTC
Cia2_Tri_vag    NYIRLIKDPEHP-FSLEQLHIVSPDDIKVDDKEGR-------------VNLVFTPTVPNC
Cia2_Cry_par    ECIKDIIDPEYP-LTLEQLNVVSLENIIIN-------------HEEQIIFVFFKPTVTSC

Cia2_Tra_Hom    SMAAIIGLCIFYVLKARL-DTFWIRVQIAE--DTHVNWKTINKQLDDKDRTNAAFENTSI
Cia2_Enc_cun    SMAAVIGLCIKTHVSRHV-RNHFVQVHIVD--GGHINFRALNKQLDDKDRVLAATENEVL
Cia2_Enc_int    SMAAIIGLCIKAQINQYI-ENHFIQVHIVN--DGHINFKALNKQLDDKDRVLAAMENETL
Cia2_Nos_cer    SMAAIIGLIIKILLEKYI-KGYYIIVSILE--GSHVNDKMLNKQLKDKDRVQAASENEAL
Cia2_Ent_bie    SLVGIIGLSIAYQLYKHT-RNYVIKLRITK--GSHHQEEIYNKQLNDRERVFAAFENESI
Cia2_Sac_cer    SLATLIGLGIRVRLERSLPPRFRITILLKK--GTHDSENQVNKQLNDKERVAAACENEQL
Cia2_Neu_cra    SLATVIGLAVRVRLENALPPNYR--IIVRMKDGSHAQDDQVNKQLGDKERVAAALENDTL
Cia2B_Hom_sap   SMATLIGLSIKVKLLRSLPQRFKMDVHITP--GTHASEHAVNKQLADKERVAAALENTHL
Cia2A_Hom_sap   SLATLIGLCLRVKLQRCLPFKHKLEIYISEG--THSTEEDINKQINDKERVAAAMENPNL
Cia2_Gia_lam    SLGSIIGLSLKIKLDRCLPRRFCSVVYCKD--GTHENAISLNKQINDKERALAALTNKNI
Cia2_Tri_vag    SLPAVLGLCIRERLLQVLPQRFHSKIFITVARGKHIQEDSINRQLRDKERCLAALERRNI
Cia2_Cry_par    SQASLIGLSLYYKLHTVFNKNFKIIIKVVK--GTHDLEDSINKQLKDKERVHAALENPQI
```

```
Cia2_Tra_Hom   LNLIGDCIGPCSV--------
Cia2_Enc_cun   LDLMEKCLPAI----------
Cia2_Enc_int   LDLMKECLPRHGELLDMPK--
Cia2_Nos_cer   LEIIDECLVSIIDKYDL----
Cia2_Ent_bie   LEIIENSINK-----------
Cia2_Sac_cer   LGVVSKMLVTCK---------
Cia2_Neu_cra   KGIIEKMLETCV---------
Cia2B_Hom_sap  LEVVNQCLSARS---------
Cia2A_Hom_sap  REIVEQCVLEPD---------
Cia2_Gia_lam   ASVVNTAIRI-----------
Cia2_Tri_vag   RTMIDNCIACDDEEE------
Cia2_Cry_par   YKTITKGLANSDVWEDQSLLY
```

Abbreviations: Tra_hom, *Trachipleistophora hominis*; Enc_cun, *Encephalitozoon cuniculi*; Enc_int, *Encephalitozoon intestinalis*; Nos_cer, *Nosema ceranae*; Ent_bie, *Enterocytozoon bieneusi;* Sa_cer, *Saccharomyces cerevisiae*; Neu_cra, *Neurospora crassa*; Hom_sap1-3, *Homo sapiens*; Gia_lam, *Giardia lamblia*; Tri_vag*, Trichomonas vaginalis*; Cry_par, *Crystosporidium parvum*.

The DUF59 (PF01883) domain identified using SMART (http://smart.embl-heidelberg.de and pfam database: http://pfam.xfam.org) is highlighted in light blue. Yeast mutants where the hyper-reactive cysteine (red – universally conserved across aligned sequences) is replaced by alanine are not viable[37]. Cia2 is part of the CIA targeting complex (with Cia1 and Mms19) which facilitates the insertion of [4Fe-4S] clusters into cytosolic and nuclear apoproteins[38,39]. Humans possess two isoforms of Cia2. CIA2B, together with CIAO1 and MMS19, is required for maturation of the bulk of cytosolic and nuclear Fe/S proteins. CIA2A binds to CIAO1 and IRP2 and is involved in cellular iron regulation. The human CIA targeting complex CIA2B-CIAO1-MMS19 binds to numerous Fe/S proteins presumably reflecting the apoforms[38,39].

h. Hydrogenase-like Nar1

```
Nar1_Tra_hom    MRRP-----------HMNKKINPCIK------------------------
Nar1_Enc_cun    MDAL--IRPPMSFFADLPKDNKKCIK------------------------
Nar1_Enc_int    MSFF----------AGLSKNNQKCIK------------------------
Nar1_Nos_cer    MNT----------------ENKPCI-------------------------
Nar1_Sac_cer    MSAL----LSESDLNDFISPALACVKPTQ------VSGGKKDNVNMNGEY
Nar1_Neu_cra    MSAI----LSVDDLNDFISPGVACIKPIETLPTAAPPAGDANSSLEVEVI
Nar1_Hom_sap    MASPFSGALQLTDLDDFIGPSQECIKPVKVEKRA-GSGVAKIRIEDDGSY
Nar1_Tri_vag    MSAD-----------PAASTSFDCLHPVSIEE------------------
Nar1_Gia_int    MSLK------VKVASDLNTLPEECVVPLKPADAP-STGTVKLRLK-----
Nar1_Ent_his    MSLS--VGLQIAGVDDYIQQNLVCVMPLKETPP--QEHKGAAKISLGGP-
Nar1_Cry_par    MFST---AVKLANLDDYLESSQDCIVSLLSDK---DDTKPKIAVMRPAKA

Nar1_Tra_hom    ----------GEKPFELTLNDCLACSGCV-SMEETKITAE----DLQ---
Nar1_Enc_cun    ----------IGSPLALSLSDCLACSGCV-SADEAGALSEDLS-FVL---
Nar1_Enc_int    ----------TDTPFNLSLSDCLACSGCV-TTDEAGALSADIS-FVR---
Nar1_Nos_cer    ------------SPFTFKLEDCLACSACI-ADFSVPKIPTY--TELK---
Nar1_Sac_cer    ---EVSTEPDQLEKVSITLSDCLACSGCI-TSSEEILLSSQSHSVFLKNW
Nar1_Neu_cra    LDGQQPEAKSNAPPAEISLTDCLACSGCV-TSAEAVLVSLQSHNEVLNML
Nar1_Hom_sap    FQINQDGGTRRLEKAKVSLNDCLACSGCI-TSAETVLITQQSHEELKKVL
Nar1_Tri_vag    ---RGRVKADDEATFKVTLQDCLACSGCAITKDEITIISEQNTSRIFEKL
Nar1_Gia_int    ---ACDAAPVSSTPVKITINDCLMCSGCV-TSAEEVFFRELNTTALQNAI
Nar1_Ent_his    ---EEGNELPKLTKVTVRLEDCLACSGCI-TSAETVLIEQQGLPEFRKNI
Nar1_Cry_par    QGNKDDKKSGTSDKATVNVADCLACSGCV-TSAEAKLLEDQNVSEFMNIL

Nar1_Tra_hom    --------------------------NVPINLIMSHYSVLNLCNEIKKQQ
Nar1_Enc_cun    ------------------------DLSPQTSFVLSPQSKINIFNLYR---
Nar1_Enc_int    ------------------------DSAPQTSFVLSPQSKVNIFNIYG---
Nar1_Nos_cer    --------------------------DVPLTFLLSPHSKMNMYAHYN---
Nar1_Sac_cer    GKLS-------------------QQQDKFLVVSVSPQCRLSLAQYYG---
Nar1_Neu_cra    DSAPALKLVGPDANGKHSVQGLENSDAKLYVASVSPQSRASLAAACG---
Nar1_Hom_sap    DANKM----------------AAPSQQRLVVVSVSPQSRASLAARFQ---
Nar1_Tri_vag    ------------------------DEVKDYIVLVATHVVANLAAVRN---
Nar1_Gia_int    TSGP--------------------KAGRPIVLSLSQSAILSLSRVL----
Nar1_Ent_his    KEL---------------------SQRKKVICTIADECIASMSVVHN---
Nar1_Cry_par    ------------------------KQKRLTVVSISNQSCSSFACHLN---

Nar1_Tra_hom    LKQNKDDSTNLHAITVKEWYA-YLQNALRRKFSVK--------R------
Nar1_Enc_cun    ----------EDGMEYREFEA-VLSSFLRSKFNIH--------R------
Nar1_Enc_int    ----------KGNMSYREFEG-ALSSFLRAKLNVR--------R------
Nar1_Nos_cer    ----------NHIMSFSDFEY-HLISFIKLKFNVI--------K------
Nar1_Sac_cer    -------------LTLEAADL-CLMNFFQKHFQCK--------Y------
Nar1_Neu_cra    ----------NG-VTEQQAGR-MIEQLFLGEQGLARGGKWGNKF------
Nar1_Hom_sap    -------------LNPTDTAR-KLTSFF-KKIGVH--------F------
Nar1_Tri_vag    -------------WSAAKAFS-TIKQLF-L-------SKGAQKV------
Nar1_Gia_int    -------------LDITSVEPCTVDTLFK-QLEYALRTRVAGLRHCTYED
Nar1_Ent_his    -------------QPFNVVWT-RVEKAL-KKEGVD--------E------
Nar1_Cry_par    -------------CDLITIQR-KLSGLF-KHIGAR--------F------
```

```
Nar1_Tra_hom    -----IVNTYDYQQLSNYMIYNE-----------L--LTKNKLILSECPG
Nar1_Enc_cun    -----IVDTSYLRSKIYEETYRE-----------Y--MATNHLIVSACPG
Nar1_Enc_int    -----IVDTSSMRRKIYKEIYKE-----------Y--LATDHLVISACPG
Nar1_Nos_cer    -----VYDTSYVKNILYDSVYE------------E--STRDKIIISDCPG
Nar1_Sac_cer    -----MVGTEMGRIISISKTVEKI--IAHKKQKENTGADRKPLLSAVCPG
Nar1_Neu_cra    ---TWVVDTNTAREATLVLGSDEV--LGGLIAPSD--KAATPVLTASCPG
Nar1_Hom_sap    -----VFDTAFSRHFSLLESQREF--VRRFRGQAD-CRQALPLLASACPG
Nar1_Tri_vag    -----VLDTD-IQLVFRRLVVKEF--IEN--------QTLSPFMISRCAG
Nar1_Gia_int    APPVYVVSEAQHQEQSVLMNVRQISFLMQS---SE--PRSNIAIITHCPA
Nar1_Ent_his    -----LRDLSQAQDISLFGIYDEF--KEYQ-------KMNKVLLTSTCPG
Nar1_Cry_par    -----VMNSTISEYISLLETKYEF--ISRYKA-----KSDLPMIISHCPG

Nar1_Tra_hom    VVAYVERRR-PDLIPYLSEVPSLQQMCAYLCEEEG---------------
Nar1_Enc_cun    VVTYIERTA-PYLIGYLSRVKSPQQMAFSLVKG-----------------
Nar1_Enc_int    VVAYVERTA-PHLIDYLSRVKSPQQMAFSLVKG-----------------
Nar1_Nos_cer    TVSYIERQA-HHLIEYLSVTSTQQQAIKLLAQ------------------
Nar1_Sac_cer    FLIYTEKTK-PQLVPMLLNVKSPQQITGSLIRATFESLAI----------
Nar1_Neu_cra    WVCYAEKTH-PYVLPHLSRVKSPQALMGTLLKTSLSRILD----------
Nar1_Hom_sap    WICYAEKTHGSFILPHISTARSPQQVMGSLVKDFFAQQQH----------
Nar1_Tri_vag    SVVYYERKT--SYADHLAQIKPYPQLYAMYEKKILQ--------------
Nar1_Gia_int    VRLFITKRN-RELISYIVSTASPMELFGASYCGIDA--------------
Nar1_Ent_his    WVCYSEKMQGKWMFEYMSKVASSMTIAGMIMKKQNS--------------
Nar1_Cry_par    WICYSEKSLNSSVLPLLSKVRSAQQLQGILIKTLTLEIYNQLLFLYKFRL

Nar1_Tra_hom    -------------------------VLTVGVMQCHDKRLEGGV---RV--
Nar1_Enc_cun    -------------------------SRTVSVMPCQDKKLENGRDGVK---
Nar1_Enc_int    -------------------------DRTVSVMPCQDKKLESGRDGVK---
Nar1_Nos_cer    -------------------------GRSISVIQCYDKFLENSDDVLTT--
Nar1_Sac_cer    ---------------------ARESFYHLSLMPCFDKKLEASRPESLD--
Nar1_Neu_cra    --------------------IAPERIWHLAVMPCFDKKLEASREELTDAV
Nar1_Hom_sap    --------------------LTPDKIYHVTVMPCYDKKLEASRPDFFNQ-
Nar1_Tri_vag    -----------------------STNYVLYIGPCYDRKLEAARFEED---
Nar1_Gia_int    ------------------------SPLLVSIQPCQDRKLEQFRGAAV---
Nar1_Ent_his    ------------------------EIYHVSIQMCFDKKLEATKTYNNI--
Nar1_Cry_par    SNSYRTNMNVKSTFTQNDNFVEQSDIFHVAIMPCHDKKLESTRSSLSLK-

Nar1_Tra_hom    --------------DYVLSSKDIYEICSD--VRINFEVDNNDRDIYDDVG
Nar1_Enc_cun    -------------FDFILTTRGFCKALDS--LGFRRPARAS---------
Nar1_Enc_int    -------------FDHVLTTREFRRVLDE--LEFELFLKAN---------
Nar1_Nos_cer    --------------------YDFYKMILD--LGFLQHNFIK-----GTCE
Nar1_Sac_cer    -----------DGIDCVITPREIVTMLQE--LNLDFKSFLT-----EDTS
Nar1_Neu_cra    WAGDGKPGRGVRDVDCVITSKEVLMLAAS--RGFDFFSLSA--SMPPQTP
Nar1_Hom_sap    -------EHQTRDVDCVLTTGEVFRLLEE--EGVSLPDLEP---------
Nar1_Tri_vag    -------------VDAVLTIAEINDHITE--PTEEIPVKFP---------
Nar1_Gia_int    --------------DVCLTAQEVHSFLAGTPQGSSPPA---------FCS
Nar1_Ent_his    -----------HVIDCVLTTSEIDSIIDW--NE-----------------
Nar1_Cry_par    ---SSDKNSSCPEVDIVLATSEVGEIIKL--AGFNSLLDVP--------E
```

```
Nar1_Tra_hom    VGNEGKGDNNGRDIHDDVGVGNEGKGDNNGRDTYD---------------
Nar1_Enc_cun    GKSLCS--------------------------------------------
Nar1_Enc_int    SWNHHE--------------------------------------------
Nar1_Nos_cer    KWEKCT--------------------------IGY---------------
Nar1_Sac_cer    LYGRLS--------------------------------------------
Nar1_Neu_cra    RFPDQL-------------------------I------------------
Nar1_Hom_sap    APLDSL-------------------------C------------------
Nar1_Tri_vag    ADTDLN--------------------------------------------
Nar1_Gia_int    SYTPSP--------------------------------------------
Nar1_Ent_his    ----PI--------------------------------------------
Nar1_Cry_par    APLDNL-------------------------WLNQNFQITKKHNLSLLIT

Nar1_Tra_hom    ----------DVGVGNEGKGDNNGRDIYDDVGVNGKDNNIYETCNGVHTN
Nar1_Enc_cun    ----------MEE---------AETTQW----------------------
Nar1_Enc_int    ----------IED---------MEVTQW----------------------
Nar1_Nos_cer    ----------HYG---------YLEHILNKKNNYLTKNDSIKIFNSK---
Nar1_Sac_cer    ----------PPG---------WDPRVH------------------W---
Nar1_Neu_cra    -----HDFLFRPG---------HRQQ------------------------
Nar1_Hom_sap    -----SG---ASA---------EEPT------------------------
Nar1_Tri_vag    ----------AIS---------QKLGQI----------------------
Nar1_Gia_int    --------------------------TS----------------------
Nar1_Ent_his    ----------------------NEITST----------------------
Nar1_Cry_par    ENYVSNQILNQFS---------WLIPSY----------------------

Nar1_Tra_hom    DKDDNNTNTHNDRLSPEQHSKNSSEHVLTNEKEFLTGITPYLIKM-----
Nar1_Enc_cun    -------------NIGT-SSGGYAEFILG------KHC------------
Nar1_Enc_int    -------------NIGT-SSGGYAEFILS------KHR------------
Nar1_Nos_cer    -----------------------------NEEVRLNGKRKL---------
Nar1_Sac_cer    ----------AS-NLGG-TCGGYAYQYVT------AVQRLHPGSQ-----
Nar1_Neu_cra     -----------S-REAG-TSGGNMHFILR------HLQAKNPGSQ-----
Nar1_Hom_sap    -----------S-HRGG-GSGGYLEHVFR------HAARELFGIHV----
Nar1_Tri_vag    -------------KDSL-NSDSIYQLIAE------IEP------------
Nar1_Gia_int    -------------FWQY-ALGPLLVLY-L------RAKEWISDEGLSRLL
Nar1_Ent_his    -------------RMKG-FISSPAQYIAL------MEQKKE---------
Nar1_Cry_par    --------------FNS-NSGGFCEYIIR------SAIKELAGDHI----

Nar1_Tra_hom    --------LNLTIERIQTI----------NKSYRVLHFK-------NSSL
Nar1_Enc_cun    ---------VVETREIRNGI-------------KEH-LLDD-G------R
Nar1_Enc_int    ---------SVKKIKDRNGI-------------REY-MVDD-G------G
Nar1_Nos_cer    ---------NINTVTLNKINIPEIDYKYKNGVFTYTQIKN------NKKK
Nar1_Sac_cer    --------MIVLEGRNSDIV-------------EYR-LLHD-D---RIIA
Nar1_Neu_cra    --------IQTVPGRNADVV-------------EYK-LIAEAG---EVMF
Nar1_Hom_sap    ------AEVTYKPLRNKDFQ-------------EVT-LEKE-G---QVLL
Nar1_Tri_vag    ---------TLNEEEINSLI-------------SEL-PSRF-----DLEI
Nar1_Gia_int    VQRADGVDLHWTKIGNELFS-------------CTI-ELSQ-NQSPYSCV
Nar1_Ent_his    ---------PFKVTRNKDFL-------------END------G-------
Nar1_Cry_par    ---DNKVQLPFNKLKN-DIL-------------EAK-YIKN-N----VEL
```

```
Nar1_Tra_hom    TFAHITGLKNLLNFLND---------------------------------
Nar1_Enc_cun    TISQITGLENSINYFKSSKT------------------------------
Nar1_Enc_int    IVSQITGLENSINYFKISKT------------------------------
Nar1_Nos_cer    KYLRILGLEPFLNFIKESKH------------------------------
Nar1_Sac_cer    AASELSGFRNIQNLVRKLTSGSGSERKRNITALRKRRTGPKANSREMAAA
Nar1_Neu_cra    KAARYYGFRNIQNLVRKLKP-------------AKTSRMPGGKPFGSAKR
Nar1_Hom_sap    HFAMAYGFRNIQNLVQRLKR------------------------------
Nar1_Tri_vag    STNSFDGETLNKRLTKTLDMM-------------SSG-------------
Nar1_Gia_int    IIYKSTGYHNLQNLVRRIHA-------------LCP--------------
Nar1_Ent_his    -IAIANGFRNIQNVVRFVK-------------------------------
Nar1_Cry_par    NYCLAYGFRAIQSISRKLNL-------------QKNASQ-------NTQY

Nar1_Tra_hom    -----EEMRYNFVDIFLCDDRCFGGPGQIKNN-VQNDY-AHYFKLV----
Nar1_Enc_cun    -----KGPRHKMTEIFLCKNGCIGGPGQERVNDVEMDI-REY-DRN----
Nar1_Enc_int    -----KGPKYKMAEVFLCKNSCIGGPGQERINSVEVDS-AEY-NIY----
Nar1_Nos_cer    -----KELEYDVCEIYICNQGCINGPGQLYTDNLYVNN-SEYIDID----
Nar1_Sac_cer    TAATADPYHSDYIEVNACPGACMNGGGLLNGEQNSLKR-KQLVQTL----
Nar1_Neu_cra    PAGKASGLDYGYVEVMACPGGCTNGGGQIKVDDQVVVDRKGLAVKPGPQE
Nar1_Hom_sap    -----GRCPYHYVEVMACPSGCLNGGGQLQAPDRPSRELLQHVERL---Y
Nar1_Tri_vag    --KKVPKPAPRLAQIDFCKGGCLVGGGQIRGNSPAQRR-A-LIAATQ---
Nar1_Gia_int    -----NKSALYILDLHACPYGCYGGA-CIAGDDRHPVSSV--ASAS--HA
Nar1_Ent_his    -----SKTKLQFIEVEACPGGCICGGGQIKCSPKEKDE-RVK-KMME-IL
Nar1_Cry_par    KQSVVNHVNYHLIEAMACPTGCVSGGGQILSQNDQNDDNSDL-NKL--RK

Nar1_Tra_hom    ---GLNTNEEV------------------------------------IVP
Nar1_Enc_cun    ----GREQPRIFYSS-----------------------------------
Nar1_Enc_int    ----GKEQPEIYYSD-----------------------------------
Nar1_Nos_cer    -----------------------------------------------LGI
Nar1_Sac_cer    ---NKRHGEELAMVD---------------PLT--LGPKLEEAAARPLSL
Nar1_Neu_cra    QKEWQKEVDEAYFSG-----DESGSRAQDESLDLVVDGISPSHIRNVLTH
Nar1_Hom_sap    GMVRAEAPEDAPGVQ------------------------------ELYTH
Nar1_Tri_vag    ---EVHTQNESTNIS---------------------------FPTELYNE
Nar1_Gia_int    ATMSADKAVLSHILAADTCAGL-------------LEGLVQAVDRITLQE
Nar1_Ent_his    EPKVVDEKNKSIYES-----------------------------------
Nar1_Cry_par    NIKFIDEVQEALYKG-----INLN-KNQEV-----ILPDEIPIVNILYEY

Nar1_Tra_hom    QVLKDK---------KRIFENR--------KIYR--------SNFKVEW
Nar1_Enc_cun    -PGLEE---------KRVFREV--------KAKR--------VDLRVDW
Nar1_Enc_int    -PGLEE---------KRTFRPV--------SVKR--------IDFKVDW
Nar1_Nos_cer    DCKQLQ---------KRTYRKI--------ETKK--------VNFKIEW
Nar1_Sac_cer    EYVFAP-----------VKQ----------------AVEKDLVSVGSTW
Nar1_Neu_cra    WSTLTGIQLER--LAYTSYREVVSDVGKEKKMTDTERVVQLAGKIGGGW
Nar1_Hom_sap    WLQGTDSECAGR-LLHTQYHAV----------------EKASTGLGIRW
Nar1_Tri_vag    L--------IKF-GYKTHYESL----------------PQEEEKDQFAW
Nar1_Gia_int    TVIRTD---------GEVVSPE--------EIAARKGIQSGVRIQDLAW
Nar1_Ent_his    -IKDSI---------KLTFIDR--------KESAQ------ENALHLNW
Nar1_Cry_par    LIHIDK-QIDRSSGLKLPFLRN--------DFVSI---NEVPTASSLKW
```

Abbreviations: Tra_hom, *Trachipleistophora hominis*; Enc_cun, *Encephalitozoon cuniculi*; Enc_int, *Encephalitozoon intestinalis*; Nos_cer, *Nosema ceranae*; Sac_cer, *Saccharomyces cerevisiae*; Neu_cra, *Neurospora crassa*; Hom_sap, *Homo sapiens*; Tri_vag, *Trichomonas vaginalis*; Gia_int, *Giardia intestinalis*; Ent_his, *Entamoeba histolytica*; Cry_par, *Crystosporidium parvum*.

Nar1 proteins are related to Fe-only hydrogenases[40] and contain two-conserved cysteine motifs. One is located at the N-terminus and the second is distributed between the central part of the protein and its C-terminus, the cysteine residues forming these motifs are coloured in red and blue, respectively. In yeast, each cysteine motif coordinates a [4Fe-4S] cluster and both are essential for the assembly of cytosolic Fe/S proteins[41]. It has been shown that the C-terminal Fe/S cluster is stably bound to the protein and its assembly depends on the Fe/S cluster from the N-terminal cysteine motif[42]. A conserved C-terminal tryptophan characteristic of the Nar1 protein family is highlighted in yellow.

i. Fe/S protein Dre2

```
Dre2_Tra_hom     MATE----------------------------------------N-----
Dre2_Enc_cun     MEDK----------------------------------------------
Dre2_Enc_int     MEDK----------------------------------------------
Dre2_Ent_bie     MTSS----------------------------------------D-----
Dre2_Sac_cer     MSQYKTGLLL-IHPA--------------------------VTTTP---
Dre2_Neu_cra     MSPITLDLTSDFNPA-NTTGAGSSSSQP-----RTLLVAPPSVASHE---
Dre2_Hom_sap     MADFGISAGQ-FVAVVWDKSSPVEALKGLVDKLQALTGNEGRVSV-----
Dre2_Cry_par     MTQLIITHQ---------SDSKLEES-------EVFLSELNRIKKEEDKF

Dre2_Tra_hom     --DDF---KKLLEG------------------------------------
Dre2_Enc_cun     --EEL---RKLLRS------------------------------------
Dre2_Enc_int     --EEL---RKLLRS------------------------------------
Dre2_Ent_bie     --EEL---KELLKQ------------------------------------
Dre2_Sac_cer     --ELVENTKAQAASKKVKFVDQFLINKLNDGSITLENAKYETVHYLTPEA
Dre2_Neu_cra     --ERI---SALFSTYPRDTTDLHMLDRLAAGLVTLPTSTY----------
Dre2_Hom_sap     --ENI---KQLLQSAHKESSFDIILSGLVPGSTTLHSA------------
Dre2_Cry_par     GKFSS---LSDLRAIVKKGEFRIVSIYLSSGSILGEIF------------

Dre2_Tra_hom     --------------------------------------------------
Dre2_Enc_cun     --------------------------------------------------
Dre2_Enc_int     --------------------------------------------------
Dre2_Ent_bie     --------------------------------------------------
Dre2_Sac_cer     QTDIKFPKKLISVLADSLKPNGSLIGLSDIYKVDALINGFEIINEPDYCW
Dre2_Neu_cra     -----------DLILVLTDPDGSRHAEASALLSNRAVWSLLVPALKAGGK
Dre2_Hom_sap     --------EILAEIARILRPGGCLFLKEPVETAVDNNSKVKTASKLCSAL
Dre2_Cry_par     ------TFEFLKEFYGVLDFGSVLKVNI---LALDSIDKVKAFER---NL

Dre2_Tra_hom     --------------------------------------------------
Dre2_Enc_cun     --------------------------------------------------
Dre2_Enc_int     --------------------------------------------------
Dre2_Ent_bie     --------------------------------------------------
Dre2_Sac_cer     IKMDSSKLNQ------------TVSIPLKKKKTNNTKLQSGSKLPT--FK
Dre2_Neu_cra     LRSEDGTLGRDTTTPEARE---AVLAGL---------VAGADGFTKPDYA
Dre2_Hom_sap     TLSGLVEVKELQREPLTPEEVQSVREHLGHESDNLLFVQITG--KKPNFE
Dre2_Cry_par     LFSGFIKVKKLKGDGLN-----SSDSDF---------------------E

Dre2_Tra_hom     ---------ALKVKNR---R-----TEE----------------------
Dre2_Enc_cun     ---------MMRKSTDP--RT-------------KM--------------
Dre2_Enc_int     ---------TIRKNTDP--RV-------------KI--------------
Dre2_Ent_bie     ---------ATTYKQDP--R------------------------------
Dre2_Sac_cer     KASSSTSNLPSFKKADHSRQPIVKETDSFKPPSFKMTTEPKVYRVVDDL-
Dre2_Neu_cra     EEEAVPLRFGLKRKTNP--NPVVAP—IQPVAQVVTAAPAGVGFVTLDL--
Dre2_Hom_sap     VGSSRQLKLSITKKSSP--SVKPAVDPAAAKL-------------WTLS-
Dre2_Cry_par     IVIKAEKP--SWKPEE----------------------------------

Dre2_Tra_hom     ----------D-----------------EKDF--LD-EDA----------
Dre2_Enc_cun     ----------Q-----------------DEYL--TD-EDK---AIQ----
Dre2_Enc_int     ----------S-----------------DEYL--TD-EDK---AET----
Dre2_Ent_bie     ----------H-----------------RIPM--VDRPSK----------
Dre2_Sac_cer     ----IEDSDDDDFSSDSSKAQYFDQVDTSDDS--IE-EEE-LIDEDGSGK
Dre2_Neu_cra     NDDLDLDGEDD-----------------DDDV--ID-EDT-LLTEADLRR
Dre2_Hom_sap     ----ANDMEDD-----------------SMDL--ID-SDE-LLDPEDLKK
Dre2_Cry_par     ----------G-----------------KVLVDDID-LEGSVPDIKNYVP
```

```
Dre2_Tra_hom    ---SE--SVSNSRRVQKKKRACENCVCGRAEGKKKLSREELKKM------
Dre2_Enc_cun    -----------RSERPPAKKRACKDCTCGLKEEQEV---------------
Dre2_Enc_int    -----------KTLRTPAKKRACKDCTCGLKEKQEV---------------
Dre2_Ent_bie    ---I---LTQSNKCRENKARKCSNCTCNKNTNTNNT--------------
Dre2_Sac_cer    S---MITMITCGKSKTKKKACKDCTCGMKEQEENEINDI-RSQQDKV--
Dre2_Neu_cra    P---IQQPPECQPKPGKKRRACKDCTCGLAERLEAEDKAR-RDKADQALN
Dre2_Hom_sap    PDPASLRAASCGEG--KKRKACKNCTCGLAEELEKEKS---REQ------
Dre2_Cry_par    ---LGQ----GKESCKSKERACNNCNCGRADLEKEIGVEAARKV------

Dre2_Tra_hom    ----SREEV-------KKLANAGCGGCKMGDAFRCGDCPFYGLPSFEEGD
Dre2_Enc_cun    ------------------RTRSACGNCYKGDAFRCSGCPSLGLPPYEPGD
Dre2_Enc_int    ------------------EVRSACGNCYKGDAFRCSGCPSLGLPPYEPGE
Dre2_Ent_bie    ------------------IYKSKCGSCHLGDPFRCSSCPYKGLPPFNEGD
Dre2_Sac_cer    -VKFTEDELTEIDFTIDGKKVGGCGSCSLGDAFRCSGCPYLGLPAFKPGQ
Dre2_Neu_cra    TLKLKSEDLLELDLTV-PGKTGSCGSCALGDAFRCAGCPYLGLPPFKVGE
Dre2_Hom_sap    --------M-------SSQPKSACGNCYLGDAFRCASCPYLGMPAFKPGE
Dre2_Cry_par    ----YQEKVE------TGTARSSCGNCYLGDAFRCSGCPYKGMPAFKPGE

Dre2_Tra_hom    EVFFD---------------------------------------------
Dre2_Enc_cun    VVSFSMDL--------SNE-----------------------------FQ
Dre2_Enc_int    VVSFSTDL--------DEG-----------------------------LQ
Dre2_Ent_bie    EINFD---------------------------------------------
Dre2_Sac_cer    PINLDSIS--------D---------------------------------
Dre2_Neu_cra    EVSILNNV--------P---------------------------------
Dre2_Hom_sap    KVLLSDSN------------------------------------------
Dre2_Cry_par    KVSLANAEGDANDHTVDMNLIHEEKVDLITTTFDDDGSGVNNVQSKGGVL

Dre2_Tra_hom    ----
Dre2_Enc_cun    GEDA
Dre2_Enc_int    GQDG
Dre2_Ent_bie    --EL
Dre2_Sac_cer    --DL
Dre2_Neu_cra    --QL
Dre2_Hom_sap    LHDA
Dre2_Cry_par    KLNI
```

Abbreviations: Tra_hom, *Trachipleistophora hominis*; Enc_cun, *Encephalitozoon cuniculi*; Enc_int, *Encephalitozoon intestinalis*; Ent_bie, *Enterocytozoon bieneusi*; Sac_cer, *Saccharomyces cerevisiae*; Neu_cra, *Neurospora crassa*; Hom_sap, *Homo sapiens*; Cry_par, *Crystosporidium parvum*.

Dre2 is an essential protein in *Saccharomyces cerevisiae* and is conserved among animal species and other eukaryotes[43]. The human protein is called CIAPIN1 or anamorsin. Dre2 homologues have a conserved C-terminal domain (underlined with blue bar) sometimes called a CIAPIN1 motif, which is present in the microsporidian sequences. In yeast the amino acids 173-348 within the CIAPIN1 motif interact with the FMN- and FAD-binding domains of Tah18. The protein contains an N-terminal [2Fe-2S] and a C-terminal [4Fe-4S] cluster within the CIAPIN1 motif. The coordinating cysteine residues are marked in red[44]. In yeast, Tah18 transfers electrons to the [2Fe-2S] cluster of Dre2[29]. In human and yeast the C-terminus of the proteins are connected to an N-terminal S-adenosylmethionine (SAM) methyltransferase-like domain which is not known to bind SAM[45]. The SAM-like domain is not present

in the microsporidian Dre2-like proteins and was not detected using CDD at the NCBI[16]. Notably, residues 1-172 of this region are known to be important for Dre2 function in Fe/S protein biogenesis, yet the region is not essential for yeast cell viability[43-45].

In yellow: Acidic and serine (E, D and S)-rich patch identified in human and yeast Dre2[43]. The E, D and S residues in this region of microsporidian proteins were also labelled in yellow for comparison.

j. Diflavin-oxidoreductase Tah18

```
Tah18_Tra_hom  ----------------------------------------MSVQIPIVYGTQSGNSLHVS
Tah18_Enc_cun  -------------------------------------------MIPILYGSQTGTAIYVS
Tah18_Enc_int  -------------------------------------------MIPILYGSQTGTSIYVS
Tah18_Nos_cer  --------------------------------------------MIILYGSQTGNSIHIA
Tah18_Sac_cer  --------------------------------------MSSSKKIVILYGSETGNAHDFA
Tah18_Neu_cra  ------------------------MGEPVLAASVAKSTTMEGRNLILLYGSETGNSEEIA
Tah18_Hom_sap  ---------------------------------------MPSPQLLVLFGSQTGTAQDVS
Tah18_Cry_par  MPFFYINNALNYLSICNISIGFILVLKKLAQLSSEYIENNVPSNFSIFYSTETGNSRKIS

Tah18_Tra_hom  QLIRSKLTH-YTTHIVP-------------------VDEFLLENFLNCNTFIFVCSTYGN
Tah18_Enc_cun  NLIARAIMHGYDAKTIYNLDAFLYSPGQKDACLVMEMDLLDIEKILDIDLIIFVCSTHGD
Tah18_Enc_int  NLIERALLYGYDPKTIYNLDCFFNSRDQENLSSVMEMDLFDIEKILDIDFIIFVCSTHGD
Tah18_Nos_cer  KLIQNVILYGYNKDLIYNVDKELLPTD-----FTLDMDSFDFEKILDIDMIIFVCSTHGN
Tah18_Sac_cer  TILSHRLHRWHFSHTFCS------------------IGDYDPQDILKCRYLFIICSTTGQ
Tah18_Neu_cra  MELAKMAERLHFNTVVG------------------EMDDFKLTDLLRYSLAIFVTSTTGQ
Tah18_Hom_sap  ERLGREARRRRLGCRVQ------------------ALDSYPVVNLINEPLVIFVCATTGQ
Tah18_Cry_par  ELFKKLLDEISIEANVREIN---------------SILEENMYLNSNNSVFVFVVSTCGN

Tah18_Tra_hom  GDFPFAA--------QYFYNCLVSESVPNDFLKKLVRACPTIFLKSCNIAVFGLGDSSYA
Tah18_Enc_cun  GAEPFNM--------TKFWSFLSRDDLPSTILSHLS------------FAVFGLGDSSYE
Tah18_Enc_int  GTEPFNM--------TKFWSFLSNSDLPGNLLSHLN------------FAVFGLGDSSYE
Tah18_Nos_cer  GSEPFNM--------TKFWKFLRKKNLPTNFLQHLN------------FAVFGLGDSSYK
Tah18_Sac_cer  GELPRNVNALKGERPVTFWSFLKRKNLPSNLLNHIQT------------AMLGLGDSSYP
Tah18_Neu_cra  GDMPKNT--------TTLWKSLRRTKLNNTNCLAPVK-----------FSIFGLGDSSYP
Tah18_Hom_sap  GDPPDNM--------KNFWRFIFRKNLPST-ALCQMD-----------FAVLGLGDSSYA
Tah18_Cry_par  GSFPASS--------RKFIRYLSKMIKSGNEIFLGIK-----------YTIIGLGSSLYE

Tah18_Tra_hom  KFNFASKKLFKVFS-KLGANLFVERGNGNVQDDEG--------YYTALFPWIEKLITNLQ
Tah18_Enc_cun  KFNYCSKRLFNRLR-MLGARPVIRRGSGDSQDREG--------FLSDFRPWLLELTAYLR
Tah18_Enc_int  KFNYCSKKLFNRLR-MLGAKPVVRRGDGNAQDKEG--------FLSDLRPWLLELMAHFD
Tah18_Nos_cer  SFNFCSKKLYNCLL-KHGAKPLIRKGNGDSQDKEG--------FMGEFKTWIKDLYYILP
Tah18_Sac_cer  KFNYGIRKLHQRIVTQLGANELFDRLEADDQAMAGSNKGTGLGIESVYFEYEKKVLSFLL
Tah18_Neu_cra  KFNWAARKLRVRLL-QLGASEFFRPGEADERHENG--------LDSIYLPWYQELRESLL
Tah18_Hom_sap  KFNFVAKKLHRRLL-QLGGSALLPVCLGDDQHELG--------PDAAVDPWLRDLWDRVL
Tah18_Cry_par  YSFNSAALKLDKLISSLKGEKYCEIALLDEVNGNE------IDFKTWWNNTFLNRLGISD

Tah18_Tra_hom  S-LNIKGAELIIKQKR--------------------------------------------
Tah18_Enc_cun  TYISRPCTDFISTQPK--------------------------------------------
Tah18_Enc_int  P-LKIKHIDILSLKPE--------------------------------------------
Tah18_Nos_cer  HYKLQNAKNFASCKND--------------------------------------------
Tah18_Sac_cer  SKYPNRKVNGQIIKREELDPEVYLEPASYLQLS---------------------------
Tah18_Neu_cra  SQFPLPKGIEPIPDDAPLPPKYNIRLVPSTGSLKDKITNGEGHVSQVEDNEQLAARFERM
Tah18_Hom_sap  GLYPPPPGLTEIPPGVPLPSKFTLLFLQEAPS---------------------------T
Tah18_Cry_par  SKEHLNKHFSIAVTRKKDEFVCKLRSINEISLYN--------------------------

Tah18_Tra_hom  -------------------------------LYDATIISKNMLTPCDHFQEILEVGLSVE
Tah18_Enc_cun  -------------------------------RYVSRLVEKRVLTPDDHFQKIVEFVFDIP
Tah18_Enc_int  -------------------------------KYTSRLVGKRLLTPEGHFQKIIELVFEIP
Tah18_Nos_cer  -------------------------------LYSASINDIKILTPYNYIYPILEIKFDI-
Tah18_Sac_cer  -----------DEHANEKFTSTKVIFEGDESLKVGRVNINKRITSEGHFQDVRQFKFSNV
Tah18_Neu_cra  STESEATEAPGQKDGTDVPDFPPAKLLPIPGSFTAQVVCNKRVTPEDHWQDVRHIEFELR
Tah18_Hom_sap  GSEGQRVAHPGSQE-------PPSESKP----FLAPMISNQRVTGPSHFQDVRLIEFDIL
Tah18_Cry_par  ------------HSLSGNCHVSEIYFKLIEFCPINRELLCETKTIDGIERQIFNLKLELP
```

```
Tah18_Tra_hom  NYDDFE-----PGDTIRIYPSN--YNWREFCD-YIGNVDDEDHVR--------------N
Tah18_Enc_cun  DYKEFF-----PGDCLSLLPEN--YNYREFMS-YNGIGDGDLDGVSSVWM-------LQN
Tah18_Enc_int  EYKEFS-----PGDCLSFCPEN--YNYKEFMK-YNGM-EEDVDGISSSLM-------MKH
Tah18_Nos_cer  DIENFE-----IGDCLAVYPEN--YNYEEFVR-YNNIKDNTLVKY------------IKK
Tah18_Sac_cer  DKIQEN---YEPGDTVTIYPCNTDEDVSRFLANQSHWLEIADKPLNFTSGVPNDLKDGGL
Tah18_Neu_cra  SPGRNGAMSFAG-QTLLIYPKNYPKDVQKLID-LMGWSEVAEQRIEIDWVKGTRPRDYHF
Tah18_Hom_sap  GSG----ISFAAGDVVLIQPSNSAAHVQRFCQ-VLGLD--PDQLFMLQPREPDVSSPTRL
Tah18_Cry_par  DKVQYK-----TFDIIDILPPNLDENITFFSSKVLGINSIEDLKNITVEFVPINNMTRNI

Tah18_Tra_hom  TMDYN----FVPFFRIFAEIVKYLEDEPLNDYVFKNPERKEAVI-----------RRIRE
Tah18_Enc_cun  VVDFN----SQPHQPFFFALRHFLGQK---------NRTEEEIL-----------LKIEE
Tah18_Enc_int  SIDFN----SQPHQPFFFALRYFLDRK---------GGIEDEVL-----------LKIEE
Tah18_Nos_cer  YCDFN----SIPQIYFFLQLSLITDT------------IAEEYR-----------EKCKE
Tah18_Sac_cer  VRPMT----LRNLLKYHCDFMSIPRTSFFLKIWTFATDVTKMERGQEQLNDQREKLRQFA
Tah18_Neu_cra  LKDAT----IRDVLTHNFDISAVPKRTFLEFMAYHTTNPLEKER-----------LHELT
Tah18_Hom_sap  PQPCS----MRHLVSHYLDIASVPRRSFFELLACLSLHELEREK-----------LLEFS
Tah18_Cry_par  SVPFPNNRSLMHILKYYFDLMTLPPHSVMLQFVPYLNSIEGELISNE--------SFFNE

Tah18_Tra_hom  MSTDYEL-YFDYVKRPKRTFFEVLQDFAL------KLPFSFLKKIVPNIQPRYFTLTKRE
Tah18_Enc_cun  IAQDYDL-YHDYVIRARRTVFEVLKDLRI------KVDIGFLKSFVPAMYPRFFSVTKKK
Tah18_Enc_int  IAQDYDL-YYEYIVKPKRTIFEVLQELRV------KVDARFLKKFVPTIYPRFFSVTKKK
Tah18_Nos_cer  IYLNYDL-YYDYILLPKRTIFEVLKDFKI------KLTSNFMYKYIPVINPRYFTLTKKD
Tah18_Sac_cer  TDQDMQD-LYDYCNRPRRSILEVLEDFIS--V---KLPWKYVLDYLPIIKPRYYSISSGP
Tah18_Neu_cra  QRGDSDE-FYDYTSRPRRTILEVLEDFPG--V---KIPYTRLLEFP-IIRPREFSLCNGG
Tah18_Hom_sap  SAQGQEE-LFEYCNRPRRTILEVLCDFPHTAA---AIPPDYLLDLIPVIRPRAFSIASS-
Tah18_Cry_par  NKDSYEFSFHLFINRFMKSLIPIPIEKFVKFTGIRQYPRSYSISSSSLASPSMIDLTIST

Tah18_Tra_hom  -------------------------SSYYLTIALVEYQNSIKAKRKGLCSQYLREINVK-
Tah18_Enc_cun  -------------------------GLYHITVAIVRYTTFLSEPRRGVCSEYLMSLSLN-
Tah18_Enc_int  -------------------------GLYHVTVAVVNYKTILSQPRRGVCSEYLMGLSLN-
Tah18_Nos_cer  -------------------------FCYFVTVSLVSFKTSLKEERKGLCSEYLKLLTKG-
Tah18_Sac_cer  GD-----------------------PNIELTVAIVKYKTILRKIRRGICTNYIARLQEG-
Tah18_Neu_cra  DPAVNAKDLVISNEQDTTTTTTTDVYKFEILAALVHYRTIIRKPRQGLCSRYLRHLPVG-
Tah18_Hom_sap  ---------------------------LLILVAVVQFQTRLKEPRRGLCSSWLASLDPGQ
Tah18_Cry_par  CIKG------------------QISAPLNEIISEGANKKNSKKIIKGLCSSFLFEFDLN-

Tah18_Tra_hom  --ESIKVGMVKSHLFYDSAN-------LLFICTGTGITLPRAFWN----FFTD----KNI
Tah18_Enc_cun  --DVIKIGVERSNLYFDSDK-------LLFVCTGTGITLPRACVN----EFKD----KEI
Tah18_Enc_int  --DKVPIGIGRSNLYFGSNK-------LLFICTGTGITLPRACIN----EFKD----KEI
Tah18_Nos_cer  --STIHVNIVQNRLNFSGKK-------ILFMCTGTGITLPRAFVN----YYSDLNFFKKV
Tah18_Sac_cer  --EQIRYKLQNNHIIKKEFLN----KPMILVGPGVGLAPLLSVVK--------AEISKDI
Tah18_Neu_cra  --TTVQIGIKPPSSPFAMDDPSFYSRPLIGVATGTGIAPFRALLQDRCLVQEDQQKLGPT
Tah18_Hom_sap  GPVRVPLWVRPGSLAFPETP----DTPVIMVGPGTGVAPFRAAIQERVAQGQTG-----N
Tah18_Cry_par  --LPVLGMIRSSSLNIDNVTS-----SALMFSHGSGIAPIRALLHERKYLINEKKIIKPA

Tah18_Tra_hom  VVFYGYRHDDKDRLYVEEMKKNKNVKVVYAPSRMG-------------------------
Tah18_Enc_cun  VIFYGFRYKNKDFLYPDEWT-GRNVRMFTAASRD--------------------------
Tah18_Enc_int  VVFYGFRYRDRDFLYSDEWN-CKNVRMLTAASRD--------------------------
Tah18_Nos_cer  ILLYGFRFRDVDFLYKEEFE-NKGIEIYPAVSRE--------------------------
Tah18_Sac_cer  KLLFGCRYKDKDYIYKDMLEDWFRKGKIALHSSFSRDEENSP------------------
Tah18_Neu_cra  LLFFGCRNAAADFHFQAEWGTVPN---LTVYPAFSRDNDSSSTEEEETKLALQRAAGIYD
Tah18_Hom_sap  FLFFGCRWRDQDFYWEAEWQELEKRDCLTLIPAFSREQEQ--------------------
Tah18_Cry_par  YLFYGCRTEN-EIIYKDELKDFKRIGALTEVFFALSKT----------------------
```

```
Tah18_Tra_hom  -EELYVQEIFYKYF------HDSIDQYLIYVSGR---TRLNKEVRQMFVKKYG-------
Tah18_Enc_cun  -DKVYVQDVFRKSP------VEDVDQYLIFVSGN---SRLNREVKKLLEDVYG-------
Tah18_Enc_int  -DKMYVQDVFNRNP------IEDIDEYLIFVSGN---SRLNKEVRKLFQKLYG-------
Tah18_Nos_cer  -DNKYIQDIYKTIKG-----LENIDDWLIFVSGN---SRLNNIIEKMLLDIYK-------
Tah18_Sac_cer  -GVKYVQDYLWRLGEEITNLVVNKDAVFFLCGSS---GKMPIQVRLTFIEMLKKWGNFSD
Tah18_Neu_cra  AGKNYVQNQIRQHAAEVGELL-RQNPIIVVCGNS---GRMPKSVREALEDAAVGSGVVAD
Tah18_Hom_sap  --KVYVQHRLRELGSLVWELLDRQGAYFYLAGNA---KSMPADVSEALMSIFQEEGGLCS
Tah18_Cry_par  -QKKYVKDIIPFYKHIILKVVDQNDSIIYICGKKEFVSGIKNEVASIISNNRS-------

Tah18_Tra_hom  ------------AELYFQAETW-
Tah18_Enc_cun  ------------KRIVFQSETW-
Tah18_Enc_int  ------------RTIAFQSETW-
Tah18_Nos_cer  ------------KKIYFQAETW-
Tah18_Sac_cer  EETAKKYLKEMEKSDRYIQETW-
Tah18_Neu_cra  KEEAKGWFDRK-ENCVYWQETW-
Tah18_Hom_sap  PDAAAYLARLQ-QTRRFQTETWA
Tah18_Cry_par  ----RNIIKKMFVEGRIFIESWN
```

Abbreviations: Tra_hom, *Trachipleistophora hominis*; Enc_cun, *Encephalitozoon cuniculi*; Enc_int, *Encephalitozoon intestinalis*; Nos_cer, *Nosema ceranae;* Sac_cer, *Saccharomyces cerevisiae*; Neu_cra, *Neurospora crassa*; Hom_sap, *Homo sapiens;* Cry_par, *Crystosporidium parvum*.

Tah18 is a member of the diflavin oxidoreductase family containing FMN and FAD cofactors that are reduced by cytosolic NADPH. The FMN binding domain of diflavin reductase proteins is located at the N-terminus while the FAD/NADH binding domain is located in the C-terminal domain[46]. The FMN and FAD domains interact with Dre2[45]. *In vivo* Tah18 interacts with the Fe/S CIA component Dre2 and transfers electrons to the [2Fe-2S] cluster of Dre2[29]. Motifs similar to other diflavin reductases: Residues involved in FMN binding are indicated in **bold**. Residues that make hydrogen bonds with phosphate groups of FMN are labelled in red. The tyrosine that stabilizes the FMN prosthetic group is labelled in green[47]. The FMN, FAD and NAD binding domains identified using SMART(http://smart.embl-heidelberg.de) are highlighted in pink (PF00258), yellow (PF00667) and light blue (PF00175) respectively.

Supplementary Figure 2. Phylogenetic trees for mitosomal and cytosolic Fe/S protein biogenesis components and for cytosolic and nuclear Fe/S proteins.

Components of the mitosomal ISC pathway have originated from the mitochondrial endosymbiont. The CIA pathway appears largely bacterial in character, and not archaeal as might be expected given that the host for the mitochondrial endosymbiosis is now thought to have descended from an Archaeon. By contrast, important nuclear and cytosolic Fe/S proteins do appear to have originated from an Archaeon. Monophyly of eukaryotic sequences, including those from microsporidians, is generally observed, suggesting that there is strong negative selection against gene replacement and reflecting the important roles that Fe/S proteins play in eukaryotic physiology.

1 Mitochondrial / mitosomal Fe/S cluster (ISC) assembly components

Mitochondrial Hsp70 (Ssc1 in yeast)

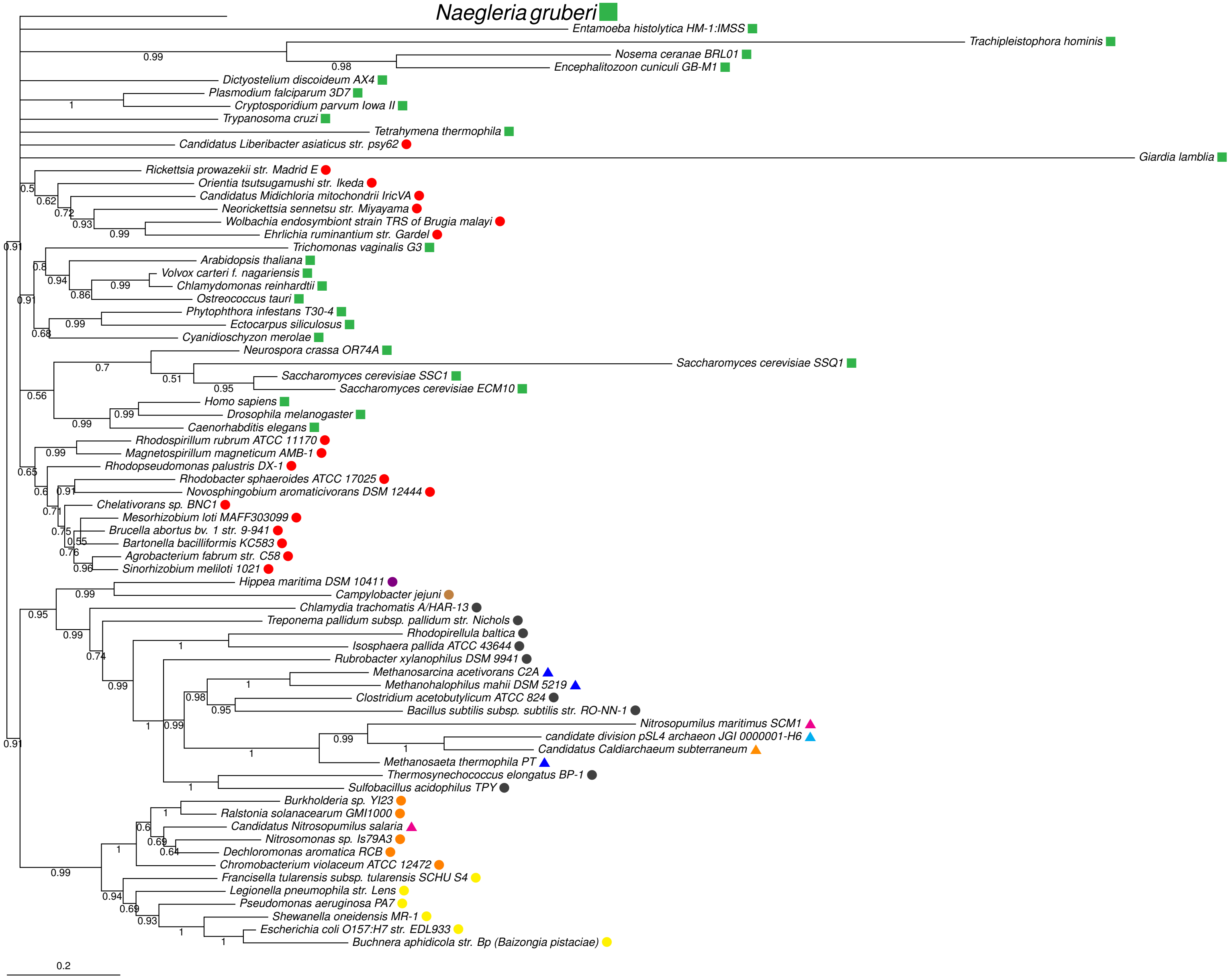

For all trees:

- Alphaproteobacteria
- Betaproteobacteria
- Gammaproteobacteria
- Deltaproteobacteria
- Epsilonproteobacteria
- Other bacterial groups
- Euryarchaeota
- Thaumarchaeota
- Aigarchaeota
- Crenarchaeota
- Korarchaeota
- Eukaryota

Jac1

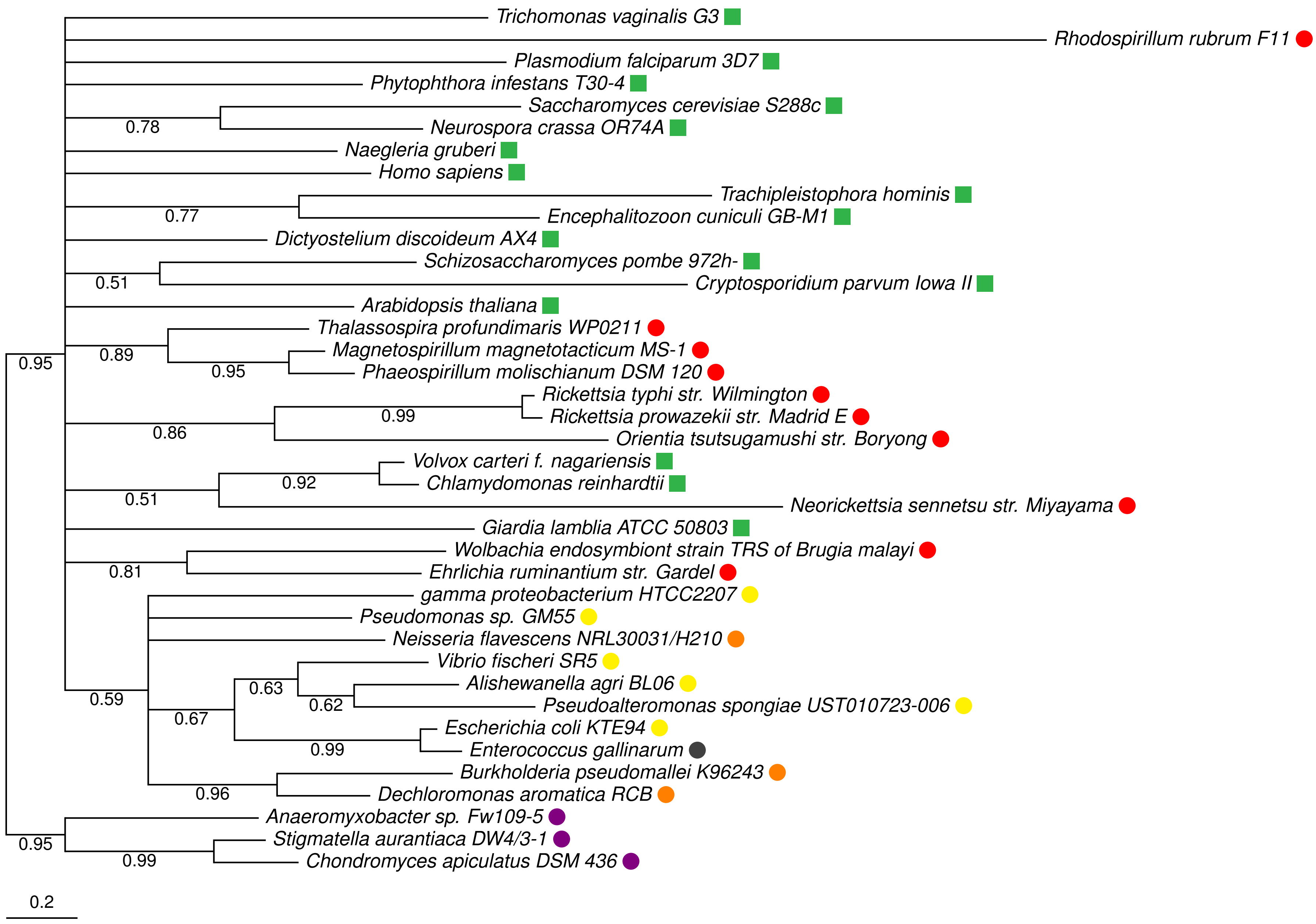
Trichomonas vaginalis G3
Rhodospirillum rubrum F11
Plasmodium falciparum 3D7
Phytophthora infestans T30-4
Saccharomyces cerevisiae S288c
Neurospora crassa OR74A
Naegleria gruberi
Homo sapiens
Trachipleistophora hominis
Encephalitozoon cuniculi GB-M1
Dictyostelium discoideum AX4
Schizosaccharomyces pombe 972h-
Cryptosporidium parvum Iowa II
Arabidopsis thaliana
Thalassospira profundimaris WP0211
Magnetospirillum magnetotacticum MS-1
Phaeospirillum molischianum DSM 120
Rickettsia typhi str. Wilmington
Rickettsia prowazekii str. Madrid E
Orientia tsutsugamushi str. Boryong
Volvox carteri f. nagariensis
Chlamydomonas reinhardtii
Neorickettsia sennetsu str. Miyayama
Giardia lamblia ATCC 50803
Wolbachia endosymbiont strain TRS of Brugia malayi
Ehrlichia ruminantium str. Gardel
gamma proteobacterium HTCC2207
Pseudomonas sp. GM55
Neisseria flavescens NRL30031/H210
Vibrio fischeri SR5
Alishewanella agri BL06
Pseudoalteromonas spongiae UST010723-006
Escherichia coli KTE94
Enterococcus gallinarum
Burkholderia pseudomallei K96243
Dechloromonas aromatica RCB
Anaeromyxobacter sp. Fw109-5
Stigmatella aurantiaca DW4/3-1
Chondromyces apiculatus DSM 436
0.78
0.77
0.51
0.95
0.89
0.95
0.99
0.86
0.92
0.51
0.81
0.59
0.63
0.62
0.67
0.99
0.96
0.95
0.99
0.2

Arh1

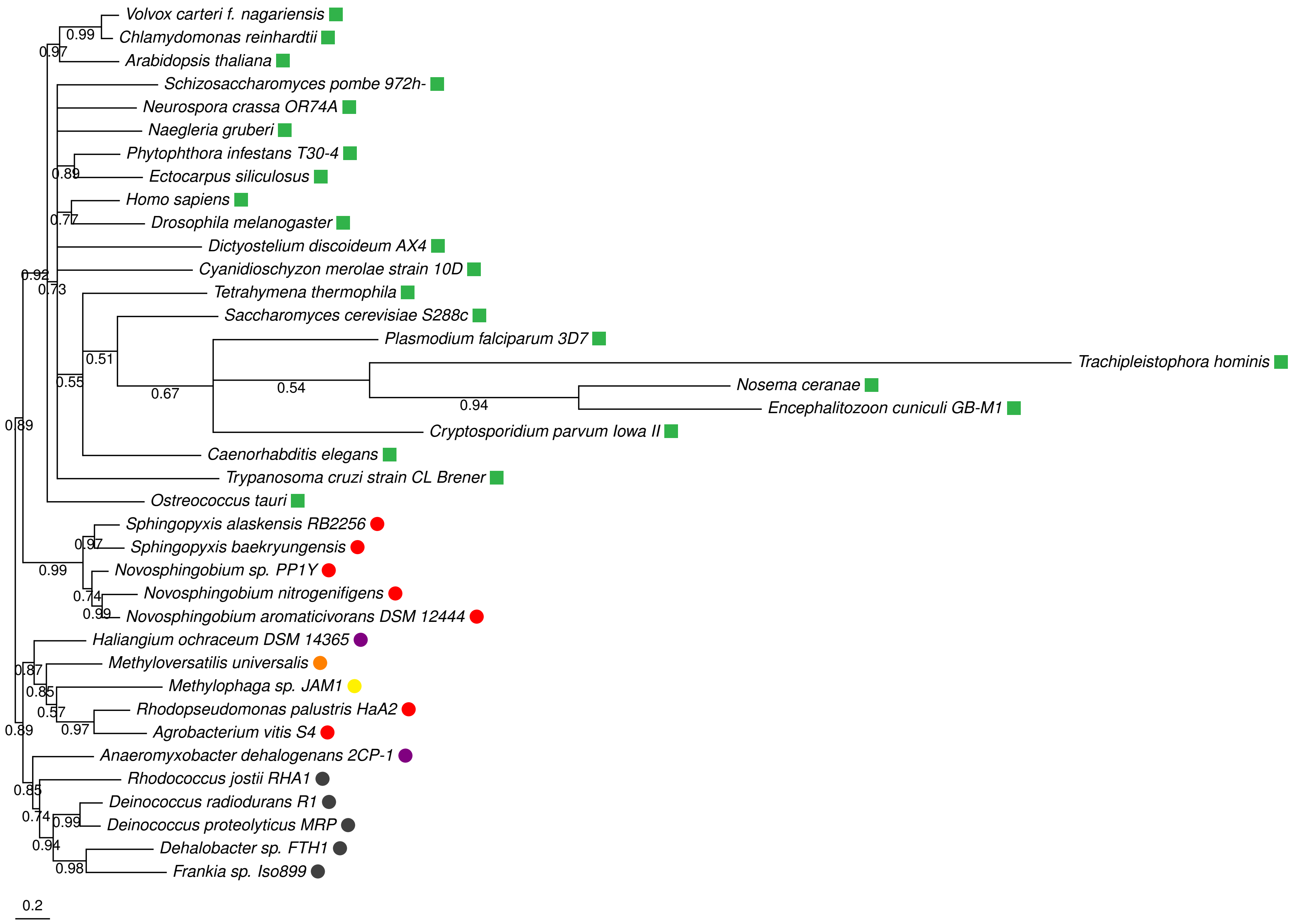
Volvox carteri f. nagariensis
Chlamydomonas reinhardtii
Arabidopsis thaliana
Schizosaccharomyces pombe 972h-
Neurospora crassa OR74A
Naegleria gruberi
Phytophthora infestans T30-4
Ectocarpus siliculosus
Homo sapiens
Drosophila melanogaster
Dictyostelium discoideum AX4
Cyanidioschyzon merolae strain 10D
Tetrahymena thermophila
Saccharomyces cerevisiae S288c
Plasmodium falciparum 3D7
Trachipleistophora hominis
Nosema ceranae
Encephalitozoon cuniculi GB-M1
Cryptosporidium parvum Iowa II
Caenorhabditis elegans
Trypanosoma cruzi strain CL Brener
Ostreococcus tauri
Sphingopyxis alaskensis RB2256
Sphingopyxis baekryungensis
Novosphingobium sp. PP1Y
Novosphingobium nitrogenifigens
Novosphingobium aromaticivorans DSM 12444
Haliangium ochraceum DSM 14365
Methyloversatilis universalis
Methylophaga sp. JAM1
Rhodopseudomonas palustris HaA2
Agrobacterium vitis S4
Anaeromyxobacter dehalogenans 2CP-1
Rhodococcus jostii RHA1
Deinococcus radiodurans R1
Deinococcus proteolyticus MRP
Dehalobacter sp. FTH1
Frankia sp. Iso899
0.99
0.97
0.89
0.77
0.92
0.73
0.51
0.55
0.67
0.54
0.94
0.89
0.97
0.99
0.74
0.99
0.87
0.85
0.57
0.89
0.97
0.85
0.74
0.99
0.94
0.98
0.2

Yah1

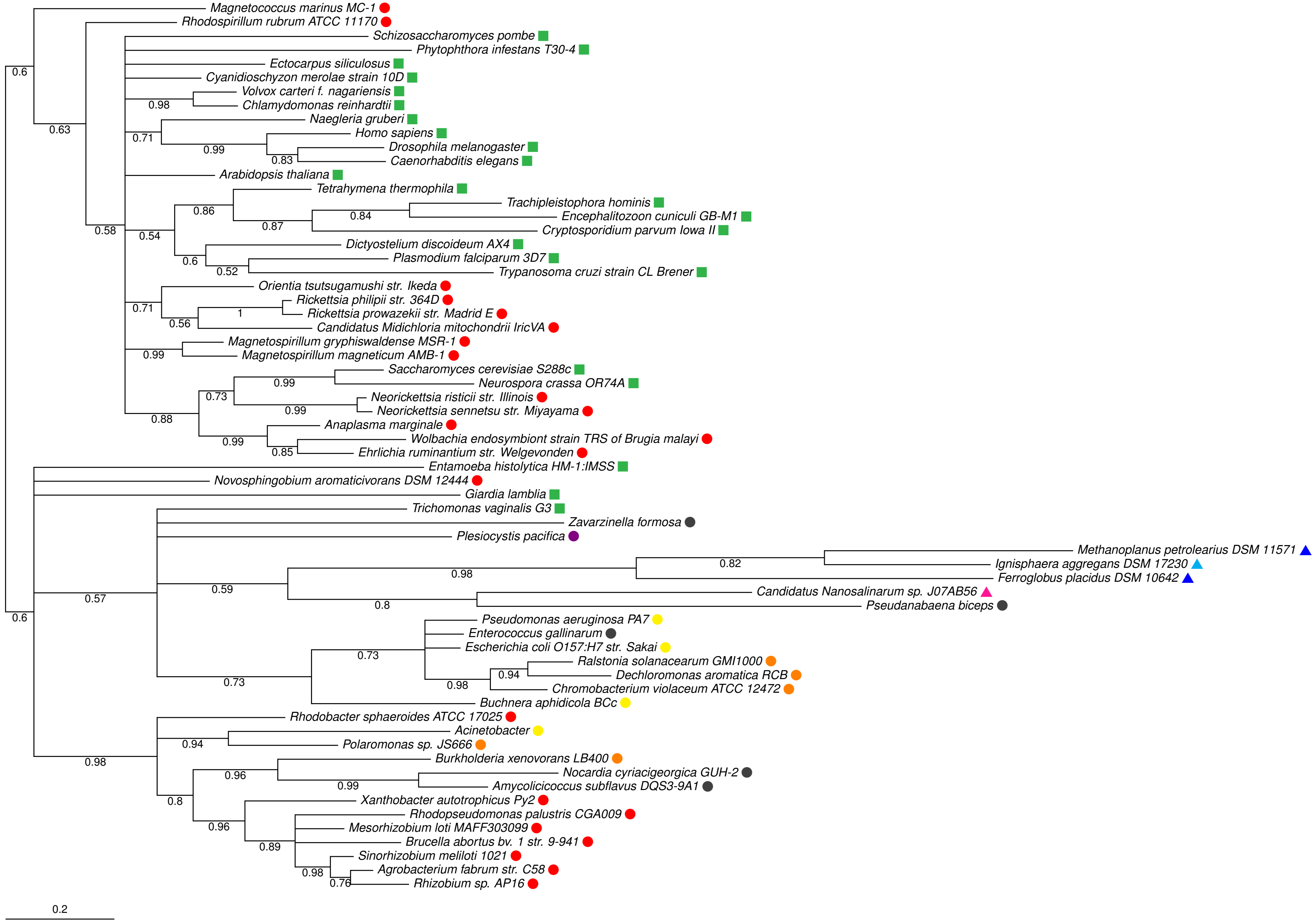

Magnetococcus marinus MC-1
Rhodospirillum rubrum ATCC 11170
Schizosaccharomyces pombe
Phytophthora infestans T30-4
Ectocarpus siliculosus
Cyanidioschyzon merolae strain 10D
Volvox carteri f. nagariensis
Chlamydomonas reinhardtii
Naegleria gruberi
Homo sapiens
Drosophila melanogaster
Caenorhabditis elegans
Arabidopsis thaliana
Tetrahymena thermophila
Trachipleistophora hominis
Encephalitozoon cuniculi GB-M1
Cryptosporidium parvum Iowa II
Dictyostelium discoideum AX4
Plasmodium falciparum 3D7
Trypanosoma cruzi strain CL Brener
Orientia tsutsugamushi str. Ikeda
Rickettsia philipii str. 364D
Rickettsia prowazekii str. Madrid E
Candidatus Midichloria mitochondrii IricVA
Magnetospirillum gryphiswaldense MSR-1
Magnetospirillum magneticum AMB-1
Saccharomyces cerevisiae S288c
Neurospora crassa OR74A
Neorickettsia risticii str. Illinois
Neorickettsia sennetsu str. Miyayama
Anaplasma marginale
Wolbachia endosymbiont strain TRS of Brugia malayi
Ehrlichia ruminantium str. Welgevonden
Entamoeba histolytica HM-1:IMSS
Novosphingobium aromaticivorans DSM 12444
Giardia lamblia
Trichomonas vaginalis G3
Zavarzinella formosa
Plesiocystis pacifica
Methanoplanus petrolearius DSM 11571
Ignisphaera aggregans DSM 17230
Ferroglobus placidus DSM 10642
Candidatus Nanosalinarum sp. J07AB56
Pseudanabaena biceps
Pseudomonas aeruginosa PA7
Enterococcus gallinarum
Escherichia coli O157:H7 str. Sakai
Ralstonia solanacearum GMI1000
Dechloromonas aromatica RCB
Chromobacterium violaceum ATCC 12472
Buchnera aphidicola BCc
Rhodobacter sphaeroides ATCC 17025
Acinetobacter
Polaromonas sp. JS666
Burkholderia xenovorans LB400
Nocardia cyriacigeorgica GUH-2
Amycolicicoccus subflavus DQS3-9A1
Xanthobacter autotrophicus Py2
Rhodopseudomonas palustris CGA009
Mesorhizobium loti MAFF303099
Brucella abortus bv. 1 str. 9-941
Sinorhizobium meliloti 1021
Agrobacterium fabrum str. C58
Rhizobium sp. AP16
0.6
0.63
0.98
0.71
0.99
0.83
0.58
0.54
0.86
0.87
0.84
0.6
0.52
0.71
0.56
1
0.99
0.73
0.99
0.99
0.88
0.99
0.85
0.6
0.57
0.59
0.98
0.82
0.8
0.73
0.73
0.94
0.98
0.98
0.94
0.96
0.99
0.8
0.96
0.89
0.98
0.76
0.2

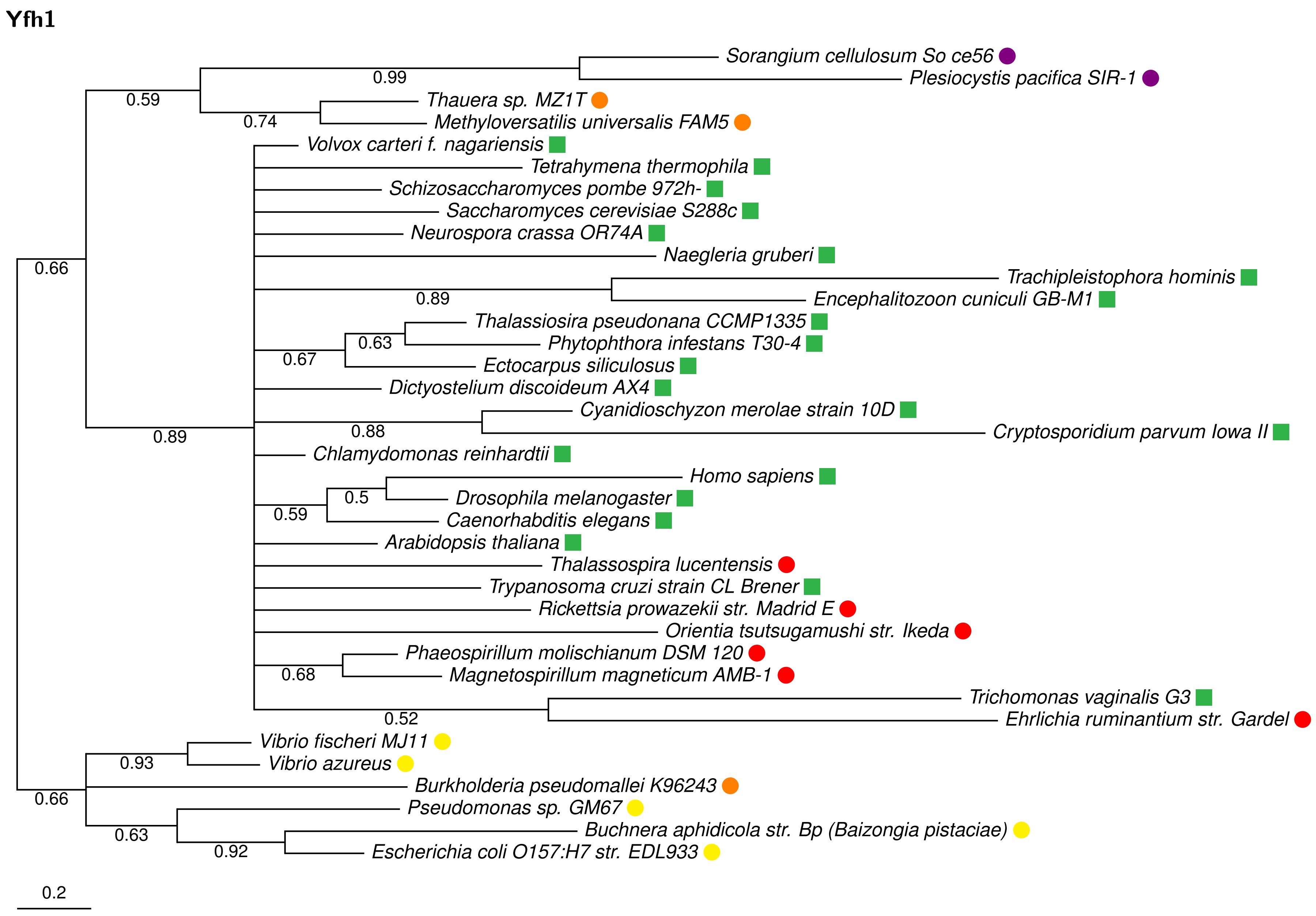

Yfh1
Sorangium cellulosum So ce56
Plesiocystis pacifica SIR-1
0.99
Thauera sp. MZ1T
0.59
Methyloversatilis universalis FAM5
0.74
Volvox carteri f. nagariensis
Tetrahymena thermophila
Schizosaccharomyces pombe 972h-
Saccharomyces cerevisiae S288c
Neurospora crassa OR74A
Naegleria gruberi
0.66
Trachipleistophora hominis
Encephalitozoon cuniculi GB-M1
0.89
Thalassiosira pseudonana CCMP1335
Phytophthora infestans T30-4
0.63
0.67
Ectocarpus siliculosus
Dictyostelium discoideum AX4
Cyanidioschyzon merolae strain 10D
Cryptosporidium parvum Iowa II
0.88
0.89
Chlamydomonas reinhardtii
Homo sapiens
Drosophila melanogaster
0.5
0.59
Caenorhabditis elegans
Arabidopsis thaliana
Thalassospira lucentensis
Trypanosoma cruzi strain CL Brener
Rickettsia prowazekii str. Madrid E
Orientia tsutsugamushi str. Ikeda
Phaeospirillum molischianum DSM 120
0.68
Magnetospirillum magneticum AMB-1
Trichomonas vaginalis G3
0.52
Ehrlichia ruminantium str. Gardel
Vibrio fischeri MJ11
0.93
Vibrio azureus
Burkholderia pseudomallei K96243
0.66
Pseudomonas sp. GM67
Buchnera aphidicola str. Bp (Baizongia pistaciae)
0.63
0.92
Escherichia coli O157:H7 str. EDL933
0.2

Nfs1

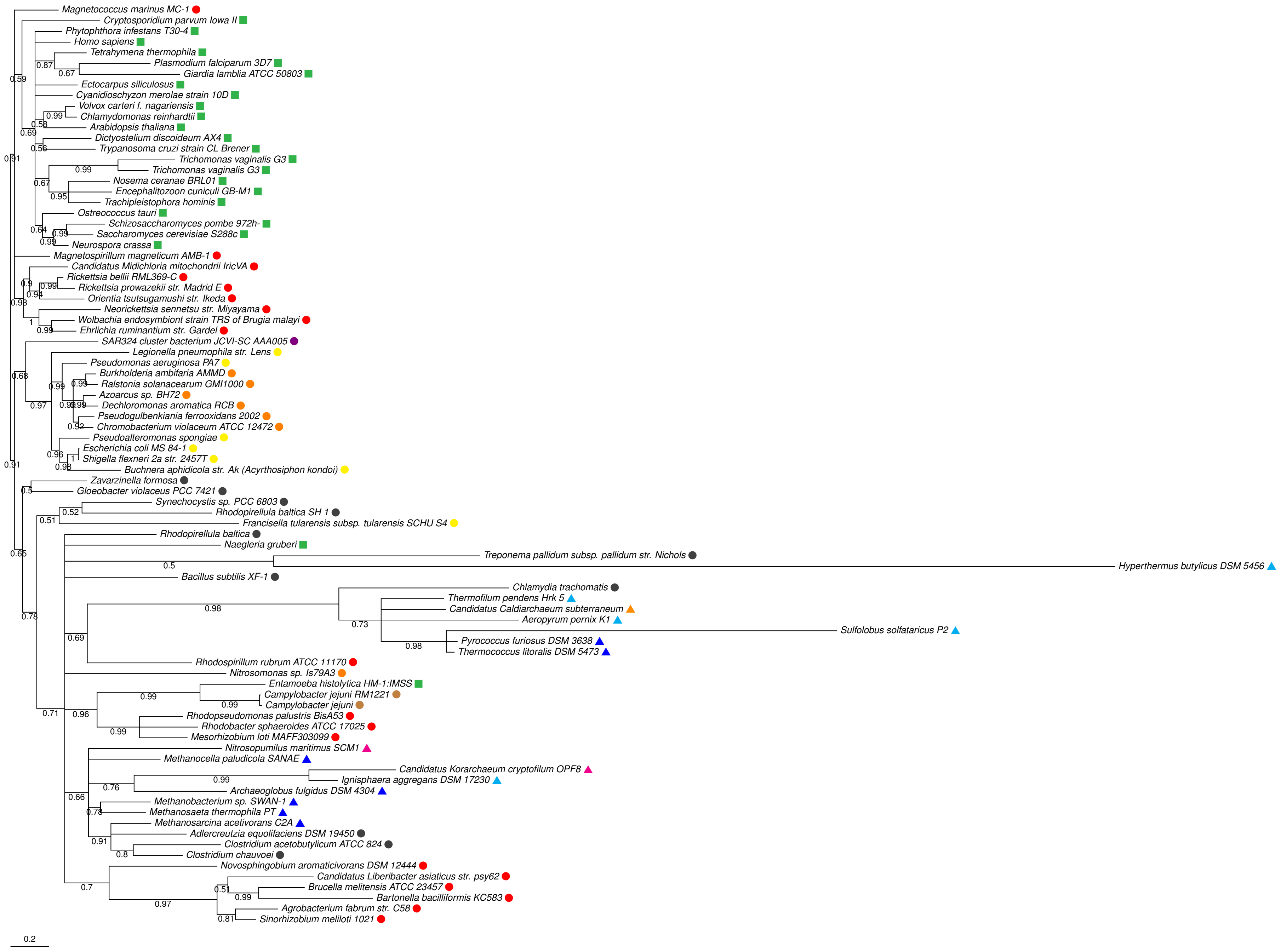
Magnetococcus marinus MC-1
Cryptosporidium parvum Iowa II
Phytophthora infestans T30-4
Homo sapiens
Tetrahymena thermophila
Plasmodium falciparum 3D7
Giardia lamblia ATCC 50803
Ectocarpus siliculosus
Cyanidioschyzon merolae strain 10D
Volvox carteri f. nagariensis
Chlamydomonas reinhardtii
Arabidopsis thaliana
Dictyostelium discoideum AX4
Trypanosoma cruzi strain CL Brener
Trichomonas vaginalis G3
Trichomonas vaginalis G3
Nosema ceranae BRL01
Encephalitozoon cuniculi GB-M1
Trachipleistophora hominis
Ostreococcus tauri
Schizosaccharomyces pombe 972h-
Saccharomyces cerevisiae S288c
Neurospora crassa
Magnetospirillum magneticum AMB-1
Candidatus Midichloria mitochondrii IricVA
Rickettsia bellii RML369-C
Rickettsia prowazekii str. Madrid E
Orientia tsutsugamushi str. Ikeda
Neorickettsia sennetsu str. Miyayama
Wolbachia endosymbiont strain TRS of Brugia malayi
Ehrlichia ruminantium str. Gardel
SAR324 cluster bacterium JCVI-SC AAA005
Legionella pneumophila str. Lens
Pseudomonas aeruginosa PA7
Burkholderia ambifaria AMMD
Ralstonia solanacearum GMI1000
Azoarcus sp. BH72
Dechloromonas aromatica RCB
Pseudogulbenkiania ferrooxidans 2002
Chromobacterium violaceum ATCC 12472
Pseudoalteromonas spongiae
Escherichia coli MS 84-1
Shigella flexneri 2a str. 2457T
Buchnera aphidicola str. Ak (Acyrthosiphon kondoi)
Zavarzinella formosa
Gloeobacter violaceus PCC 7421
Synechocystis sp. PCC 6803
Rhodopirellula baltica SH 1
Francisella tularensis subsp. tularensis SCHU S4
Rhodopirellula baltica
Naegleria gruberi
Treponema pallidum subsp. pallidum str. Nichols
Hyperthermus butylicus DSM 5456
Bacillus subtilis XF-1
Chlamydia trachomatis
Thermofilum pendens Hrk 5
Candidatus Caldiarchaeum subterraneum
Aeropyrum pernix K1
Sulfolobus solfataricus P2
Pyrococcus furiosus DSM 3638
Thermococcus litoralis DSM 5473
Rhodospirillum rubrum ATCC 11170
Nitrosomonas sp. Is79A3
Entamoeba histolytica HM-1:IMSS
Campylobacter jejuni RM1221
Campylobacter jejuni
Rhodopseudomonas palustris BisA53
Rhodobacter sphaeroides ATCC 17025
Mesorhizobium loti MAFF303099
Nitrosopumilus maritimus SCM1
Methanocella paludicola SANAE
Candidatus Korarchaeum cryptofilum OPF8
Ignisphaera aggregans DSM 17230
Archaeoglobus fulgidus DSM 4304
Methanobacterium sp. SWAN-1
Methanosaeta thermophila PT
Methanosarcina acetivorans C2A
Adlercreutzia equolifaciens DSM 19450
Clostridium acetobutylicum ATCC 824
Clostridium chauvoei
Novosphingobium aromaticivorans DSM 12444
Candidatus Liberibacter asiaticus str. psy62
Brucella melitensis ATCC 23457
Bartonella bacilliformis KC583
Agrobacterium fabrum str. C58
Sinorhizobium meliloti 1021
0.2

Isu1

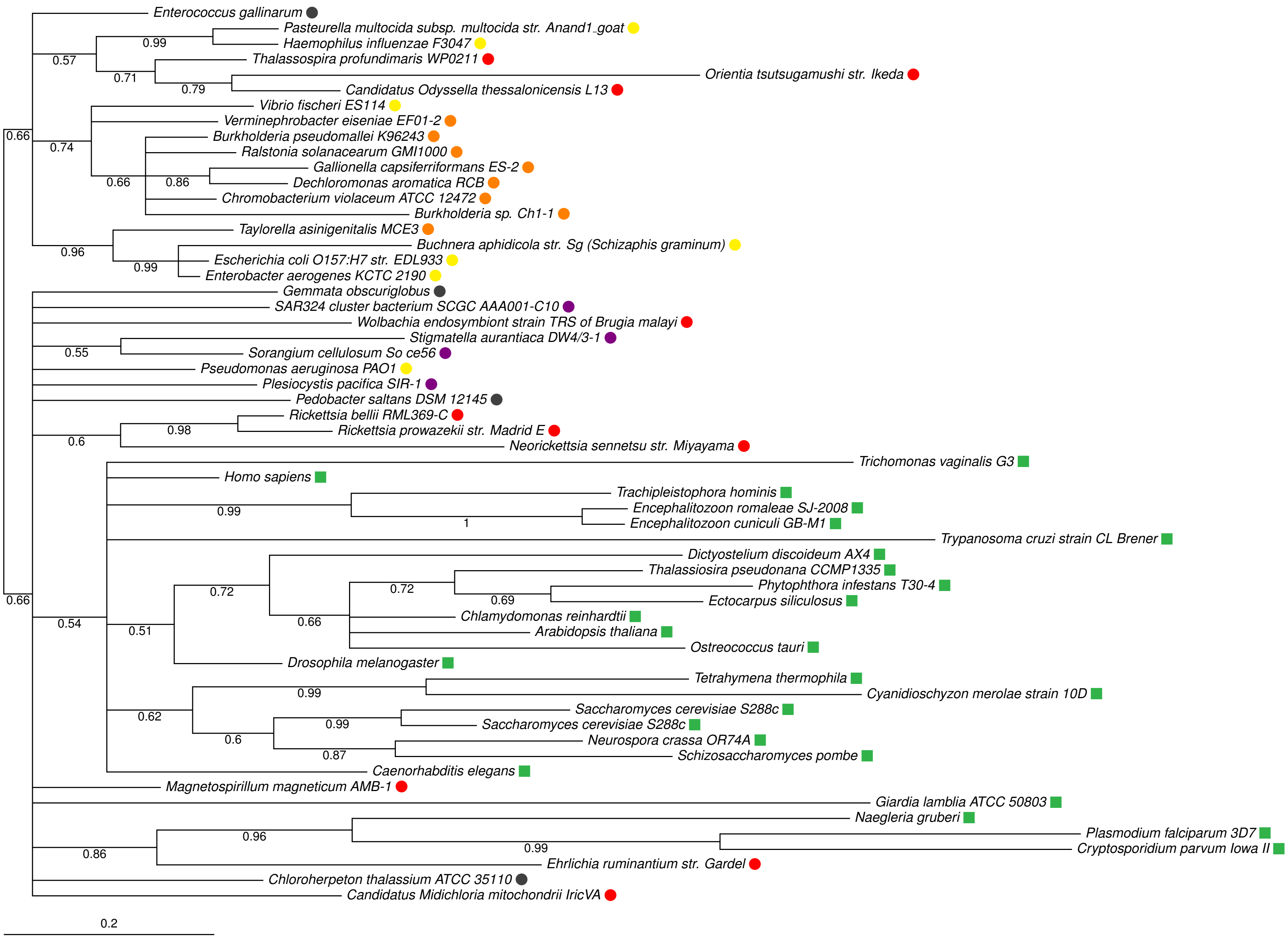
Enterococcus gallinarum
Pasteurella multocida subsp. multocida str. Anand1_goat
Haemophilus influenzae F3047
Thalassospira profundimaris WP0211
Orientia tsutsugamushi str. Ikeda
Candidatus Odyssella thessalonicensis L13
Vibrio fischeri ES114
Verminephrobacter eiseniae EF01-2
Burkholderia pseudomallei K96243
Ralstonia solanacearum GMI1000
Gallionella capsiferriformans ES-2
Dechloromonas aromatica RCB
Chromobacterium violaceum ATCC 12472
Burkholderia sp. Ch1-1
Taylorella asinigenitalis MCE3
Buchnera aphidicola str. Sg (Schizaphis graminum)
Escherichia coli O157:H7 str. EDL933
Enterobacter aerogenes KCTC 2190
Gemmata obscuriglobus
SAR324 cluster bacterium SCGC AAA001-C10
Wolbachia endosymbiont strain TRS of Brugia malayi
Stigmatella aurantiaca DW4/3-1
Sorangium cellulosum So ce56
Pseudomonas aeruginosa PAO1
Plesiocystis pacifica SIR-1
Pedobacter saltans DSM 12145
Rickettsia bellii RML369-C
Rickettsia prowazekii str. Madrid E
Neorickettsia sennetsu str. Miyayama
Trichomonas vaginalis G3
Homo sapiens
Trachipleistophora hominis
Encephalitozoon romaleae SJ-2008
Encephalitozoon cuniculi GB-M1
Trypanosoma cruzi strain CL Brener
Dictyostelium discoideum AX4
Thalassiosira pseudonana CCMP1335
Phytophthora infestans T30-4
Ectocarpus siliculosus
Chlamydomonas reinhardtii
Arabidopsis thaliana
Ostreococcus tauri
Drosophila melanogaster
Tetrahymena thermophila
Cyanidioschyzon merolae strain 10D
Saccharomyces cerevisiae S288c
Saccharomyces cerevisiae S288c
Neurospora crassa OR74A
Schizosaccharomyces pombe
Caenorhabditis elegans
Magnetospirillum magneticum AMB-1
Giardia lamblia ATCC 50803
Naegleria gruberi
Plasmodium falciparum 3D7
Cryptosporidium parvum Iowa II
Ehrlichia ruminantium str. Gardel
Chloroherpeton thalassium ATCC 35110
Candidatus Midichloria mitochondrii IricVA
0.57
0.99
0.71
0.79
0.66
0.74
0.66
0.86
0.96
0.99
0.55
0.6
0.98
0.99
1
0.72
0.72
0.69
0.66
0.66
0.54
0.51
0.99
0.62
0.99
0.6
0.87
0.96
0.99
0.86
0.2

Grx proteins

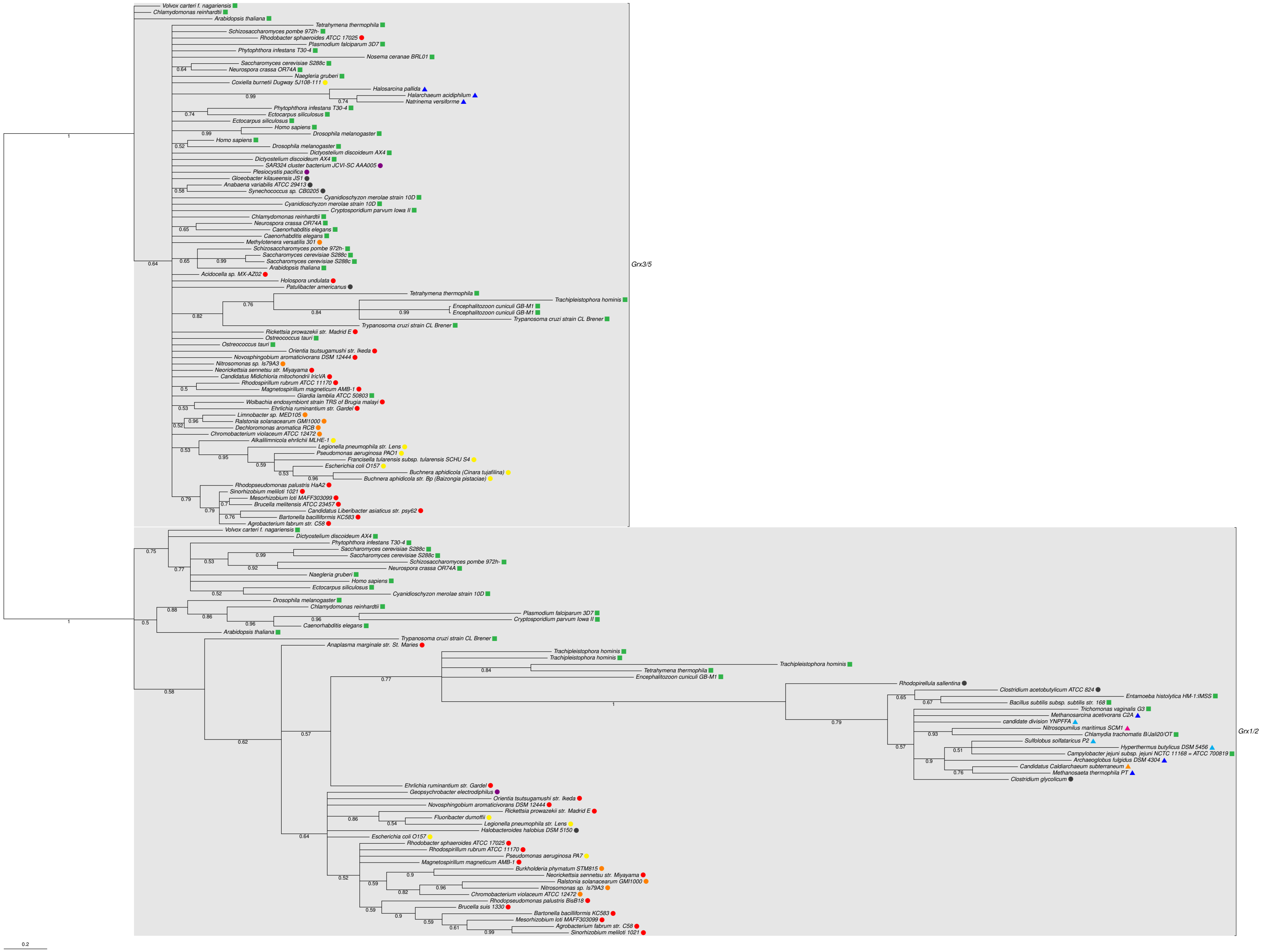

Atm1

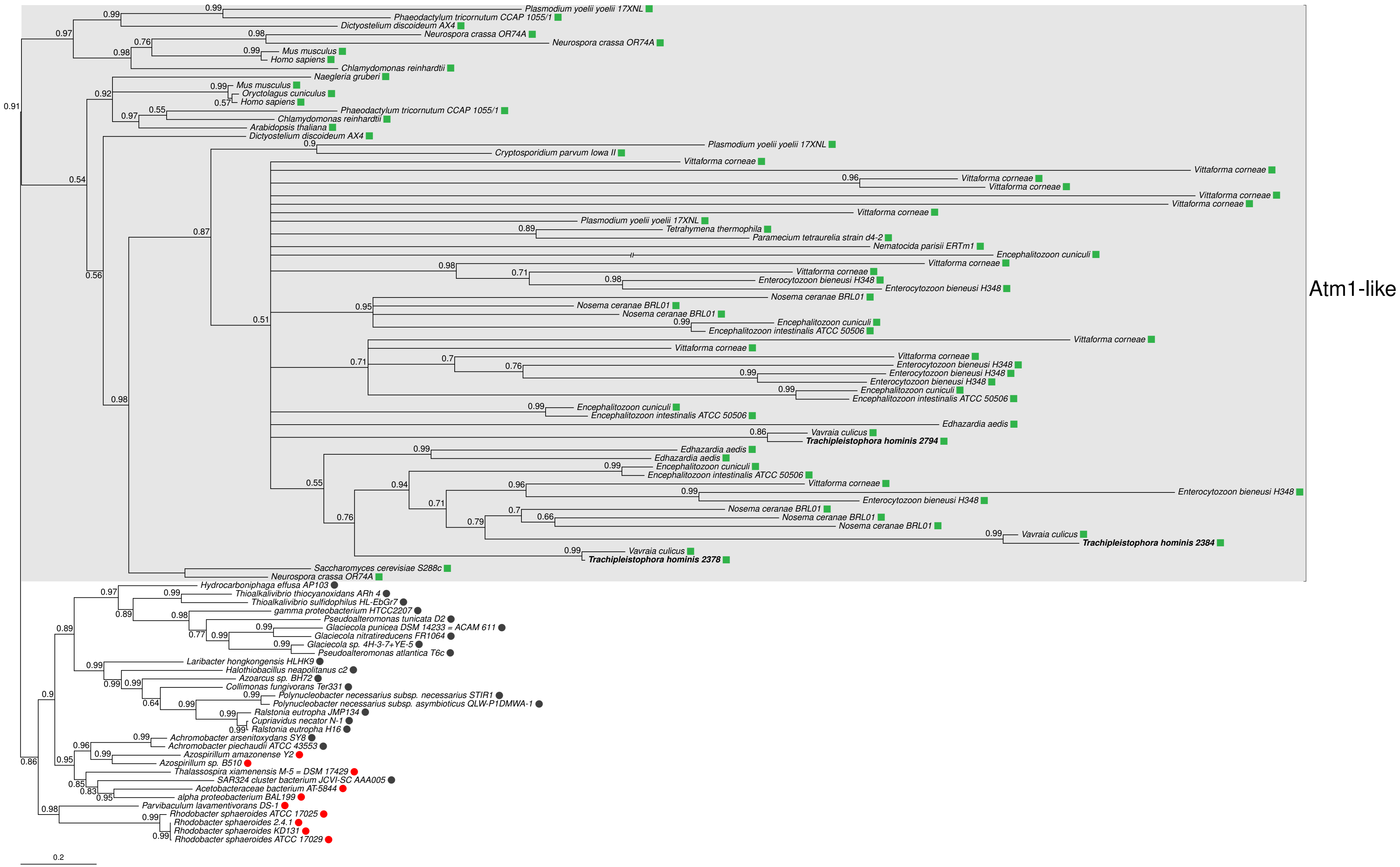

Atm1-like
Plasmodium yoelii yoelii 17XNL
Phaeodactylum tricornutum CCAP 1055/1
Dictyostelium discoideum AX4
Neurospora crassa OR74A
Neurospora crassa OR74A
Mus musculus
Homo sapiens
Chlamydomonas reinhardtii
Naegleria gruberi
Mus musculus
Oryctolagus cuniculus
Homo sapiens
Phaeodactylum tricornutum CCAP 1055/1
Chlamydomonas reinhardtii
Arabidopsis thaliana
Dictyostelium discoideum AX4
Plasmodium yoelii yoelii 17XNL
Cryptosporidium parvum Iowa II
Vittaforma corneae
Vittaforma corneae
Vittaforma corneae
Vittaforma corneae
Vittaforma corneae
Vittaforma corneae
Vittaforma corneae
Plasmodium yoelii yoelii 17XNL
Tetrahymena thermophila
Paramecium tetraurelia strain d4-2
Nematocida parisii ERTm1
Encephalitozoon cuniculi
Vittaforma corneae
Vittaforma corneae
Enterocytozoon bieneusi H348
Enterocytozoon bieneusi H348
Nosema ceranae BRL01
Nosema ceranae BRL01
Nosema ceranae BRL01
Encephalitozoon cuniculi
Encephalitozoon intestinalis ATCC 50506
Vittaforma corneae
Vittaforma corneae
Vittaforma corneae
Enterocytozoon bieneusi H348
Enterocytozoon bieneusi H348
Enterocytozoon bieneusi H348
Encephalitozoon cuniculi
Encephalitozoon intestinalis ATCC 50506
Encephalitozoon cuniculi
Encephalitozoon intestinalis ATCC 50506
Edhazardia aedis
Vavraia culicus
Trachipleistophora hominis 2794
Edhazardia aedis
Edhazardia aedis
Encephalitozoon cuniculi
Encephalitozoon intestinalis ATCC 50506
Vittaforma corneae
Enterocytozoon bieneusi H348
Enterocytozoon bieneusi H348
Nosema ceranae BRL01
Nosema ceranae BRL01
Nosema ceranae BRL01
Vavraia culicus
Trachipleistophora hominis 2384
Vavraia culicus
Trachipleistophora hominis 2378
Saccharomyces cerevisiae S288c
Neurospora crassa OR74A
Hydrocarboniphaga effusa AP103
Thioalkalivibrio thiocyanoxidans ARh 4
Thioalkalivibrio sulfidophilus HL-EbGr7
gamma proteobacterium HTCC2207
Pseudoalteromonas tunicata D2
Glaciecola punicea DSM 14233 = ACAM 611
Glaciecola nitratireducens FR1064
Glaciecola sp. 4H-3-7+YE-5
Pseudoalteromonas atlantica T6c
Laribacter hongkongensis HLHK9
Halothiobacillus neapolitanus c2
Azoarcus sp. BH72
Collimonas fungivorans Ter331
Polynucleobacter necessarius subsp. necessarius STIR1
Polynucleobacter necessarius subsp. asymbioticus QLW-P1DMWA-1
Ralstonia eutropha JMP134
Cupriavidus necator N-1
Ralstonia eutropha H16
Achromobacter arsenitoxydans SY8
Achromobacter piechaudii ATCC 43553
Azospirillum amazonense Y2
Azospirillum sp. B510
Thalassospira xiamenensis M-5 = DSM 17429
SAR324 cluster bacterium JCVI-SC AAA005
Acetobacteraceae bacterium AT-5844
alpha proteobacterium BAL199
Parvibaculum lavamentivorans DS-1
Rhodobacter sphaeroides ATCC 17025
Rhodobacter sphaeroides 2.4.1
Rhodobacter sphaeroides KD131
Rhodobacter sphaeroides ATCC 17029
0.2

2 Mitochondrial / mitosomal Fe/S cluster (ISC) assembly components lost in the Microsporidia

Iba57

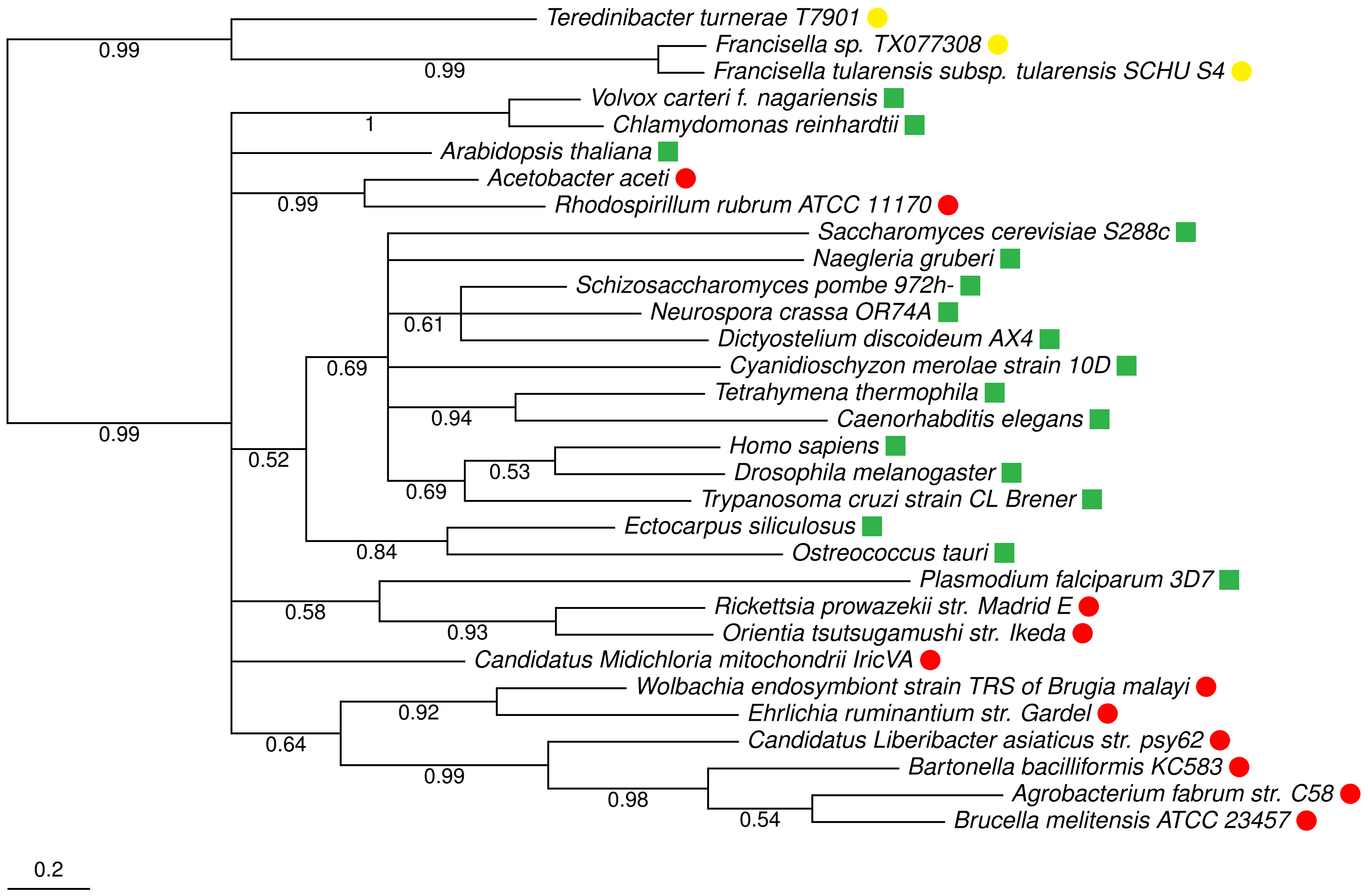

Isa1/2

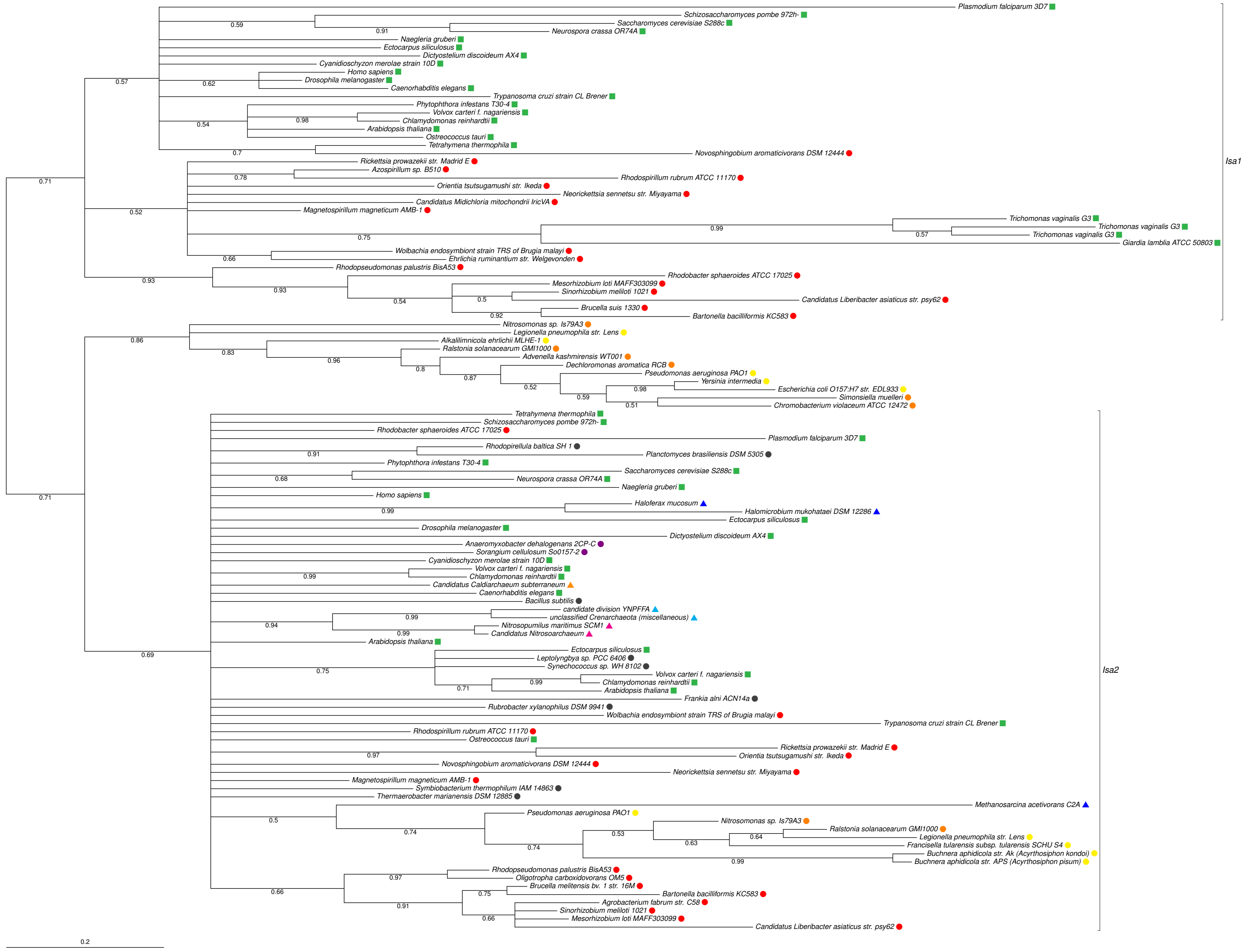
Plasmodium falciparum 3D7
Schizosaccharomyces pombe 972h-
0.59
Saccharomyces cerevisiae S288c
0.91
Neurospora crassa OR74A
Naegleria gruberi
Ectocarpus siliculosus
Dictyostelium discoideum AX4
Cyanidioschyzon merolae strain 10D
Homo sapiens
0.57
0.62
Drosophila melanogaster
Caenorhabditis elegans
Trypanosoma cruzi strain CL Brener
Phytophthora infestans T30-4
Volvox carteri f. nagariensis
0.98
Chlamydomonas reinhardtii
0.54
Arabidopsis thaliana
Ostreococcus tauri
Tetrahymena thermophila
0.7
Novosphingobium aromaticivorans DSM 12444
Rickettsia prowazekii str. Madrid E
Azospirillum sp. B510
0.71
0.78
Rhodospirillum rubrum ATCC 11170
Orientia tsutsugamushi str. Ikeda
Neorickettsia sennetsu str. Miyayama
Candidatus Midichloria mitochondrii IricVA
0.52
Magnetospirillum magneticum AMB-1
Trichomonas vaginalis G3
0.99
Trichomonas vaginalis G3
0.75
0.57
Trichomonas vaginalis G3
Giardia lamblia ATCC 50803
Wolbachia endosymbiont strain TRS of Brugia malayi
0.66
Ehrlichia ruminantium str. Welgevonden
Rhodopseudomonas palustris BisA53
0.93
Rhodobacter sphaeroides ATCC 17025
Mesorhizobium loti MAFF303099
0.93
Sinorhizobium meliloti 1021
0.5
0.54
Candidatus Liberibacter asiaticus str. psy62
Brucella suis 1330
0.92
Bartonella bacilliformis KC583
Isa1
Nitrosomonas sp. Is79A3
Legionella pneumophila str. Lens
0.86
Alkalilimnicola ehrlichii MLHE-1
0.83
Ralstonia solanacearum GMI1000
0.96
Advenella kashmirensis WT001
0.8
Dechloromonas aromatica RCB
0.87
Pseudomonas aeruginosa PAO1
Yersinia intermedia
0.52
0.98
Escherichia coli O157:H7 str. EDL933
0.59
Simonsiella muelleri
0.51
Chromobacterium violaceum ATCC 12472
Tetrahymena thermophila
Schizosaccharomyces pombe 972h-
Rhodobacter sphaeroides ATCC 17025
Plasmodium falciparum 3D7
Rhodopirellula baltica SH 1
0.91
Planctomyces brasiliensis DSM 5305
Phytophthora infestans T30-4
Saccharomyces cerevisiae S288c
0.68
Neurospora crassa OR74A
Naegleria gruberi
0.71
Homo sapiens
Haloferax mucosum
0.99
Halomicrobium mukohataei DSM 12286
Ectocarpus siliculosus
Drosophila melanogaster
Dictyostelium discoideum AX4
Anaeromyxobacter dehalogenans 2CP-C
Sorangium cellulosum So0157-2
Cyanidioschyzon merolae strain 10D
Volvox carteri f. nagariensis
0.99
Chlamydomonas reinhardtii
Candidatus Caldiarchaeum subterraneum
Caenorhabditis elegans
Bacillus subtilis
candidate division YNPFFA
0.99
unclassified Crenarchaeota (miscellaneous)
0.94
Nitrosopumilus maritimus SCM1
0.99
Candidatus Nitrosoarchaeum
Arabidopsis thaliana
0.69
Ectocarpus siliculosus
Leptolyngbya sp. PCC 6406
0.75
Synechococcus sp. WH 8102
Volvox carteri f. nagariensis
0.99
Chlamydomonas reinhardtii
0.71
Arabidopsis thaliana
Frankia alni ACN14a
Rubrobacter xylanophilus DSM 9941
Wolbachia endosymbiont strain TRS of Brugia malayi
Trypanosoma cruzi strain CL Brener
Rhodospirillum rubrum ATCC 11170
Ostreococcus tauri
Rickettsia prowazekii str. Madrid E
0.97
Orientia tsutsugamushi str. Ikeda
Novosphingobium aromaticivorans DSM 12444
Neorickettsia sennetsu str. Miyayama
Magnetospirillum magneticum AMB-1
Symbiobacterium thermophilum IAM 14863
Thermaerobacter marianensis DSM 12885
Methanosarcina acetivorans C2A
0.5
Pseudomonas aeruginosa PAO1
0.74
Nitrosomonas sp. Is79A3
0.53
Ralstonia solanacearum GMI1000
0.74
0.63
0.64
Legionella pneumophila str. Lens
Francisella tularensis subsp. tularensis SCHU S4
0.99
Buchnera aphidicola str. Ak (Acyrthosiphon kondoi)
Buchnera aphidicola str. APS (Acyrthosiphon pisum)
Rhodopseudomonas palustris BisA53
0.97
Oligotropha carboxidovorans OM5
Brucella melitensis bv. 1 str. 16M
0.66
0.75
Bartonella bacilliformis KC583
0.91
Agrobacterium fabrum str. C58
Sinorhizobium meliloti 1021
0.66
Mesorhizobium loti MAFF303099
Candidatus Liberibacter asiaticus str. psy62
Isa2
0.2

Mge1

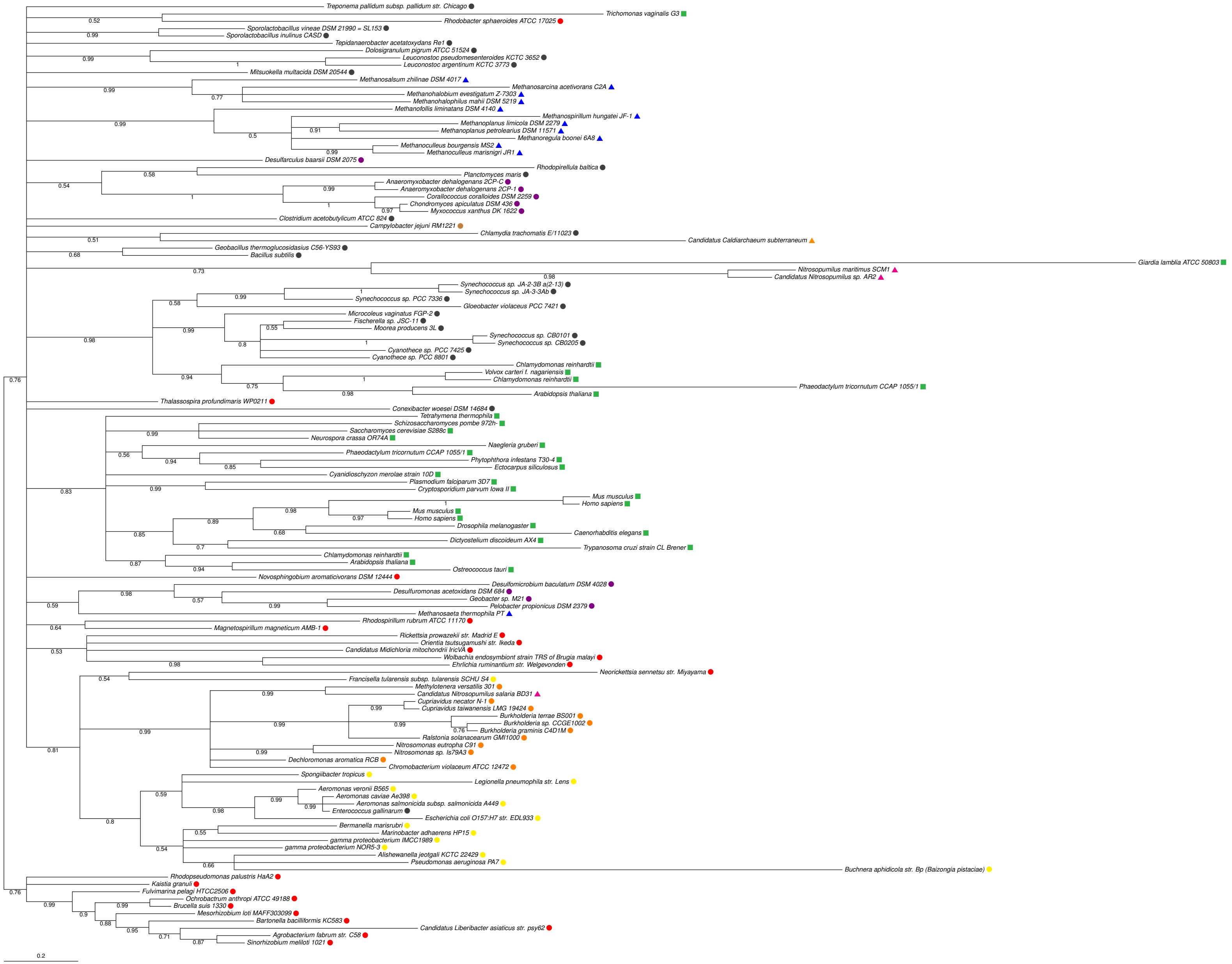
Treponema pallidum subsp. pallidum str. Chicago
Trichomonas vaginalis G3
Rhodobacter sphaeroides ATCC 17025
Sporolactobacillus vineae DSM 21990 = SL153
Sporolactobacillus inulinus CASD
Tepidanaerobacter acetatoxydans Re1
Dolosigranulum pigrum ATCC 51524
Leuconostoc pseudomesenteroides KCTC 3652
Leuconostoc argentinum KCTC 3773
Mitsuokella multacida DSM 20544
Methanosalsum zhilinae DSM 4017
Methanosarcina acetivorans C2A
Methanohalobium evestigatum Z-7303
Methanohalophilus mahii DSM 5219
Methanofollis liminatans DSM 4140
Methanospirillum hungatei JF-1
Methanoplanus limicola DSM 2279
Methanoplanus petrolearius DSM 11571
Methanoregula boonei 6A8
Methanoculleus bourgensis MS2
Methanoculleus marisnigri JR1
Desulfarculus baarsii DSM 2075
Rhodopirellula baltica
Planctomyces maris
Anaeromyxobacter dehalogenans 2CP-C
Anaeromyxobacter dehalogenans 2CP-1
Corallococcus coralloides DSM 2259
Chondromyces apiculatus DSM 436
Myxococcus xanthus DK 1622
Clostridium acetobutylicum ATCC 824
Campylobacter jejuni RM1221
Chlamydia trachomatis E/11023
Candidatus Caldiarchaeum subterraneum
Geobacillus thermoglucosidasius C56-YS93
Bacillus subtilis
Giardia lamblia ATCC 50803
Nitrosopumilus maritimus SCM1
Candidatus Nitrosopumilus sp. AR2
Synechococcus sp. JA-2-3B a(2-13)
Synechococcus sp. JA-3-3Ab
Synechococcus sp. PCC 7336
Gloeobacter violaceus PCC 7421
Microcoleus vaginatus FGP-2
Fischerella sp. JSC-11
Moorea producens 3L
Synechococcus sp. CB0101
Synechococcus sp. CB0205
Cyanothece sp. PCC 7425
Cyanothece sp. PCC 8801
Chlamydomonas reinhardtii
Volvox carteri f. nagariensis
Chlamydomonas reinhardtii
Phaeodactylum tricornutum CCAP 1055/1
Arabidopsis thaliana
Thalassospira profundimaris WP0211
Conexibacter woesei DSM 14684
Tetrahymena thermophila
Schizosaccharomyces pombe 972h-
Saccharomyces cerevisiae S288c
Neurospora crassa OR74A
Naegleria gruberi
Phaeodactylum tricornutum CCAP 1055/1
Phytophthora infestans T30-4
Ectocarpus siliculosus
Cyanidioschyzon merolae strain 10D
Plasmodium falciparum 3D7
Cryptosporidium parvum Iowa II
Mus musculus
Homo sapiens
Mus musculus
Homo sapiens
Drosophila melanogaster
Caenorhabditis elegans
Dictyostelium discoideum AX4
Trypanosoma cruzi strain CL Brener
Chlamydomonas reinhardtii
Arabidopsis thaliana
Ostreococcus tauri
Novosphingobium aromaticivorans DSM 12444
Desulfomicrobium baculatum DSM 4028
Desulfuromonas acetoxidans DSM 684
Geobacter sp. M21
Pelobacter propionicus DSM 2379
Methanosaeta thermophila PT
Rhodospirillum rubrum ATCC 11170
Magnetospirillum magneticum AMB-1
Rickettsia prowazekii str. Madrid E
Orientia tsutsugamushi str. Ikeda
Candidatus Midichloria mitochondrii IricVA
Wolbachia endosymbiont strain TRS of Brugia malayi
Ehrlichia ruminantium str. Welgevonden
Neorickettsia sennetsu str. Miyayama
Francisella tularensis subsp. tularensis SCHU S4
Methylotenera versatilis 301
Candidatus Nitrosopumilus salaria BD31
Cupriavidus necator N-1
Cupriavidus taiwanensis LMG 19424
Burkholderia terrae BS001
Burkholderia sp. CCGE1002
Burkholderia graminis C4D1M
Ralstonia solanacearum GMI1000
Nitrosomonas eutropha C91
Nitrosomonas sp. Is79A3
Dechloromonas aromatica RCB
Chromobacterium violaceum ATCC 12472
Spongiibacter tropicus
Legionella pneumophila str. Lens
Aeromonas veronii B565
Aeromonas caviae Ae398
Aeromonas salmonicida subsp. salmonicida A449
Enterococcus gallinarum
Escherichia coli O157:H7 str. EDL933
Bermanella marisrubri
Marinobacter adhaerens HP15
gamma proteobacterium IMCC1989
gamma proteobacterium NOR5-3
Alishewanella jeotgali KCTC 22429
Pseudomonas aeruginosa PA7
Buchnera aphidicola str. Bp (Baizongia pistaciae)
Rhodopseudomonas palustris HaA2
Kaistia granuli
Fulvimarina pelagi HTCC2506
Ochrobactrum anthropi ATCC 49188
Brucella suis 1330
Mesorhizobium loti MAFF303099
Bartonella bacilliformis KC583
Candidatus Liberibacter asiaticus str. psy62
Agrobacterium fabrum str. C58
Sinorhizobium meliloti 1021
0.2

Nfu1

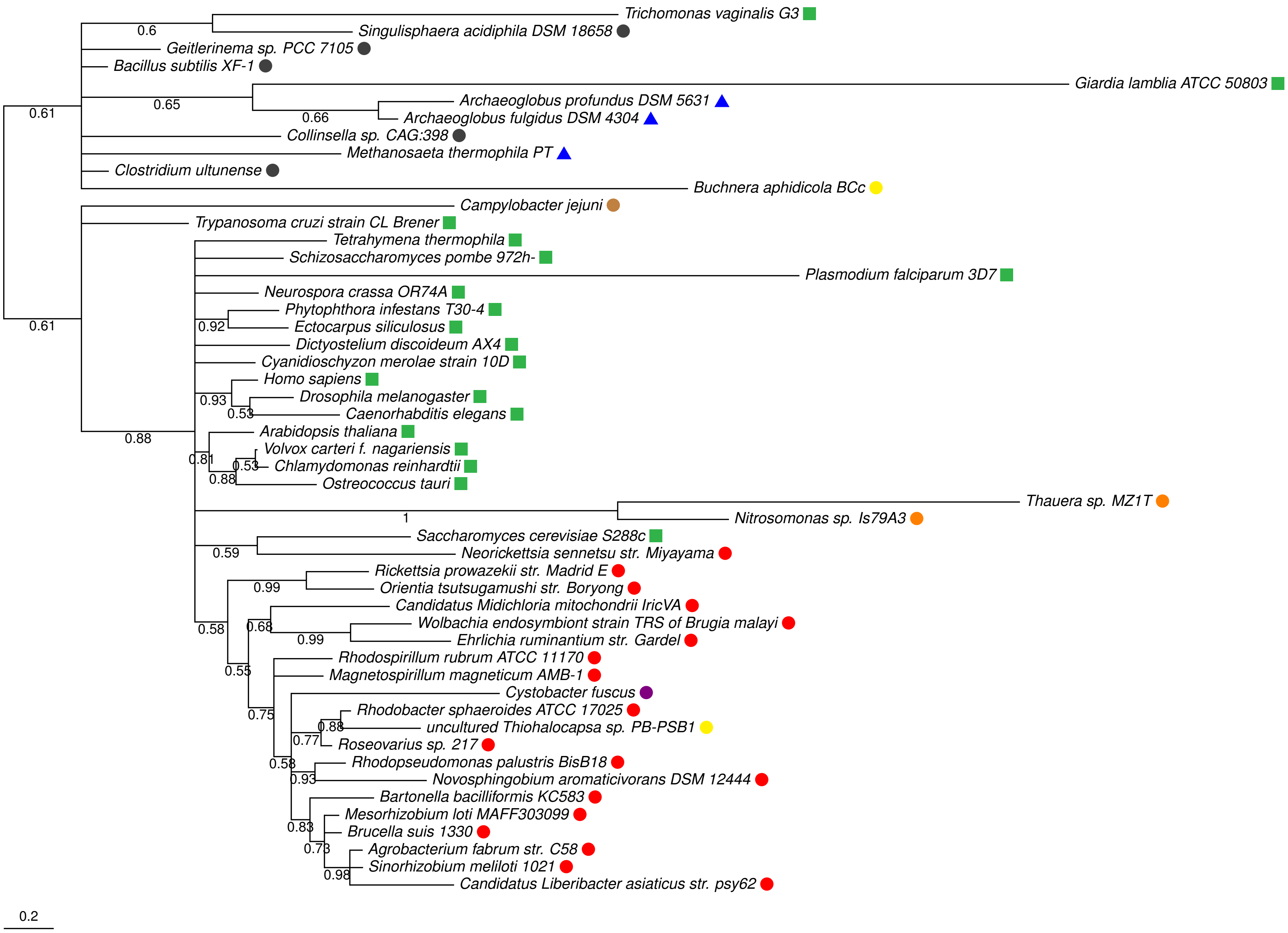
Trichomonas vaginalis G3
Singulisphaera acidiphila DSM 18658
Geitlerinema sp. PCC 7105
Bacillus subtilis XF-1
Giardia lamblia ATCC 50803
Archaeoglobus profundus DSM 5631
Archaeoglobus fulgidus DSM 4304
Collinsella sp. CAG:398
Methanosaeta thermophila PT
Clostridium ultunense
Buchnera aphidicola BCc
Campylobacter jejuni
Trypanosoma cruzi strain CL Brener
Tetrahymena thermophila
Schizosaccharomyces pombe 972h-
Plasmodium falciparum 3D7
Neurospora crassa OR74A
Phytophthora infestans T30-4
Ectocarpus siliculosus
Dictyostelium discoideum AX4
Cyanidioschyzon merolae strain 10D
Homo sapiens
Drosophila melanogaster
Caenorhabditis elegans
Arabidopsis thaliana
Volvox carteri f. nagariensis
Chlamydomonas reinhardtii
Ostreococcus tauri
Thauera sp. MZ1T
Nitrosomonas sp. Is79A3
Saccharomyces cerevisiae S288c
Neorickettsia sennetsu str. Miyayama
Rickettsia prowazekii str. Madrid E
Orientia tsutsugamushi str. Boryong
Candidatus Midichloria mitochondrii IricVA
Wolbachia endosymbiont strain TRS of Brugia malayi
Ehrlichia ruminantium str. Gardel
Rhodospirillum rubrum ATCC 11170
Magnetospirillum magneticum AMB-1
Cystobacter fuscus
Rhodobacter sphaeroides ATCC 17025
uncultured Thiohalocapsa sp. PB-PSB1
Roseovarius sp. 217
Rhodopseudomonas palustris BisB18
Novosphingobium aromaticivorans DSM 12444
Bartonella bacilliformis KC583
Mesorhizobium loti MAFF303099
Brucella suis 1330
Agrobacterium fabrum str. C58
Sinorhizobium meliloti 1021
Candidatus Liberibacter asiaticus str. psy62
0.6
0.65
0.66
0.61
0.61
0.92
0.93
0.53
0.88
0.81
0.53
0.88
1
0.59
0.99
0.58
0.68
0.99
0.55
0.75
0.88
0.77
0.58
0.93
0.83
0.73
0.98
0.2

3 Components of the cytosolic Fe/S protein assembly (CIA) pathway

Tah18

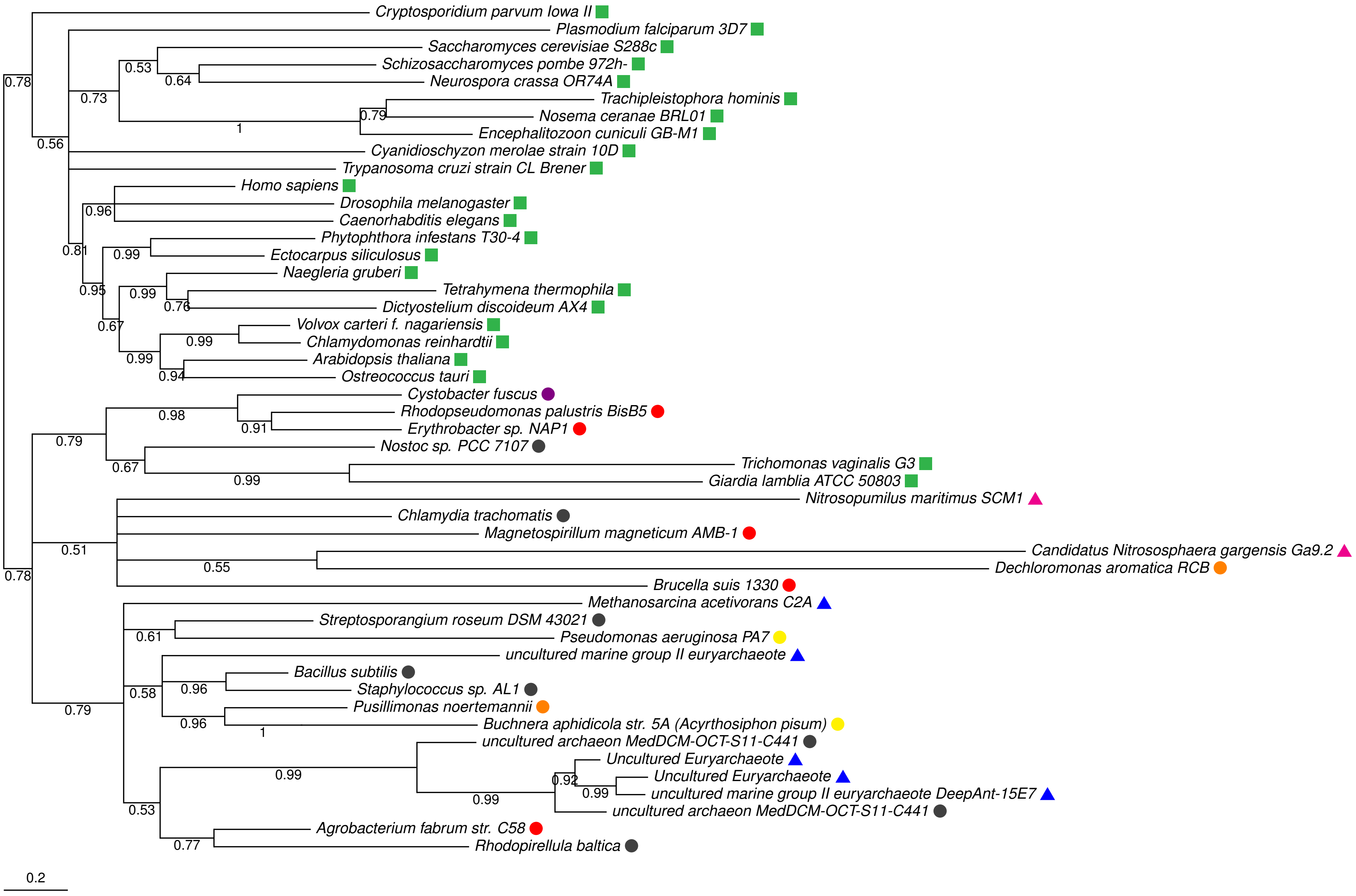

Cfd1/Nbp35/Ind1

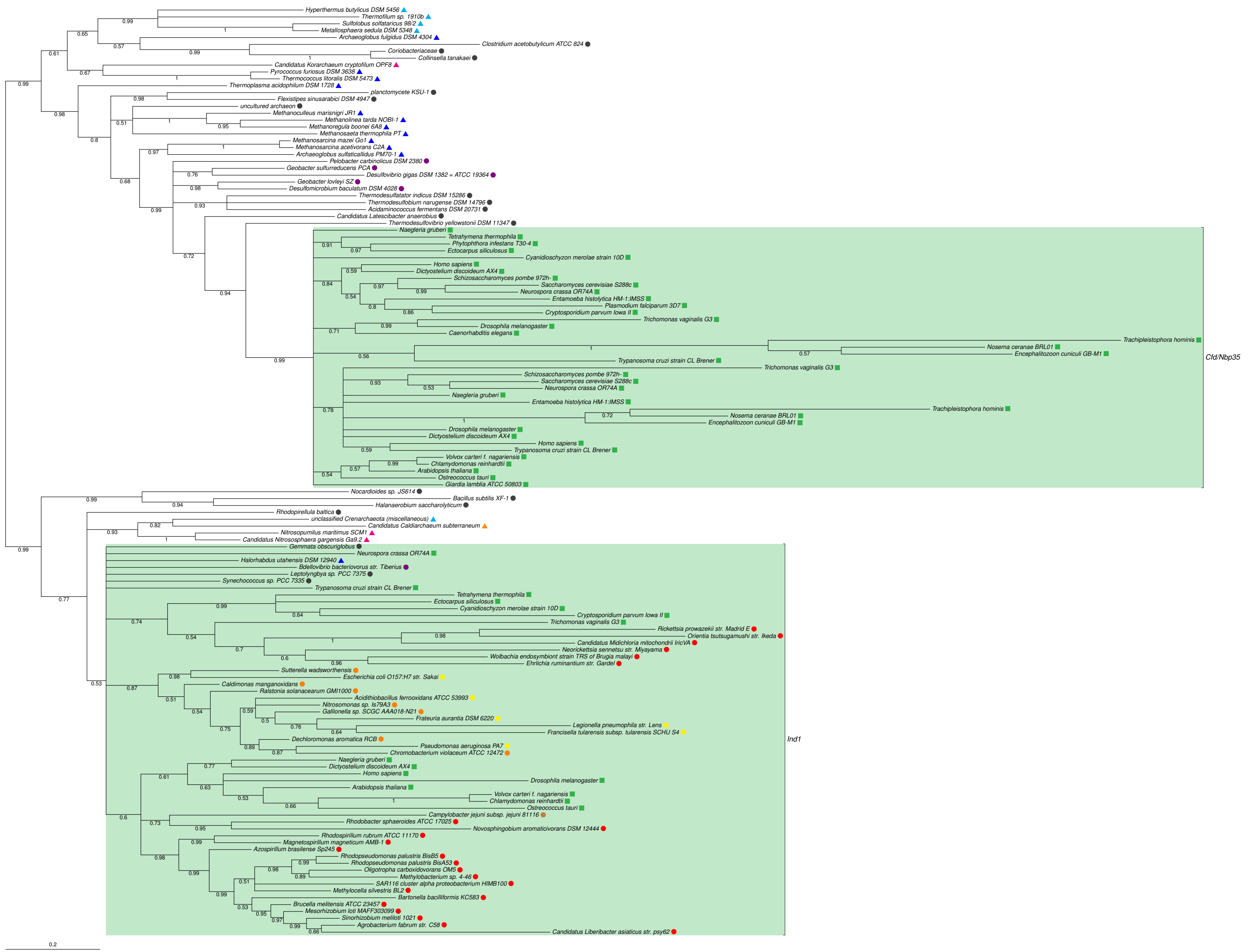

Ind1 is localized to mitochondria.

Nar1

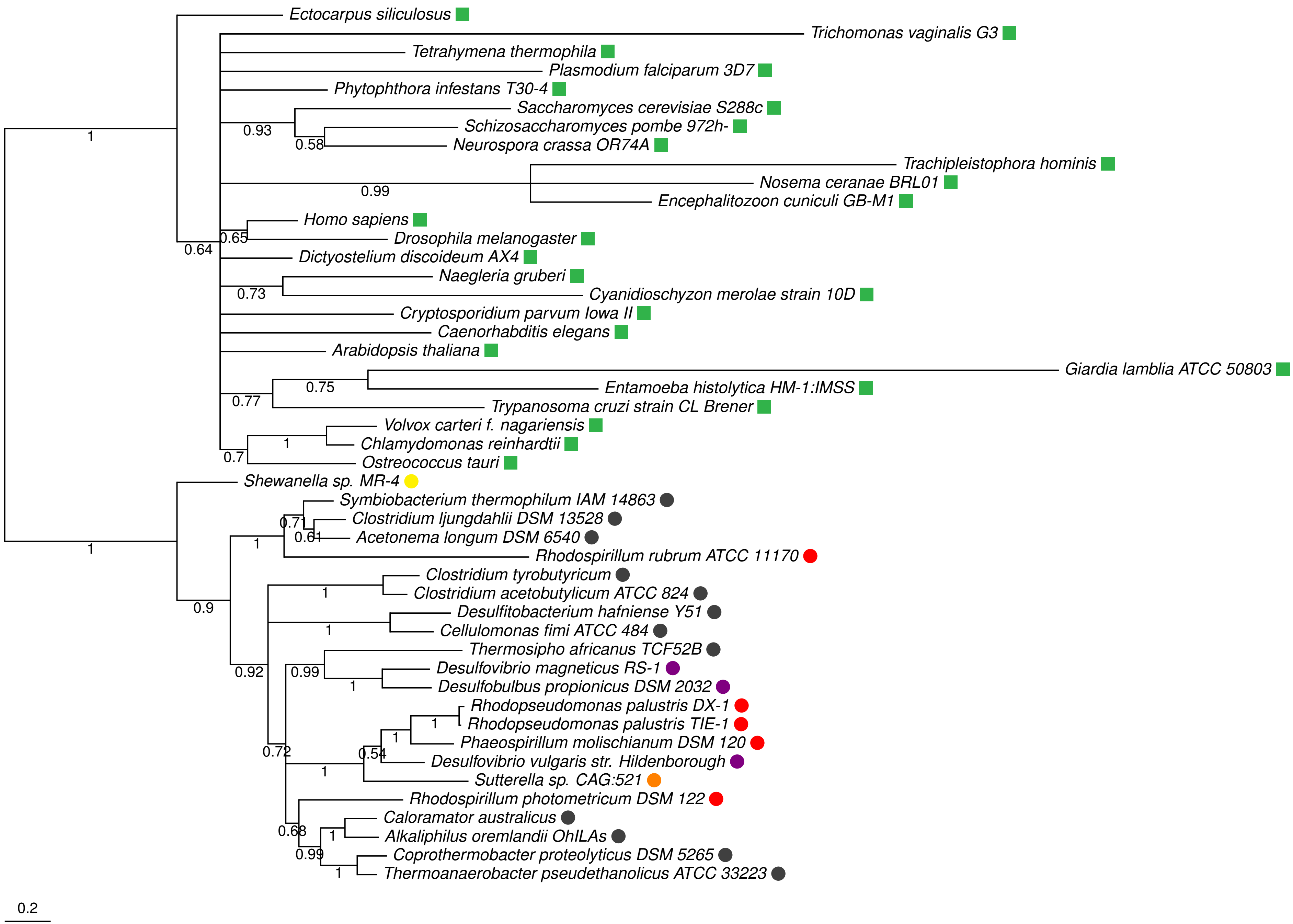
Ectocarpus siliculosus
Trichomonas vaginalis G3
Tetrahymena thermophila
Plasmodium falciparum 3D7
Phytophthora infestans T30-4
Saccharomyces cerevisiae S288c
Schizosaccharomyces pombe 972h-
Neurospora crassa OR74A
Trachipleistophora hominis
Nosema ceranae BRL01
Encephalitozoon cuniculi GB-M1
Homo sapiens
Drosophila melanogaster
Dictyostelium discoideum AX4
Naegleria gruberi
Cyanidioschyzon merolae strain 10D
Cryptosporidium parvum Iowa II
Caenorhabditis elegans
Arabidopsis thaliana
Giardia lamblia ATCC 50803
Entamoeba histolytica HM-1:IMSS
Trypanosoma cruzi strain CL Brener
Volvox carteri f. nagariensis
Chlamydomonas reinhardtii
Ostreococcus tauri
Shewanella sp. MR-4
Symbiobacterium thermophilum IAM 14863
Clostridium ljungdahlii DSM 13528
Acetonema longum DSM 6540
Rhodospirillum rubrum ATCC 11170
Clostridium tyrobutyricum
Clostridium acetobutylicum ATCC 824
Desulfitobacterium hafniense Y51
Cellulomonas fimi ATCC 484
Thermosipho africanus TCF52B
Desulfovibrio magneticus RS-1
Desulfobulbus propionicus DSM 2032
Rhodopseudomonas palustris DX-1
Rhodopseudomonas palustris TIE-1
Phaeospirillum molischianum DSM 120
Desulfovibrio vulgaris str. Hildenborough
Sutterella sp. CAG:521
Rhodospirillum photometricum DSM 122
Caloramator australicus
Alkaliphilus oremlandii OhILAs
Coprothermobacter proteolyticus DSM 5265
Thermoanaerobacter pseudethanolicus ATCC 33223
1
0.64
0.93
0.58
0.99
0.65
0.73
0.77
0.75
0.7
1
1
0.9
1
0.71
0.61
0.92
1
1
0.99
1
0.72
1
1
0.54
1
0.68
0.99
1
1
0.2

Cia2

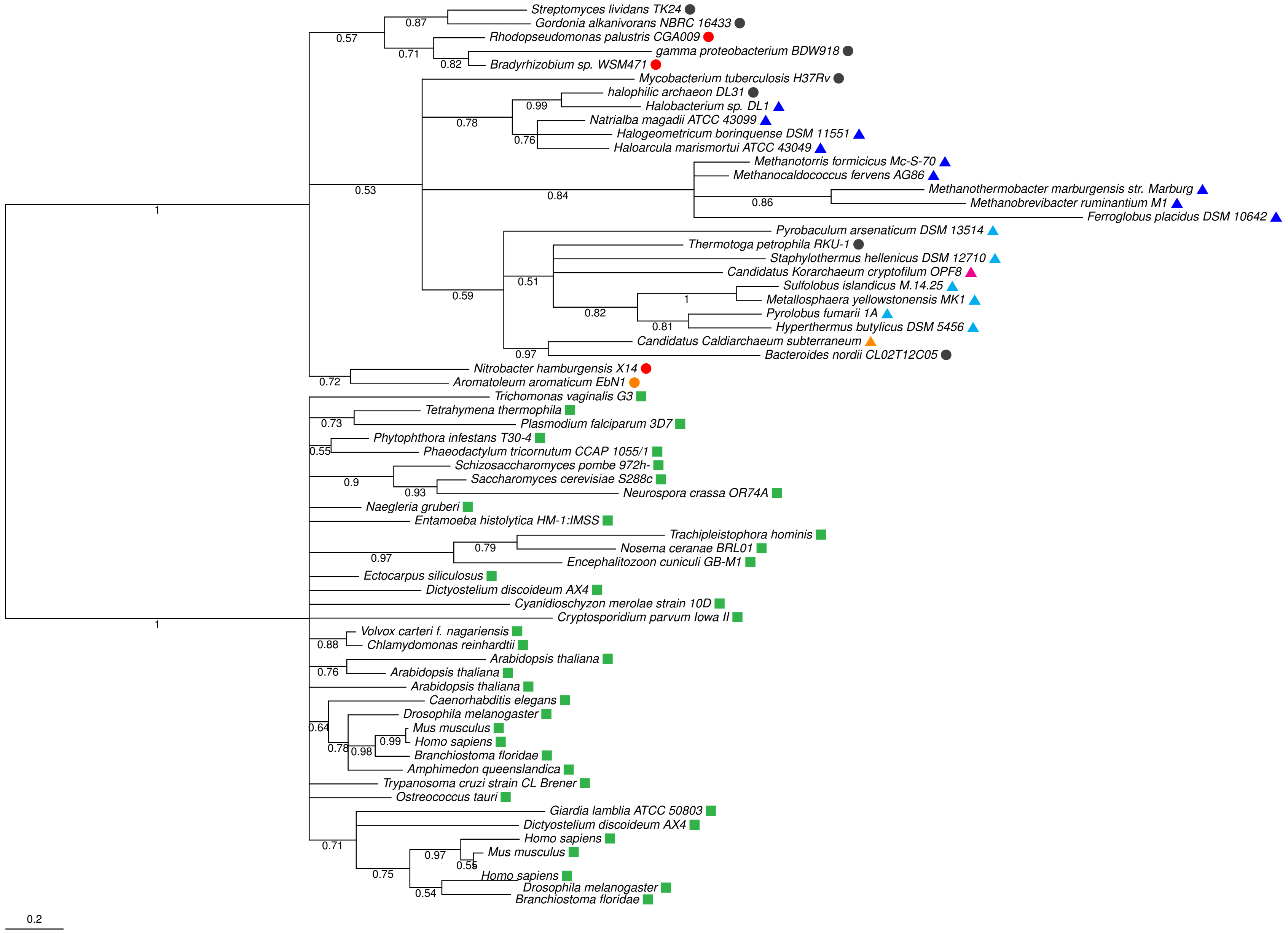

Note that the region that can be aligned between eukaryotes and prokaryotes is quite short for this protein (122 amino acids), and the specific relationships between the eukaryotes and any particular prokaryotic group are therefore tentative.

4 Nuclear and cytosolic Fe/S cluster-containing proteins

Rli1

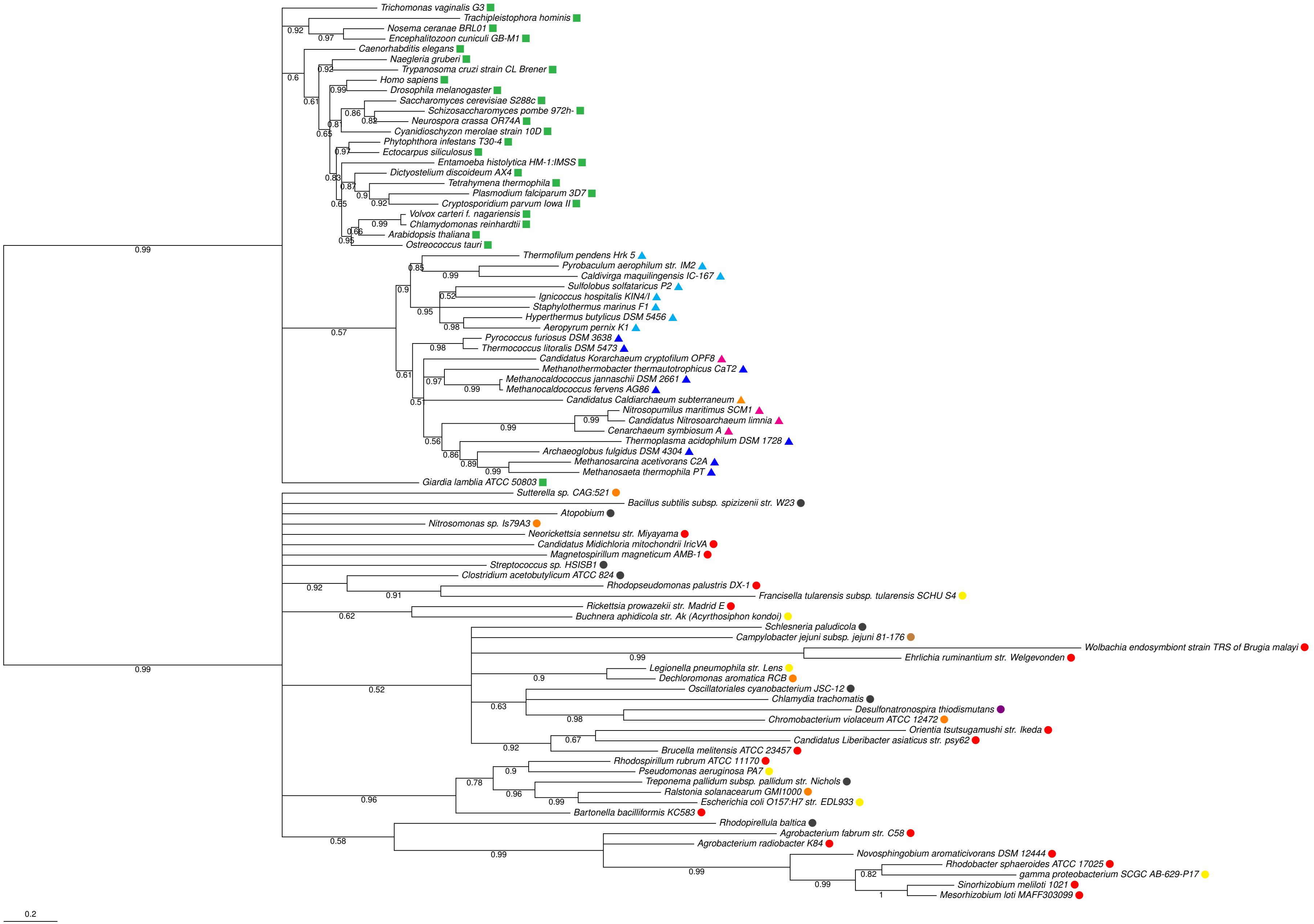

Elp3

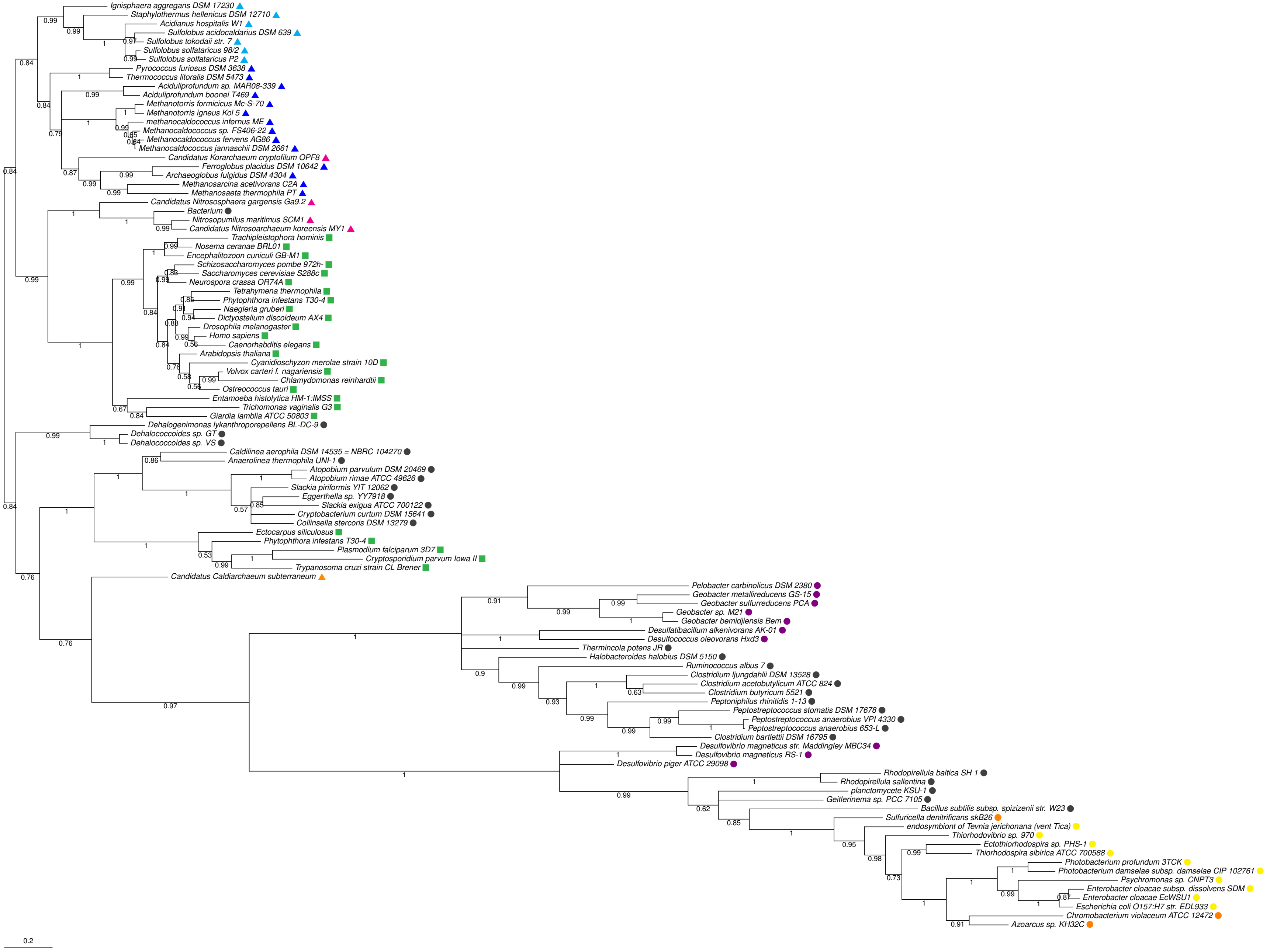

Ignisphaera aggregans DSM 17230
Staphylothermus hellenicus DSM 12710
Acidianus hospitalis W1
Sulfolobus acidocaldarius DSM 639
Sulfolobus tokodaii str. 7
Sulfolobus solfataricus 98/2
Sulfolobus solfataricus P2
Pyrococcus furiosus DSM 3638
Thermococcus litoralis DSM 5473
Aciduliprofundum sp. MAR08-339
Aciduliprofundum boonei T469
Methanotorris formicicus Mc-S-70
Methanotorris igneus Kol 5
methanocaldococcus infernus ME
Methanocaldococcus sp. FS406-22
Methanocaldococcus fervens AG86
Methanocaldococcus jannaschii DSM 2661
Candidatus Korarchaeum cryptofilum OPF8
Ferroglobus placidus DSM 10642
Archaeoglobus fulgidus DSM 4304
Methanosarcina acetivorans C2A
Methanosaeta thermophila PT
Candidatus Nitrososphaera gargensis Ga9.2
Bacterium
Nitrosopumilus maritimus SCM1
Candidatus Nitrosoarchaeum koreensis MY1
Trachipleistophora hominis
Nosema ceranae BRL01
Encephalitozoon cuniculi GB-M1
Schizosaccharomyces pombe 972h-
Saccharomyces cerevisiae S288c
Neurospora crassa OR74A
Tetrahymena thermophila
Phytophthora infestans T30-4
Naegleria gruberi
Dictyostelium discoideum AX4
Drosophila melanogaster
Homo sapiens
Caenorhabditis elegans
Arabidopsis thaliana
Cyanidioschyzon merolae strain 10D
Volvox carteri f. nagariensis
Chlamydomonas reinhardtii
Ostreococcus tauri
Entamoeba histolytica HM-1:IMSS
Trichomonas vaginalis G3
Giardia lamblia ATCC 50803
Dehalogenimonas lykanthroporepellens BL-DC-9
Dehalococcoides sp. GT
Dehalococcoides sp. VS
Caldilinea aerophila DSM 14535 = NBRC 104270
Anaerolinea thermophila UNI-1
Atopobium parvulum DSM 20469
Atopobium rimae ATCC 49626
Slackia piriformis YIT 12062
Eggerthella sp. YY7918
Slackia exigua ATCC 700122
Cryptobacterium curtum DSM 15641
Collinsella stercoris DSM 13279
Ectocarpus siliculosus
Phytophthora infestans T30-4
Plasmodium falciparum 3D7
Cryptosporidium parvum Iowa II
Trypanosoma cruzi strain CL Brener
Candidatus Caldiarchaeum subterraneum
Pelobacter carbinolicus DSM 2380
Geobacter metallireducens GS-15
Geobacter sulfurreducens PCA
Geobacter sp. M21
Geobacter bemidjiensis Bem
Desulfatibacillum alkenivorans AK-01
Desulfococcus oleovorans Hxd3
Thermincola potens JR
Halobacteroides halobius DSM 5150
Ruminococcus albus 7
Clostridium ljungdahlii DSM 13528
Clostridium acetobutylicum ATCC 824
Clostridium butyricum 5521
Peptoniphilus rhinitidis 1-13
Peptostreptococcus stomatis DSM 17678
Peptostreptococcus anaerobius VPI 4330
Peptostreptococcus anaerobius 653-L
Clostridium bartlettii DSM 16795
Desulfovibrio magneticus str. Maddingley MBC34
Desulfovibrio magneticus RS-1
Desulfovibrio piger ATCC 29098
Rhodopirellula baltica SH 1
Rhodopirellula sallentina
planctomycete KSU-1
Geitlerinema sp. PCC 7105
Bacillus subtilis subsp. spizizenii str. W23
Sulfuricella denitrificans skB26
endosymbiont of Tevnia jerichonana (vent Tica)
Thiorhodovibrio sp. 970
Ectothiorhodospira sp. PHS-1
Thiorhodospira sibirica ATCC 700588
Photobacterium profundum 3TCK
Photobacterium damselae subsp. damselae CIP 102761
Psychromonas sp. CNPT3
Enterobacter cloacae subsp. dissolvens SDM
Enterobacter cloacae EcWSU1
Escherichia coli O157:H7 str. EDL933
Chromobacterium violaceum ATCC 12472
Azoarcus sp. KH32C
0.2

Ntg1

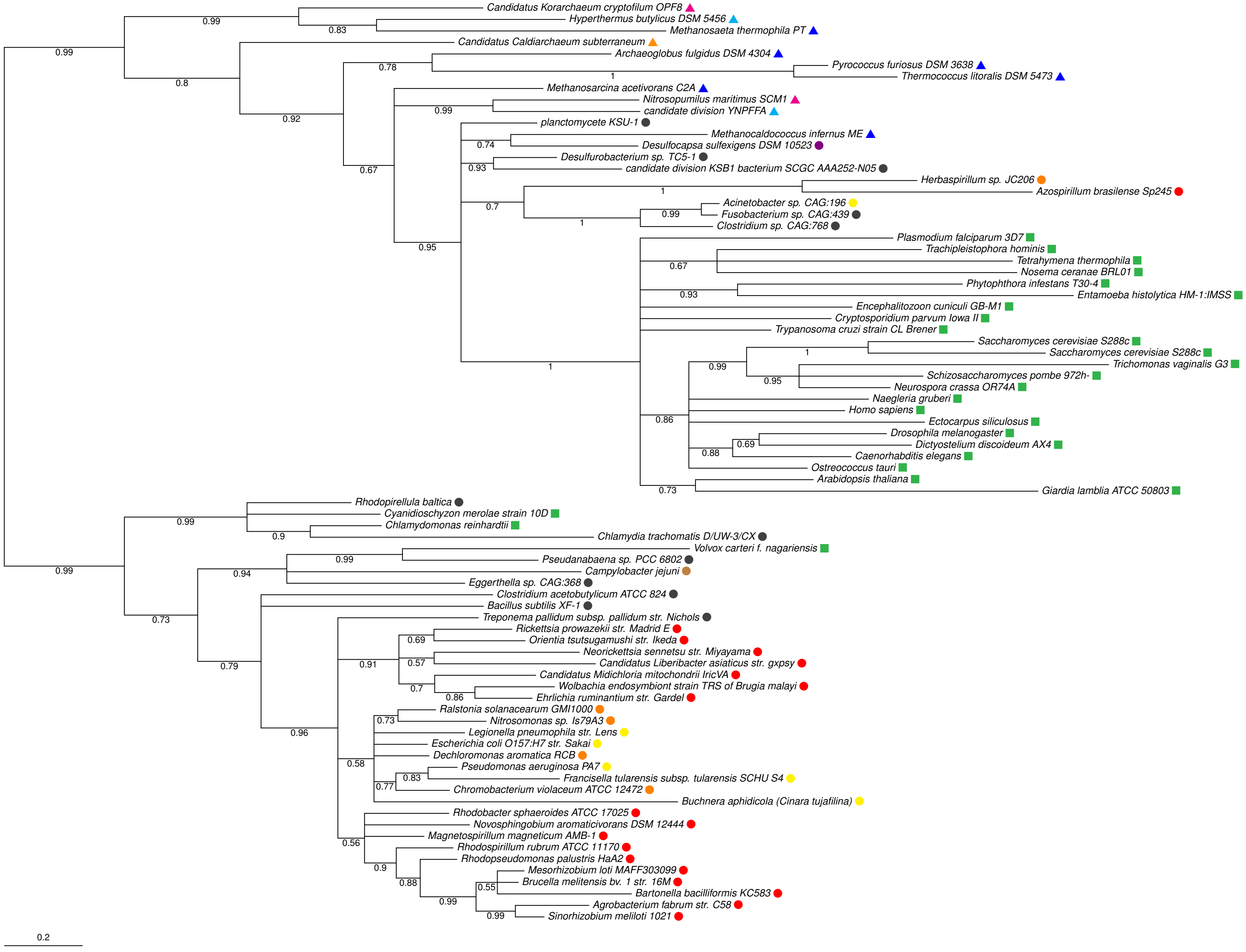
Candidatus Korarchaeum cryptofilum OPF8
Hyperthermus butylicus DSM 5456
Methanosaeta thermophila PT
Candidatus Caldiarchaeum subterraneum
Archaeoglobus fulgidus DSM 4304
Pyrococcus furiosus DSM 3638
Thermococcus litoralis DSM 5473
Methanosarcina acetivorans C2A
Nitrosopumilus maritimus SCM1
candidate division YNPFFA
planctomycete KSU-1
Methanocaldococcus infernus ME
Desulfocapsa sulfexigens DSM 10523
Desulfurobacterium sp. TC5-1
candidate division KSB1 bacterium SCGC AAA252-N05
Herbaspirillum sp. JC206
Azospirillum brasilense Sp245
Acinetobacter sp. CAG:196
Fusobacterium sp. CAG:439
Clostridium sp. CAG:768
Plasmodium falciparum 3D7
Trachipleistophora hominis
Tetrahymena thermophila
Nosema ceranae BRL01
Phytophthora infestans T30-4
Entamoeba histolytica HM-1:IMSS
Encephalitozoon cuniculi GB-M1
Cryptosporidium parvum Iowa II
Trypanosoma cruzi strain CL Brener
Saccharomyces cerevisiae S288c
Saccharomyces cerevisiae S288c
Trichomonas vaginalis G3
Schizosaccharomyces pombe 972h-
Neurospora crassa OR74A
Naegleria gruberi
Homo sapiens
Ectocarpus siliculosus
Drosophila melanogaster
Dictyostelium discoideum AX4
Caenorhabditis elegans
Ostreococcus tauri
Arabidopsis thaliana
Giardia lamblia ATCC 50803
Rhodopirellula baltica
Cyanidioschyzon merolae strain 10D
Chlamydomonas reinhardtii
Chlamydia trachomatis D/UW-3/CX
Volvox carteri f. nagariensis
Pseudanabaena sp. PCC 6802
Campylobacter jejuni
Eggerthella sp. CAG:368
Clostridium acetobutylicum ATCC 824
Bacillus subtilis XF-1
Treponema pallidum subsp. pallidum str. Nichols
Rickettsia prowazekii str. Madrid E
Orientia tsutsugamushi str. Ikeda
Neorickettsia sennetsu str. Miyayama
Candidatus Liberibacter asiaticus str. gxpsy
Candidatus Midichloria mitochondrii IricVA
Wolbachia endosymbiont strain TRS of Brugia malayi
Ehrlichia ruminantium str. Gardel
Ralstonia solanacearum GMI1000
Nitrosomonas sp. Is79A3
Legionella pneumophila str. Lens
Escherichia coli O157:H7 str. Sakai
Dechloromonas aromatica RCB
Pseudomonas aeruginosa PA7
Francisella tularensis subsp. tularensis SCHU S4
Chromobacterium violaceum ATCC 12472
Buchnera aphidicola (Cinara tujafilina)
Rhodobacter sphaeroides ATCC 17025
Novosphingobium aromaticivorans DSM 12444
Magnetospirillum magneticum AMB-1
Rhodospirillum rubrum ATCC 11170
Rhodopseudomonas palustris HaA2
Mesorhizobium loti MAFF303099
Brucella melitensis bv. 1 str. 16M
Bartonella bacilliformis KC583
Agrobacterium fabrum str. C58
Sinorhizobium meliloti 1021
0.2

Rad3/Chl1/Rtel1

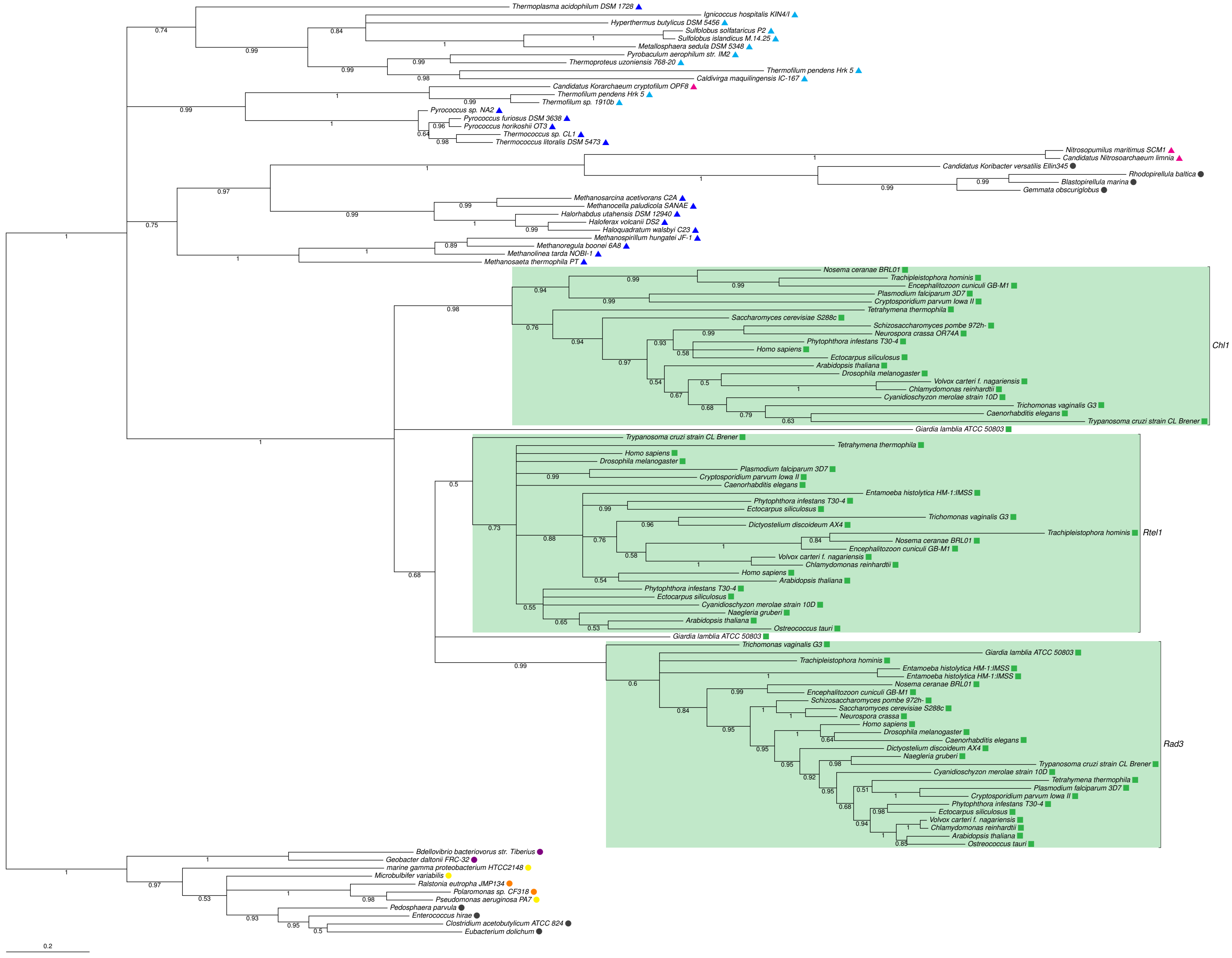

Dna2

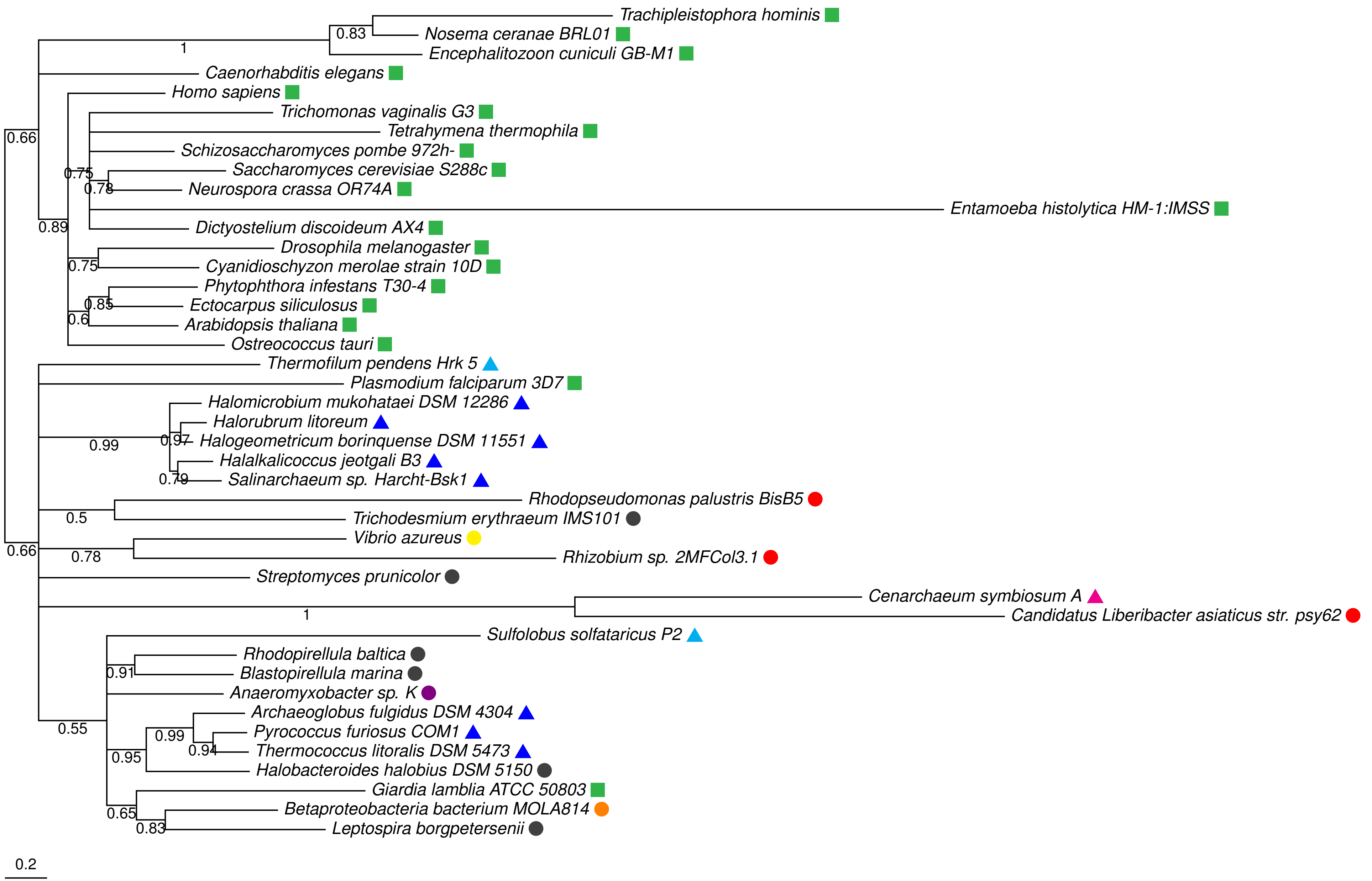

Although this tree is poorly resolved, the best BLAST hits of eukaryotic Dna2 are to sequences from the Haloarchaea; this is perhaps consistent with an archaeal origin for this gene, given the presence of good homologues in these and other Euryarchaeota. The *Giardia* and *Plasmodium* sequences included here are the most likely Dna2 candidates in these species, although the tree topology weakly excludes them from the otherwise monophyletic eukaryotic clade.

Pri2

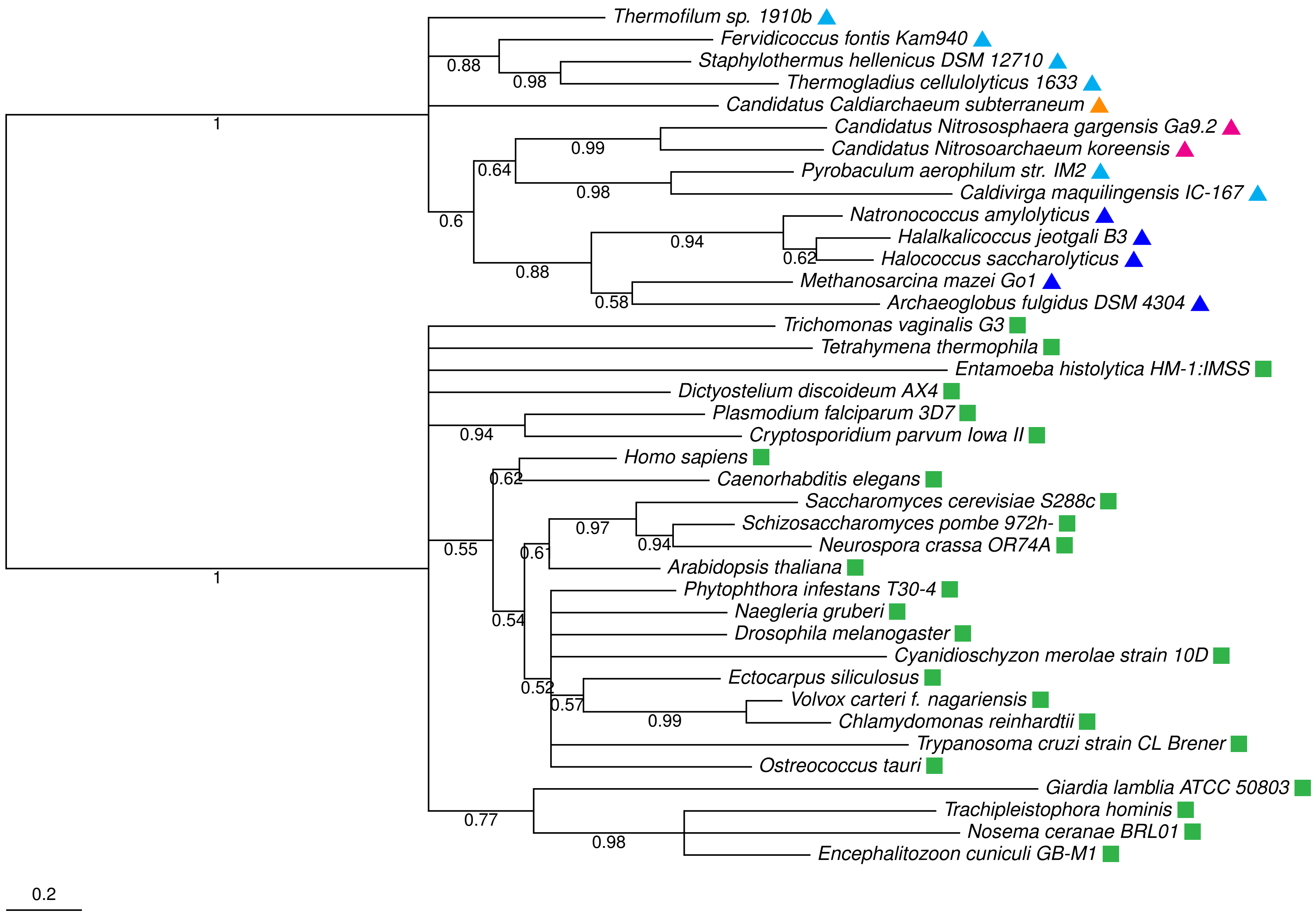

Thermofilum sp. 1910b
Fervidicoccus fontis Kam940
Staphylothermus hellenicus DSM 12710
Thermogladius cellulolyticus 1633
Candidatus Caldiarchaeum subterraneum
Candidatus Nitrososphaera gargensis Ga9.2
Candidatus Nitrosoarchaeum koreensis
Pyrobaculum aerophilum str. IM2
Caldivirga maquilingensis IC-167
Natronococcus amylolyticus
Halalkalicoccus jeotgali B3
Halococcus saccharolyticus
Methanosarcina mazei Go1
Archaeoglobus fulgidus DSM 4304
Trichomonas vaginalis G3
Tetrahymena thermophila
Entamoeba histolytica HM-1:IMSS
Dictyostelium discoideum AX4
Plasmodium falciparum 3D7
Cryptosporidium parvum Iowa II
Homo sapiens
Caenorhabditis elegans
Saccharomyces cerevisiae S288c
Schizosaccharomyces pombe 972h-
Neurospora crassa OR74A
Arabidopsis thaliana
Phytophthora infestans T30-4
Naegleria gruberi
Drosophila melanogaster
Cyanidioschyzon merolae strain 10D
Ectocarpus siliculosus
Volvox carteri f. nagariensis
Chlamydomonas reinhardtii
Trypanosoma cruzi strain CL Brener
Ostreococcus tauri
Giardia lamblia ATCC 50803
Trachipleistophora hominis
Nosema ceranae BRL01
Encephalitozoon cuniculi GB-M1
1
0.88
0.98
0.99
0.64
0.98
0.6
0.94
0.62
0.88
0.58
1
0.94
0.62
0.97
0.94
0.55
0.61
0.54
0.52
0.57
0.99
0.77
0.98
0.2

B-family DNA polymerases

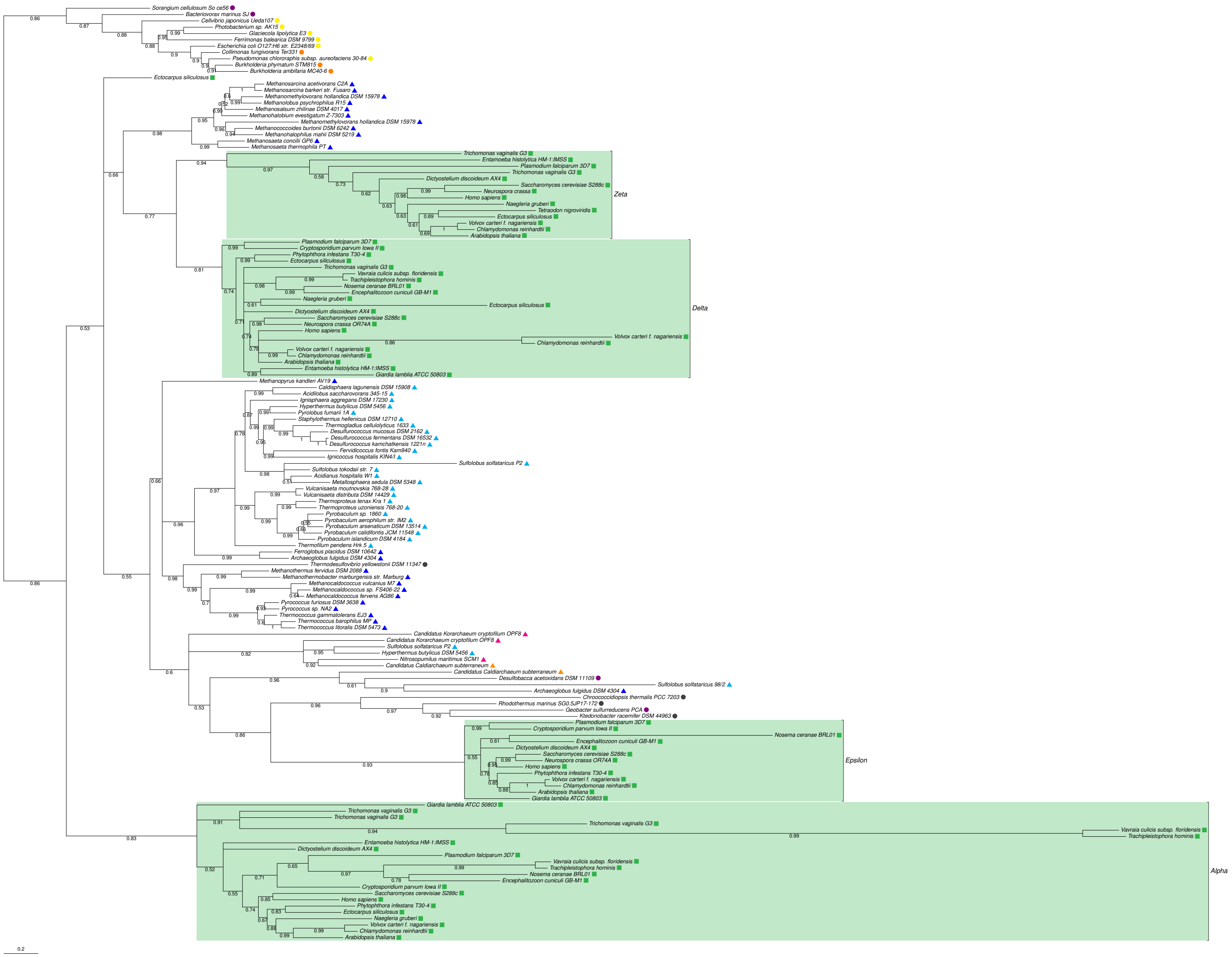

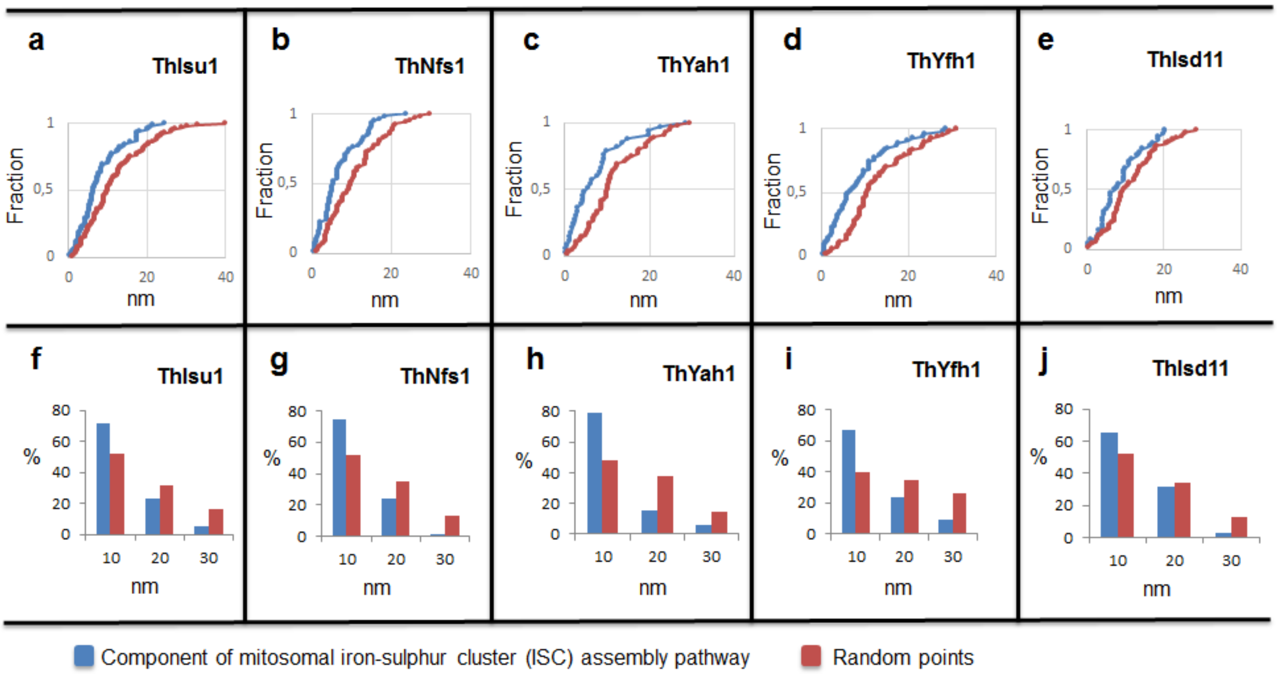

Supplementary Figure 3. Quantitative analysis of immunogold labelling for iron-sulphur cluster (ISC) assembly components over the mitosome matrix.

Distribution of immunogold labelling over mitosome matrix profiles as compared to a similar number of random points (analysed as described in the Methods). Plots (**a-e**) show the cumulative fraction (Fraction) of gold labelling (blue) and random points (red) plotted against the distance from the inner membrane (nm). For both Kolmogorov Smirnov tests and Mann-Whitney tests $p < 0.01$ for (**a-d**) and $p < 0.05$ for (**e**). The pooled data plot is shown as Fig. 1d. Plots (**f-j**) show the percentage gold labelling for ISC proteins in successive 10 nm bands from the inner membrane (Chi square test on frequency distributions for (**f-h**) $p < 0.05$, for (**i**) $p < 0.01$, and for (**j**) not significant; degrees of freedom was 2 in all cases). Compared to a random process, all tested components of the ISC pathway clearly show relative enrichment of labelling close to the mitosome inner membrane. Respective gold and random counts as follows: ThIsu1, 72/98; ThNfs1, 58/69; ThYah1 33/56; ThYfh1, 54/68 and ThIsd11, 38/62. Analyses for individual ISC proteins reveals $p < 0.01$ for ThIsu1, ThNfs1, ThYah1, and ThYfh1, and $p < 0.05$ for ThIsd11 in both Kolmogorov-Smirnov and Mann-Whitney tests.

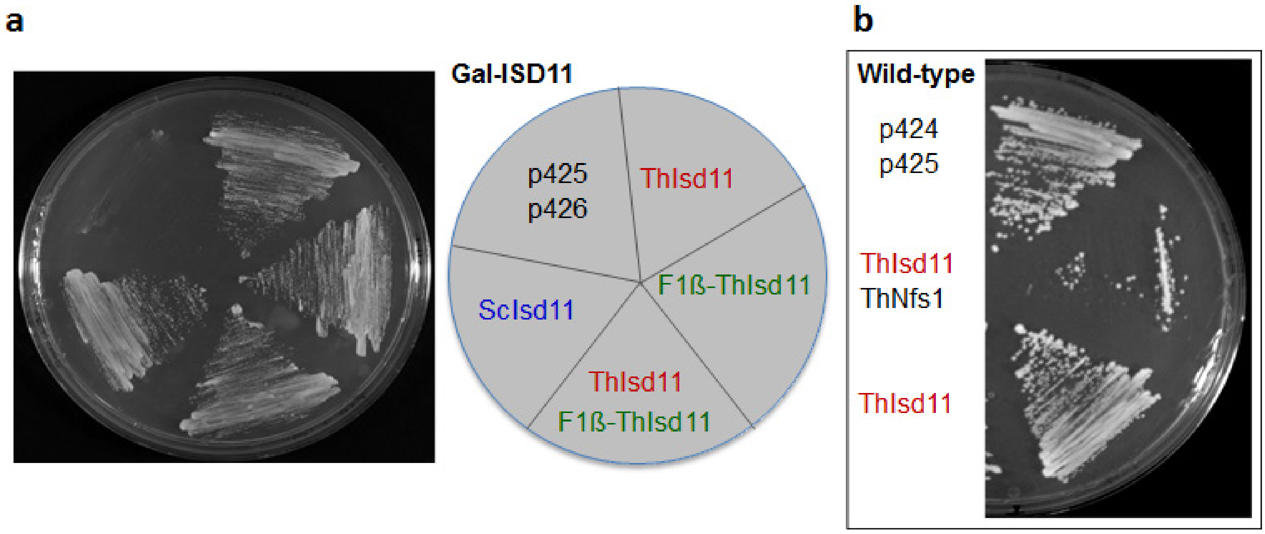

Supplementary Figure 4. Microsporidial Isd11 can complement the growth defect of a yeast Isd11-deficient mutant.

a, *T. hominis* Isd11 can rescue growth of a yeast *ISD11* mutant. Gal-*ISD11* yeast cells were transformed with the plasmids p425 and p426 containing either no insert or the *Th*ISD11 gene with or without a mitochondrial targeting sequence from *Neurospora crassa* F1-ATPase subunit β. As a positive control, yeast cells were transformed with the same plasmid containing yeast *ISD11*. Cells were depleted of endogenous Isd11 protein by growth at 30°C in minimal medium containing glucose (SD) and were spotted onto SD agar plates and grown for 2 d at 30°C. **b,** Overexpression of ThIsd11 in W303 wild-type cells does not affect growth but co-expression of ThIsd11 with ThNfs1 inhibits the growth of yeast cells. Wild-type cells were co-transformed with either the empty vectors p424 and p425, with the vectors containing Th*ISD11* and Th*NFS1* or with p424 containing Th*ISD11*. Cells were plated on minimal medium for three days at 30°C.

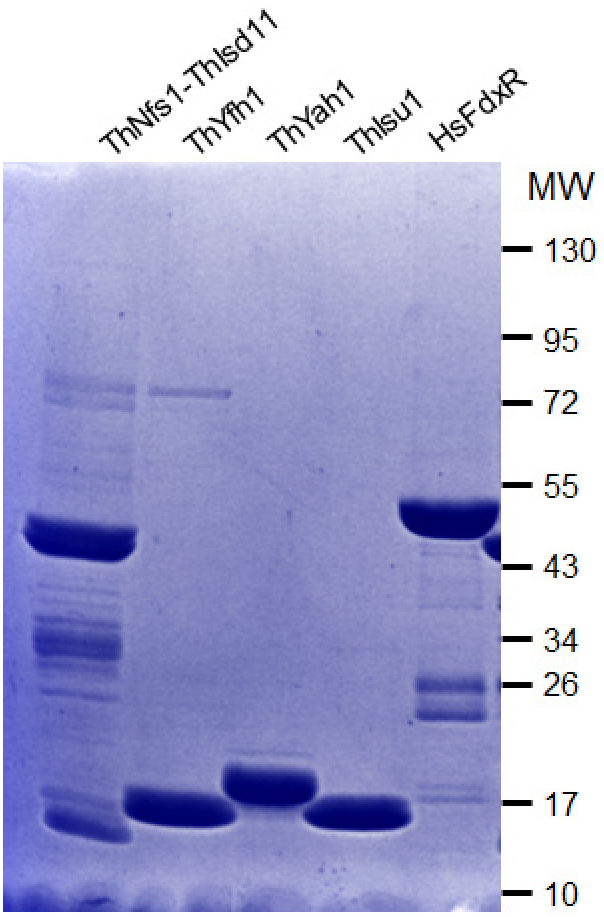

Supplementary Figure 5. Purification of recombinant *T. hominis* mitosomal ISC proteins.

The indicated components of the core mitosomal ISC assembly machinery from *T. hominis* were synthesised in *E. coli* and purified by Ni-NTA affinity chromatography and gel filtration (Äkta Purifier System 10, Column 16/60 Superdex 300). Aliquots were subjected to SDS-PAGE and proteins were stained using Coomassie.

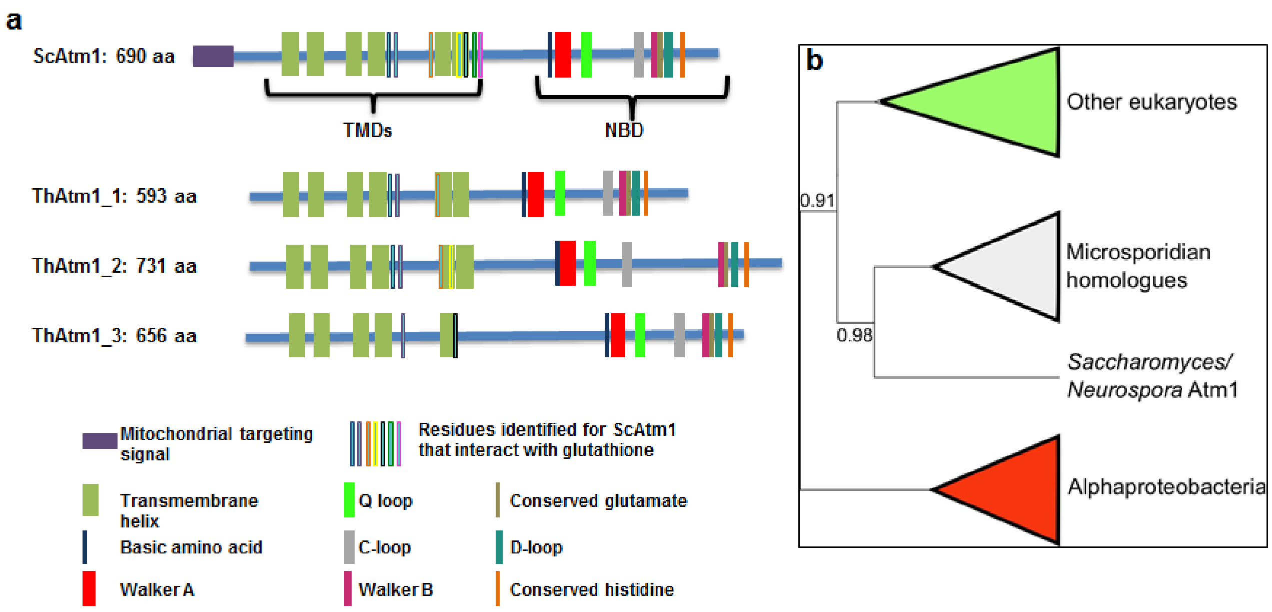

Supplementary Figure 6. Characterization of three candidate Atm1 homologues from *T. hominis.*

a, Comparison of the domain organization and sequence motifs of yeast ScAtm1 and the three best candidates for a *T. hominis* homologue. Atm1 is an ATP-binding cassette (ABC) half-transporters that contains an N-terminal mitochondrial targeting signal followed by transmembrane (TMD) and nucleotide-binding (NBD) domains[17,18]. The protein domain organization and key motifs mediating ATPase activity identified for ScAtm1 are conserved in all three *T. hominis* proteins (see alignment Supplementary Figure 1d). None of the *T. hominis* proteins have a predicted N-terminal mitochondrial targeting signal, but this has also been observed for *T. hominis* mitosomal Hsp70 which is nevertheless targeted to the organelle. ThAtm1_1 and ThAtm1_2 also contain most of the recognized glutathione-coordinating residues[25]. Other conserved motifs within the NBD include the Walker A motif and the preceding conserved basic amino acid[20]; the Q- and C- (signature) loops which are part of the ATP-binding motif[22]; the Walker B motif followed by a glutamate residue which acts as a catalytic base[18]; the D-loop which interacts with the Walker A motif[23], and a conserved histidine that is proposed to be involved in the catalytic reaction[24]. The TMDs were predicted with the TOPCONS server (http://topcons.cbr.su.se). The presence of a mitochondrial targeting signal was investigated using MITOPROT (http://ihg.gsf.de/ihg/mitoprot.html). **b,** Schematic tree showing the relationship between microsporidian Atm1 homologues, ScAtm1 and reference sequences, the detailed tree itself is included in Supplementary Figure 2.

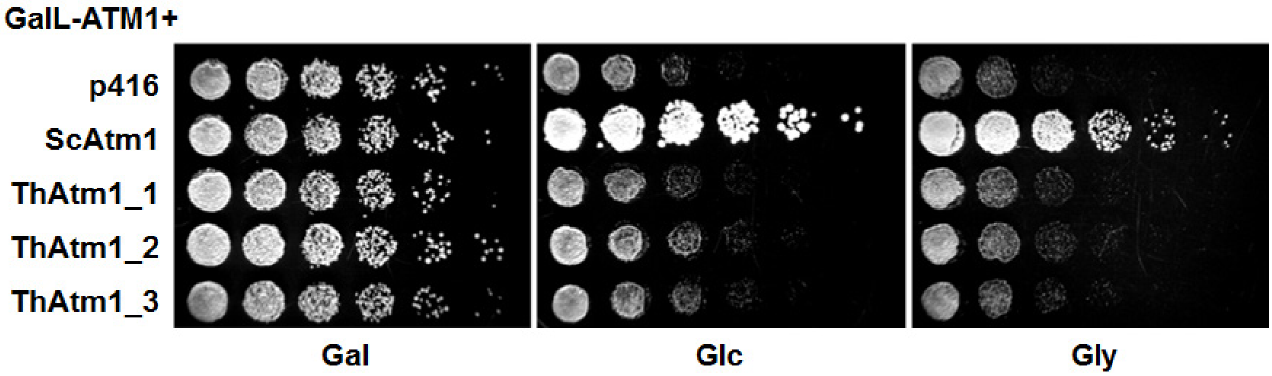

Supplementary Figure 7. Complementation of yeast GalL-ATM1 mutant cells by candidate Atm1 homologues from *T. hominis.* GalL-*ATM1* yeast cells were transformed with vector p416-MET25 containing no gene (p416), the *S. cerevisiae ATM1* gene (ScAtm1), or the three best candidate genes for ThAtm1. The ThAtm1 constructs included the coding information for a yeast N-terminal mitochondrial targeting signal. Cells were depleted of nuclear-encoded Atm1 by growth for 32 h at 30°C in minimal medium containing glucose. Serial tenfold dilutions were spotted onto minimal medium agar plates containing the indicated carbon sources, and growth was continued for 2 d at 30°C. Gal, galactose; Glc, glucose; Gly, glycerol.

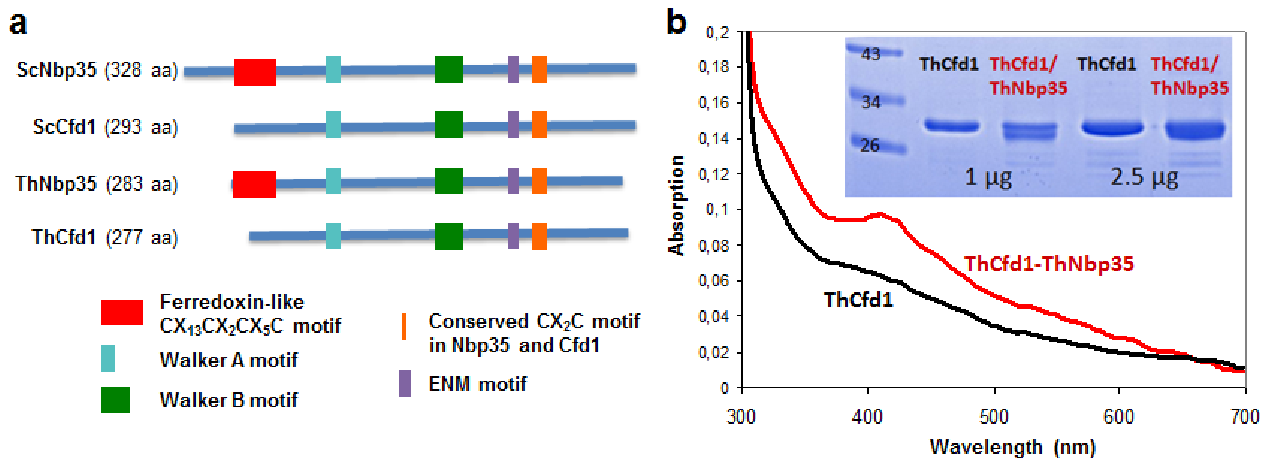

Supplementary Figure 8. Domain organization of microsporidian Cfd1 and Nbp35 homologues and chemical reconstitution. a, Conserved domain organization and sequence motifs in yeast and microsporidian Nbp35 and Cfd1 protein sequences. Yeast Nbp35 binds a [4Fe-4S] cluster to the ferredoxin-like $CX_{13}CX_2CX_5C$ motif at the N-terminus. A second [4Fe-4S] cluster binds to the conserved C-terminal CX_2C motif present in both Nbp35 and Cfd1, bridging both proteins[26,28,31,32]. Cfd1 and Nbp35 belong to the Mrp/NBP35 subfamily of P-loop NTPases. Nucleotide binding and/or hydrolysis is apparently critical for loading of Fe/S clusters onto the Cfd-Nbp35 complex. The important Walker A and B domains for nucleotide binding, and the conserved ENMS sequence common to members of the Mrp/NBP35 subfamily[48] are all conserved in the microsporidian sequences. **b,** A [4Fe-4S] cluster can be biochemically reconstituted on the *T. hominis* Cfd1-Nbp35 scaffold *in vitro*. *T. hominis* ThCdf1 or both ThCfd1 and ThNbp35 were purified after expression in *E. coli* and used for chemical reconstitution of a [4Fe-4S] cluster as measured by UV-Vis spectroscopy. The peak around 420 nm indicates the efficient formation of a [4Fe-4S] cluster on the ThCfd1-ThNbp35 complex.

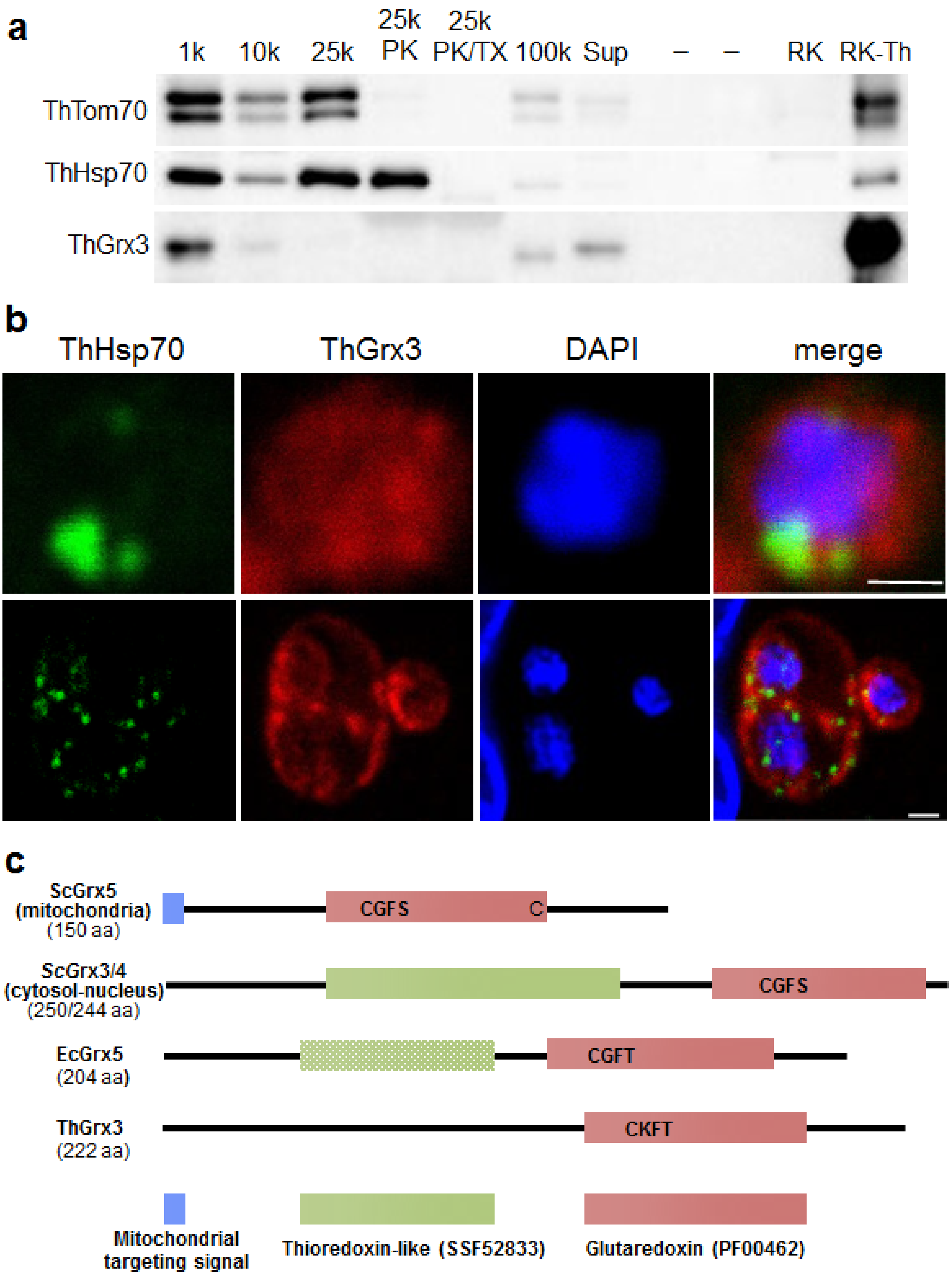

Supplementary Figure 9. Cellular localization and domain organization of the *T. hominis* Grx3 homologue. a, Western blots using antibodies to *T. hominis* proteins with fractions obtained by differential centrifugation from RK cells or RK cells infected with *T. hominis* (RK-Th) Centrifugation speeds for pellet fractions are given above each lane. Sup = final 100 K supernatant. 25 K fractions were treated with proteinase K (PK) and Triton X-100 detergent (PK-TX) as indicated.

b, Immunofluorescence microscopy of paraformaldehyde-fixed (2.5% PFA in PBS at room temperature for 10 min followed by permeabilization in 0.2% Triton X-100 in PBS for 10 min, top row) or methanol:acetone-fixed (1:1 at -20°C for 10 min, bottom row) RK cells infected with *T. hominis* (Th) using a rat antibody to *T. hominis* mitosomal Hsp70 (ThHsp70, green), and a rabbit antibody to the *T. hominis* candidate Grx3 (ThGrx3, red). DAPI (blue) was used to label host and parasite nuclear DNA. Merged images are shown on the right. Scale bars correspond to 2 µm. **c,** Comparison of the domain organization between the *S. cerevisiae* monothiol glutaredoxins Grx5 of mitochondria, Grx3-Grx4 of the cytosol-nucleus, and the glutaredoxins from *E. cuniculi* and *T. hominis*. Yeast Grx3, Grx4 and Grx5 contain a glutaredoxin (Grx) domain with the active site motif CGFS[49]. The Grx domain is present in both microsporidial proteins. Grx3-Grx4 additionally possess an N-terminal thioredoxin-like (Trx) domain with a conserved sequence (WAEPC) similar to the thioredoxin active site motif (WCGPC). The Grx proteins from both *E. cuniculi* (previously designated EcGrx5[50]) and *T. hominis* (annotated here as ThGrx3; see below) are longer than ScGrx5. EcGrx5 contains a divergent, N-terminally truncated Trx domain lacking a WAEPC-like sequence (dotted green domain; detected using the SSF52833 profile and InterProScan 4.8[51]. The ThGrx3 N terminus is 40% identical to the corresponding *E. cuniculi* domain, but a similarity to the Trx domain was not detectable upon sequence comparison.

Supplementary Table 1: Conservation of the machinery for Fe/S protein biogenesis and for nuclear and cytosolic Fe/S proteins in *Encephalitozoon cuniculi* and *Trachipleistophora hominis* and representative eukaryotes with mitochondria, mitosomes or hydrogenosomes.

		Organelle type	MT	MS		MT		MS		HY	MS	MT
			Ascomycota	Microsporidia		Human	Amoebozoa		Excavates		Apicomplexa	Plants
	**Gene**	**Description**	***Sc***	***Ec***	***Th***	***Hs***	***Dd***	***Eh***	***Gl***	***Tv***	***Cp***	***Pf***	***At***
Components of the core mitochondrial ISC assembly machinery	ISU1, ISU2	Scaffold function for de novo assembly of [2Fe-2S] clusters	■	■	■	■	■		■	■	■	■	■
	NFS1	Cysteine desulphurase; generates the sulfur moiety from cysteine	■	■	■	■	■		■	■	■	■	■
	NIFU	Scaffold function; homologue of bacterial NifU required for maturation of Fe/S clusters of bacterial nitrogenase						■					
	NIFS	Cysteine desulphurase; paralogue of Nfs1 and homologue of bacterial NifS						■					
	ISD11	Essential binding partner of the cysteine desulfurase Nfs1	■	■	■	■	■			■		■	■
	ARH1	Ferredoxin/adrenodoxin reductase; transfers electrons from NADPH to ferredoxin YAH1	■	■	■	■	■				■	■	■
	YAH1	Ferredoxin; part of electron transfer chain required for the synthesis of the [2Fe-2S] cluster on ISU1	■	■	■	■	■	■	■	■	■	■	■
	YFH1	Frataxin; potential iron chaperone or stimulator of sulfur transfer from NFS1 to ISU1	■	■	■	■	■			■	■		■
	SSQ1	Hsp70 protein; involved in Fe/S cluster transfer from ISU1 to GRX5	■	■	■	■	■	■	■	■	■	■	■
	JAC1	Specialised J-protein chaperone that stimulates the ATPase function of SSQ1	■	■	■	■	■		■	■	■	■	■
	MGE1	Nucleotide exchange factor for the Ssq1 ATPase; homologue of bacterial GrpE	■			■	■		■	■	■	■	■
	GRX5	Glutathione-dependent binding of [2Fe-2S] cluster; involved in Fe/S cluster transfer	■			■	■		■			■	■
Maturation of mitochondrial [4Fe-4S] proteins	IBA57	Involved in the maturation of mitochondrial [4Fe-4S] proteins	■			■	■					■	■
	ISA1	Involved in the maturation of mitochondrial [4Fe-4S] proteins	■			■	■		■	■		■	■
	ISA2	Involved in the maturation of mitochondrial [4Fe-4S] proteins	■			■	■					■	■
	NFU1	Involved in the maturation of aconitase, lipoate synthase and respiratory complexes I and II	■			■	■		■	■		■	■
	BOL3	Involved in the maturation of lipoate synthase and respiratory complexes I and II	■			■	■				■	■	■
	IND1	Required for the Fe/S cluster assembly of complex I				■	■			■	■		■
Export	ATM1	Mitochondrial inner membrane ABC transporter; transfers sulfur-containing compound to CIA machinery	■	■	■	■	■	■	■	■	■	■	■
	ERV1	Sulphydryl oxidase of the intermembrane space	■			■	■						■
Components of the core cytosolic Fe/S cluster assembly machinery (CIA)	TAH18	Diflavin oxidoreductase; forms a complex with Dre2 and delivers electrons from NADPH to the [2Fe-2S] cluster of Dre2	■	■	■	■	■		■	■	■	■	■
	DRE2*	Forms a complex with Tah18 and receives electrons from NADPH via Tah18	■	■	■	■	■				■	■	■
	CFD1, NBP35*	Form the cytosolic Fe/S scaffold complex that assembles [4Fe-4S] clusters	■	■	■	■	■	■	■	■	■	■	■
	NAR1*	Iron-only hydrogenase-like protein; assists Fe/S cluster transfer from Cfd1-Nbp35 to target apoproteins	■	■	■	■	■	■	■	■	■	■	■
	GRX3	Glutathione-dependent binding of [2Fe-2S] clusters; involved in cellular iron metabolism	■	■	■	■	■				■	■	■
	CIA1	WD40 beta propeller protein; part of CIA targeting complex assisting Fe/S cluster insertion into target apoproteins	■	■	■	■	■	■	■	■	■	■	■
	CIA2/ MIP18	DUF59 domain protein; part of CIA targeting complex assisting Fe/S cluster insertion into target apoproteins	■	■	■	■	■	■	■	■	■	■	■
	MMS19/ MET18	Part of CIA targeting complex assisting Fe/S cluster insertion into target apoproteins	■			■	■	■					■
	YAE1	Forms adapter complex with LTO1 to assist Fe/S cluster insertion into RLI1; binds to CIA targeting complex	■			■	■						■
	LTO1	Forms adapter complex with YAE1 to assist Fe/S cluster insertion into RLI1; binds to CIA targeting complex	■			■							■
Nuclear and cytosolic Fe/S containing proteins	RLI1	'RNAse L inhibitor'; required for ribosome biogenesis and translation initiation and termination (80S particle dissociation)	■	■	■	■	■	■	■	■	■	■	■
	PPAT/ GPAT/ CAB4	Phosphoribosyl pyrophosphate amido-transferase	■		■	■	■	■	■	■	■		■
	ELP3	Subunit of Elongator complex; required for post-transcriptional modification of nucleosides in tRNA	■	■	■	■	■	■	■	■	■	■	■
	NTG1, NTG2	DNA N-glycosylase and apurinic/apyrimidinic (AP) lyase; involved in base excision repair	■	■	■	■	■	■	■	■	■	■	■
	RAD3	DNA helicase; involved in nucleotide excision repair and transcription	■	■	■	■	■	■	■	■	■	■	■
	CHL1	DNA helicase; involved in sister-chromatid cohesion and genome integrity and interstrand cross-link repair	■	■	■	■			■	■	■	■	■
	DNA2	Tripartite DNA replication factor; involved in DNA replication and repair	■	■	■	■	■	■		■			■
	RTEL1	DNA helicase; plays a critical role in telomere maintenance and DNA repair		■	■	■	■	■	■	■	■	■	■
	PRI2	Subunit of DNA primase; required for DNA synthesis and double-strand break repair	■	■	■	■	■	■	■	■	■	■	■
	POL1	DNA polymerase alpha, catalytic subunit	■	■	■	■	■	■	■	■	■	■	■
	POL2	DNA polymerase epsilon, catalytic subunit A	■	■		■	■		■		■	■	■
	POL3	DNA polymerase delta, catalytic subunit	■	■	■	■	■	■	■	■	■	■	■

Mitochondrial/Mitosomal ISC pathway — Cytosolic Fe/S protein assembly — Nuclear and cytosolic Fe/S proteins

*** denotes components of the Fe/S biogenesis pathways that are Fe/S proteins themselves.**

Key: Sc: S. cerevisiae; Ec: E. cuniculi; Th: T. hominis; Hs: H. sapiens; Dd: D. discoideum; Eh: E. histolytica; Gl: G. lamblia; Tv: T. vaginalis; Cp: C. parvum; Pf: P. falciparum; At: A. thaliana. MT: mitochondria; MS: mitosomes; HY: hydrogenosomes.

Supplementary Table 2. Functional complementation of yeast ISC and CIA depletion cells by *Trachipleistophora hominis* genes

	T. hominis gene	Complementation in yeast	Source of data
Components of the core mitochondrial ISC assembly machinery	ISU1	Yes	Goldberg et al. 2008 (Ref. 50)
	NFS1	No	Goldberg et al. 2008 (Ref. 50)
	HSP70	No (dominant negative in yeast)	Goldberg et al. 2008 (Ref. 50)
	YFH1	No	Goldberg et al. 2008 (Ref. 50); this study
	ISD11	Yes	This study
	NFS1+ISD11	No (dominant negative in yeast)	This study
	ARH1	No	This study
	YAH1	No	This study
	ARH1+YAH1	No	This study
	GRX3	No complementation of Grx5	This study
	ATM1_1	No	This study
	ATM1_2	No	This study
	ATM1_3	No	This study
Components of the core cytosolic Fe/S cluster assembly machinery (CIA)	CFD1	No	This study
	NBP35	No	This study
	CFD1+NBP35	No	This study
	NAR1	No	This study
	CIA1	No	This study

Supplementary References:

1 Ziegler, G. A. & Schulz, G. E. Crystal structures of adrenodoxin reductase in complex with NADP+ and NADPH suggesting a mechanism for the electron transfer of an enzyme family. *Biochemistry* **39**, 10986-10995 (2000).

2 Ziegler, G. A., Vonrhein, C., Hanukoglu, I. & Schulz, G. E. The structure of adrenodoxin reductase of mitochondrial P450 systems: electron transfer for steroid biosynthesis. *J Mol Biol* **289**, 981-990, (1999).

3 Aliverti, A., Pandini, V., Pennati, A., de Rosa, M. & Zanetti, G. Structural and functional diversity of ferredoxin-NADP(+) reductases. *Arch Biochem Biophys* **474**, 283-291 (2008).

4 Muller, J. J., Lapko, A., Bourenkov, G., Ruckpaul, K. & Heinemann, U. Adrenodoxin reductase-adrenodoxin complex structure suggests electron transfer path in steroid biosynthesis. *J Biol Chem* **276**, 2786-2789 (2001).

5 Brandt, M. E. & Vickery, L. E. Charge pair interactions stabilizing ferredoxin-ferredoxin reductase complexes. Identification by complementary site-specific mutations. *J Biol Chem* **268**, 17126-17130 (1993).

6 Vickery, L. E. Molecular recognition and electron transfer in mitochondrial steroid hydroxylase systems. *Steroids* **62**, 124-127 (1997).

7 Kakuta, Y., Horio, T., Takahashi, Y. & Fukuyama, K. Crystal structure of Escherichia coli Fdx, an adrenodoxin-type ferredoxin involved in the assembly of iron-sulfur clusters. *Biochemistry* **40**, 11007-11012 (2001).

8 Sainz, G. *et al.* Structure of a [2Fe-2S] ferredoxin from Rhodobacter capsulatus likely involved in Fe-S cluster biogenesis and conformational changes observed upon reduction. *J Biol Inorg Chem* **11**, 235-246 (2006).

9 Coghlan, V. M. & Vickery, L. E. Site-specific mutations in human ferredoxin that affect binding to ferredoxin reductase and cytochrome P450scc. *J Biol Chem* **266**, 18606-18612 (1991).

10 Iwema, T. *et al.* Structural basis for delivery of the intact [Fe2S2] cluster by monothiol glutaredoxin. *Biochemistry* **48**, 6041-6043 (2009).

11 Johansson, C. *et al.* The crystal structure of human GLRX5: iron-sulfur cluster co-ordination, tetrameric assembly and monomer activity. *Biochem J* **433**, 303-311 (2011).

12 Li, H. *et al.* The yeast iron regulatory proteins Grx3/4 and Fra2 form heterodimeric complexes containing a [2Fe-2S] cluster with cysteinyl and histidyl ligation. *Biochemistry* **48**, 9569-9581 (2009).

13 Picciocchi, A., Saguez, C., Boussac, A., Cassier-Chauvat, C. & Chauvat, F. CGFS-type monothiol glutaredoxins from the cyanobacterium Synechocystis PCC6803 and other evolutionary distant model organisms possess a glutathione-ligated [2Fe-2S] cluster. *Biochemistry* **46**, 15018-15026 (2007).

14 Belli, G. *et al.* Structure-function analysis of yeast Grx5 monothiol glutaredoxin defines essential amino acids for the function of the protein. *J Biol Chem* **277**, 37590-37596 (2002).

15 Soding, J., Biegert, A. & Lupas, A. N. The HHpred interactive server for protein homology detection and structure prediction. *Nucleic Acids Res* **33**, W244-248 (2005).

16 Marchler-Bauer, A. *et al.* CDD: NCBI's conserved domain database. *Nucleic Acids Res* **43**, D222-226 (2015).

17 Kispal, G., Csere, P., Prohl, C. & Lill, R. The mitochondrial proteins Atm1p and Nfs1p are essential for biogenesis of cytosolic Fe/S proteins. *Embo J* **18**, 3981-3989 (1999).

18 Leighton, J. & Schatz, G. An ABC transporter in the mitochondrial inner membrane is required for normal growth of yeast. *Embo J* **14**, 188-195 (1995).

19 Tsirigos, K. D., Peters, C., Shu, N., Kall, L. & Elofsson, A. The TOPCONS web server for consensus prediction of membrane protein topology and signal peptides. *Nucleic Acids Res* **43**, W401-407 (2015).

20 Chloupkova, M., Reaves, S. K., LeBard, L. M. & Koeller, D. M. The mitochondrial ABC transporter Atm1p functions as a homodimer. *FEBS Lett* **569**, 65-69 (2004).

21 Smith, P. C. *et al.* ATP binding to the motor domain from an ABC transporter drives formation of a nucleotide sandwich dimer. *Mol Cell* **10**, 139-149 (2002).

22 Schmitt, L. & Tampe, R. Structure and mechanism of ABC transporters. *Curr Opin Struct Biol* **12**, 754-760 (2002).

23 Hanekop, N., Zaitseva, J., Jenewein, S., Holland, I. B. & Schmitt, L. Molecular insights into the mechanism of ATP-hydrolysis by the NBD of the ABC-transporter HlyB. *FEBS Lett* **580**, 1036-1041 (2006).

24 Zaitseva, J., Jenewein, S., Jumpertz, T., Holland, I. B. & Schmitt, L. H662 is the linchpin of ATP hydrolysis in the nucleotide-binding domain of the ABC transporter HlyB. *Embo J* **24**, 1901-1910 (2005).

25 Srinivasan, V., Pierik, A. J. & Lill, R. Crystal structures of nucleotide-free and glutathione-bound mitochondrial ABC transporter Atm1. *Science* **343**, 1137-1140 (2014).

26 Roy, A., Solodovnikova, N., Nicholson, T., Antholine, W. & Walden, W. E. A novel eukaryotic factor for cytosolic Fe-S cluster assembly. *Embo J* **22**, 4826-4835 (2003).

27 Hausmann, A. *et al.* The eukaryotic P loop NTPase Nbp35: an essential component of the cytosolic and nuclear iron-sulfur protein assembly machinery. *Proceedings of the National Academy of Sciences of the United States of America* **102**, 3266-3271 (2005).

28 Netz, D. J. *et al.* A bridging [4Fe-4S] cluster and nucleotide binding are essential for function of the Cfd1-Nbp35 complex as a scaffold in iron-sulfur protein maturation. *J Biol Chem* **287**, 12365-12378 (2012).

29 Netz, D. J. *et al.* Tah18 transfers electrons to Dre2 in cytosolic iron-sulfur protein biogenesis. *Nature chemical biology* **6**, 758-765 (2010).

30 Tsaousis, A. D., Gentekaki, E., Eme, L., Gaston, D. & Roger, A. J. Evolution of the cytosolic iron-sulfur cluster assembly machinery in Blastocystis species and other microbial eukaryotes. *Eukaryot Cell* **13**, 143-153 (2014).

31 Netz, D. J., Pierik, A. J., Stumpfig, M., Muhlenhoff, U. & Lill, R. The Cfd1-Nbp35 complex acts as a scaffold for iron-sulfur protein assembly in the yeast cytosol. *Nat Chem Biol* **3**, 278-286 (2007).

32 Leipe, D. D., Wolf, Y. I., Koonin, E. V. & Aravind, L. Classification and evolution of P-loop GTPases and related ATPases. *J Mol Biol* **317**, 41-72 (2002).

33 Li, D. & Roberts, R. WD-repeat proteins: structure characteristics, biological function, and their involvement in human diseases. *Cell Mol Life Sci* **58**, 2085-2097 (2001).

34 Neer, E. J., Schmidt, C. J., Nambudripad, R. & Smith, T. F. The ancient regulatory-protein family of WD-repeat proteins. *Nature* **371**, 297-300 (1994).

35 Smith, T. F., Gaitatzes, C., Saxena, K. & Neer, E. J. The WD repeat: a common architecture for diverse functions. *Trends in biochemical sciences* **24**, 181-185 (1999).

36 Srinivasan, V. *et al.* Structure of the yeast WD40 domain protein Cia1, a component acting late in iron-sulfur protein biogenesis. *Structure* **15**, 1246-1257 (2007).

37 Weerapana, E. *et al.* Quantitative reactivity profiling predicts functional cysteines in proteomes. *Nature* **468**, 790-795 (2010).

38 Stehling, O. *et al.* Human CIA2A-FAM96A and CIA2B-FAM96B integrate iron homeostasis and maturation of different subsets of cytosolic-nuclear iron-sulfur proteins. *Cell Metab* **18**, 187-198 (2013).

39 Stehling, O. *et al.* MMS19 Assembles Iron-Sulfur Proteins Required for DNA Metabolism and Genomic Integrity. *Science* **337**, 195-199 (2012).

40 Horner, D. S., Heil, B., Happe, T. & Embley, T. M. Iron hydrogenases--ancient enzymes in modern eukaryotes. *Trends in Biochemical Sciences* **27**, 148-153 (2002).

41 Balk, J., Pierik, A. J., Netz, D. J., Muhlenhoff, U. & Lill, R. The hydrogenase-like Nar1p is essential for maturation of cytosolic and nuclear iron-sulphur proteins. *Embo J* **23**, 2105-2115 (2004).

42 Urzica, E., Pierik, A. J., Muhlenhoff, U. & Lill, R. Crucial role of conserved cysteine residues in the assembly of two iron-sulfur clusters on the CIA protein Nar1. *Biochemistry* **48**, 4946-4958 (2009).

43 Zhang, Y. *et al.* Dre2, a conserved eukaryotic Fe/S cluster protein, functions in cytosolic Fe/S protein biogenesis. *Mol Cell Biol* **28**, 5569-5582 (2008).

44 Netz, D. J. *et al.* The conserved protein Dre2 uses essential [2Fe-2S] and [4Fe-4S] clusters for its function in cytosolic iron-sulfur protein assembly. *Biochem J* **473**, 2073-2085 (2016).

45 Soler, N. *et al.* A S-adenosylmethionine methyltransferase-like domain within the essential, Fe-S-containing yeast protein Dre2. *Febs J* **279**, 2108-2119 (2012).

46 Murataliev, M. B., Feyereisen, R. & Walker, F. A. Electron transfer by diflavin reductases. *Biochim Biophys Acta* **1698**, 1-26 (2004).

47 Porter, T. D. & Kasper, C. B. Coding nucleotide sequence of rat NADPH-cytochrome P-450 oxidoreductase cDNA and identification of flavin-binding domains. *Proceedings of the National Academy of Sciences of the United States of America* **82**, 973-977 (1985).

48 Sharma, A. K., Pallesen, L. J., Spang, R. J. & Walden, W. E. Cytosolic iron-sulfur cluster assembly (CIA) system: factors, mechanism, and relevance to cellular iron regulation. *J Biol Chem* **285**, 26745-26751 (2010).

49 Hoffmann, B. *et al.* The multidomain thioredoxin-monothiol glutaredoxins represent a distinct functional group. *Antioxid Redox Signal* **15**, 19-30 (2011).

50 Goldberg, A. V. *et al.* Localization and functionality of microsporidian iron-sulphur cluster assembly proteins. *Nature* **452**, 624-628 (2008).

51 Zdobnov, E. M. & Apweiler, R. InterProScan--an integration platform for the signature-recognition methods in InterPro. *Bioinformatics* **17**, 847-848 (2001).
